# Long-term trends in storm surge climate derived from an ensemble of global surge reconstructions

**DOI:** 10.1038/s41598-022-17099-x

**Published:** 2022-08-03

**Authors:** Michael Getachew Tadesse, Thomas Wahl, Md Mamunur Rashid, Sönke Dangendorf, Alejandra Rodríguez-Enríquez, Stefan Andreas Talke

**Affiliations:** 1 Civil, Environmental, and Construction Engineering & National Center for Integrated Coastal Research, University of Central Florida, Orlando, USA; 2 Department for River-Coastal Science and Engineering, Tulane University, New Orleans, LA USA; 3 Civil and Environmental Engineering, California Polytechnic State University, San Luis Obispo, CA USA; 4 Global Science, The Nature Conservancy, 4245 North Fairfax Drive, Suite 100, Arlington, USA; 5 Institute for Environmental Studies (IVM), Vrije Universiteit Amsterdam, 1081 HV, Amsterdam, The Netherlands

**Keywords:** Natural hazards, Civil engineering

## Abstract

We address the challenge, due to sparse observational records, of investigating long-term changes in the storm surge climate globally. We use two centennial and three satellite-era daily storm surge time series from the Global Storm Surge Reconstructions (GSSR) database and assess trends in the magnitude and frequency of extreme storm surge events at 320 tide gauges across the globe from 1930, 1950, and 1980 to present. Before calculating trends, we perform change point analysis to identify and remove data where inhomogeneities in atmospheric reanalysis products could lead to spurious trends in the storm surge data. Even after removing unreliable data, the database still extends existing storm surge records by several decades for most of the tide gauges. Storm surges derived from the centennial 20CR and ERA-20C atmospheric reanalyses show consistently significant positive trends along the southern North Sea and the Kattegat Bay regions during the periods from 1930 and 1950 onwards and negative trends since 1980 period. When comparing all five storm surge reconstructions and observations for the overlapping 1980–2010 period we find overall good agreement, but distinct differences along some coastlines, such as the Bay of Biscay and Australia. We also assess changes in the frequency of extreme surges and find that the number of annual exceedances above the 95th percentile has increased since 1930 and 1950 in several regions such as Western Europe, Kattegat Bay, and the US East Coast.

## Introduction

Extreme sea levels resulting in coastal flooding are mainly driven by waves, storm surges, and tides, and are influenced by changes and variability in relative mean sea level. Understanding the trends in magnitude and frequency of these drivers is crucial for an accurate assessment of present and future coastal flood risk, but it is challenging, especially at the global scale. Surge and tide information are commonly obtained from tide gauge records. Even though tide gauges provide very valuable in-situ sea-level observations, short record lengths in many locations (only 15% of tide gauges from the GESLA-2^1^ database have observations longer than 50 years) often limit robust statistical analysis and the estimation of secular trends in extreme sea levels. Moreover, the spatial distribution of available tide gauge records in South America, Africa, southeast Asia, and the Southern Hemisphere in general is sparse and they typically only cover short time periods. Existing tide gauge records can be extended through archival measurements^2–5^ or by reconstructing data using different modeling techniques (requiring atmospheric and/or oceanic reanalysis data as forcing)^6–8^. Using longer records not only allows for a more robust assessment of possible trends in extreme water levels, but also leads to a more accurate representation of return levels, which are important for coastal risk assessments, design of coastal defense infrastructure, and adaptation^2,9^.

Atmospheric reanalysis datasets result from the combination of models and observations with the implementation of data assimilation schemes to generate the state of a system as accurately as possible. Reanalysis datasets provide globally gridded atmospheric variables (e.g., sea-level pressure, winds etc.) over multiple decades or even centuries. Such information can be used for reconstructing continuous historical storm surge time series temporally and spatially where little or no observations exist^10^. For example, Cid et al.^11^ developed a 147-year long storm surge reconstruction from a data-driven model for Southeast Asia based on the 20th Century Reanalysis version 2c^12^ (20CRv2C). Similarly, Cid et al.^13^ reconstructed storm surges globally from 1871 to 2010 using the 20CRV2 reanalysis. Ji et al.^14^ developed a high spatial resolution storm surge reconstruction for southeast China using the ERA40^15^ and ERA-Interim^16^ reanalysis datasets, and Tadesse et al.^17^ presented a global reconstruction of storm surges (1836–2019) using five different atmospheric reanalyses (the centennial 20CRV3 and ERA-20C^18^, and satellite era ERA-Interim^16^, MERAA V2^19^, and ERA5^20^). Using a physics-based modelling approach, Muis et al.^21^ used data from the ERA-Interim reanalysis as forcing for a hydrodynamic model to derive a global reanalysis of storm surges and extreme sea levels for the 1979–2014 period. Employing an advanced version of the same hydrodynamic model, Muis et al.^22^ used data from the ERA5 climate reanalysis to derive a global dataset of extreme sea levels for 1979–2017. Many other studies have been conducted at the local or regional scale using different modelling techniques (data-driven or physics based) to develop storm surge hindcasts^23,24^.

Reconstructed storm surge data extending the observational records can be used to investigate trends in the storm surge climate at local, regional, and global scales. There is, however, an ongoing discussion about the merits of centennial reanalyses to study long-term climate trends. Donat et al.^25^ detected significant positive trends in storminess in western, central, and northern Europe when using the 20CR reanalysis. Wang et al.^26^ showed that for the North Atlantic European region and southeast Australia, trends in 20CR extra-tropical cyclone activity are in agreement with trends in geostrophic wind extremes from in-situ surface pressure observations. By contrast, Krueger et al.^27^ argued that the trends reported by Donat et al.^25^ are due to inconsistencies in the 20CR reanalysis related to a rapidly decreasing number of assimilated observations in the early twentieth century. In response to assertions made by Wang et al.^26^ that 20CR cyclone trends are in agreement with geostrophic wind extremes trends in the North Atlantic-European region, Krueger et al.^28^ showed that 20CR geostrophic storminess deviates strongly from the observation-based storminess before the 1940s. As a result, there is a spurious long-term trend in the 20CR geostrophic wind extremes which is not reflected in observed geostrophic wind extremes. The authors attribute the spurious trends to the inhomogeneities in the 20CR datasets prior to the 1950s. Inhomogeneities can be caused by inconsistencies in the amount and quality of data that are assimilated into the reanalysis products, including changes in the number of stations from where data is available and used, changes in measurement frequencies, relocation of stations, or instrumental changes^29^. These factors make the assessment of long-term climate trends using reanalysis data challenging.

In this study, we address this challenge and quantify trends in the reconstructed daily maximum storm surges obtained from the GSSR^17^ database along the global coastlines for the periods from 1930, 1950, and 1980 onwards. The centennial storm surge reconstructions are hereinafter referred to as G-20CR (GSSR surge reconstruction forced with the 20CRV3 reanalysis, 1836–2015) and G-E20C (GSSR surge reconstruction forced with the ERA-20C reanalysis, 1900–2010) whereas the satellite era reconstructions are G-EInt (GSSR surge reconstruction forced with ERA Interim reanalysis, 1979–2019), G-Merra (GSSR surge reconstruction forced with MERRA-2 reanalysis, 1980–2019), G-E5 (GSSR surge reconstruction forced with ERA-5 reanalysis, 1979–2019); we also create an ensemble mean of all reconstructions for the overlapping period 1980–2010 (G-EnsMean). Given the known limitations of reanalysis products which could lead to spurious trends, we first implement a Bayesian change point detection technique to identify time periods where reconstructed storm surge data shows suspicious behavior, and those time periods are excluded from further analysis.

**Figure 1:**
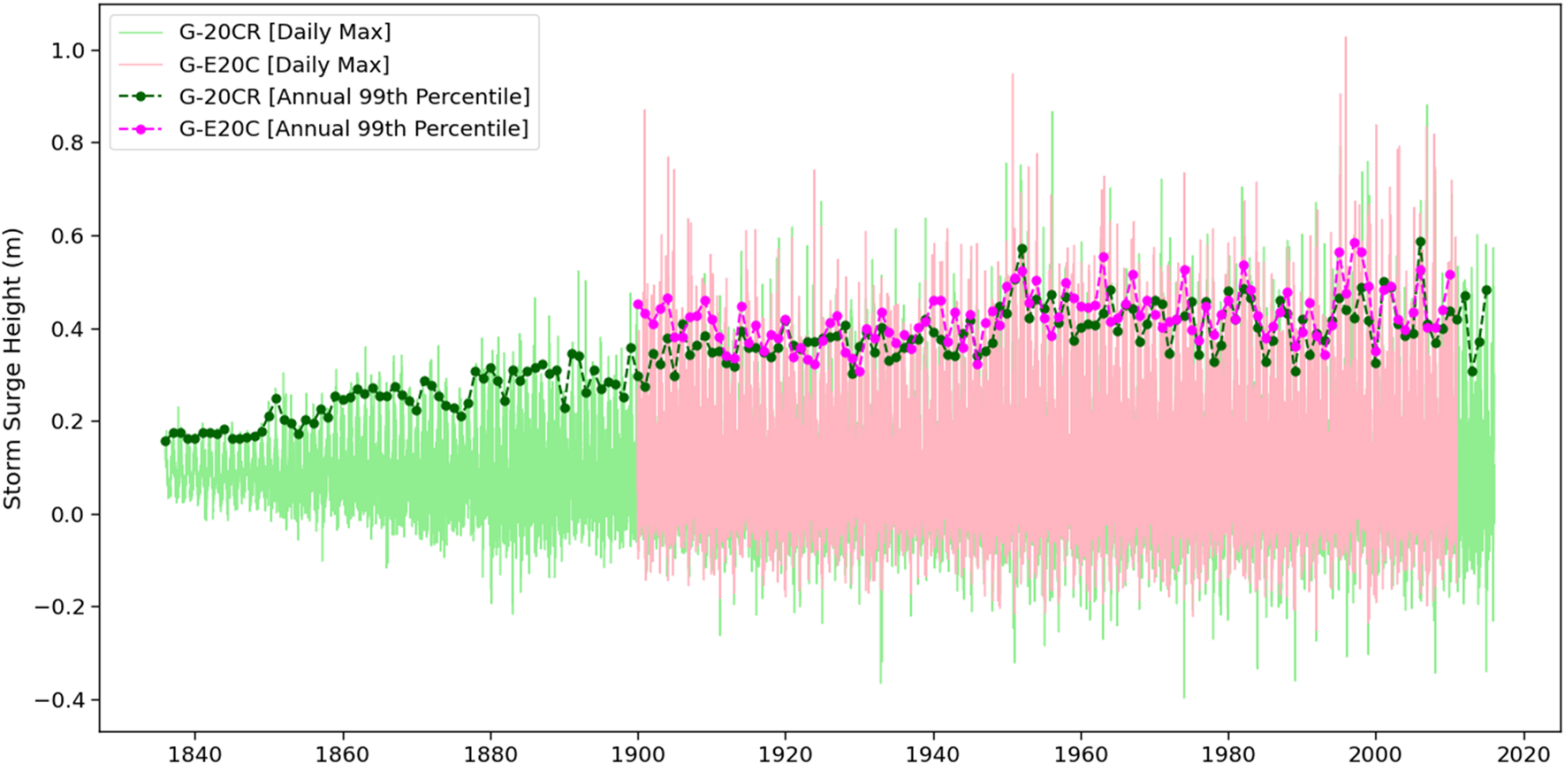
Reconstructed daily maximum surges from G-20CR (green) and G-E20C (pink) and their respective annual 99th percentiles (dashed lines with markers) for the Astoria tide gauge.

To identify time periods where modelled storm surge data is unreliable, it is preferable to validate against in-situ measurements using metrics such as the Root Mean Squared Error (RMSE) or coefficient of determination (R^2^), as shown for example in Fig. 6 of Dangendorf et al.^30^ for the Cuxhaven tide gauge. However, this can only be done for tide gauges where observed surges are long enough compared to the corresponding reconstructions. This is not the case for the vast majority of tide gauges; for example, only 10 tide gauges in GESLA-2 cover the entire twentieth century and none goes back to 1836, as G-20CR does. An alternative way to identify spurious trends, in the absence of long observational records, is to investigate only the reconstructed surge time series and the corresponding predictors used for the reconstruction. For instance, Fig. 1 shows the daily maximum surge time series and annual 99th percentile values for G-20CR and G-E20C. While there is no obvious trend in the mean of the daily maximum surge time series, both reconstructions show a persistent decrease (when looking backwards) in the variability which translates to spurious trends in the annual 99th percentile values. This is especially obvious for G-20CR where the variability declines before the 1940s and is only a fraction in the mid-nineteenth century compared to the last 80 or 90 years. Hence, the resulting increase in the 99th percentile values over time should not be interpreted as a climate related trend in storm surges, but rather as an artifact stemming from inhomogeneities in the 20CR reanalysis. This motivates us to consider the annual variability of the reconstructed surges and their predictors as a proxy for determining time periods where the quality of the surge reconstruction is poor and leads to spurious trends. A probabilistic change point detection method paired with visual inspection is employed to pre-process the reconstructed surges before trends are computed (see “Methods” for details).

## Results

### Change point detection

Based on the model validation results from Tadesse and Wahl^31^ and after applying a set of selection criteria in terms of model performance (see “Methods”), 310 and 320 tide gauges are selected with G-20CR and G-E20C surge reconstructions, respectively. These tide gauges adequately cover the Northern Hemisphere coastlines and also include several locations in the Southern Hemisphere, while the tropics are under-sampled due to model inaccuracies^47^.

**Figure 2:**
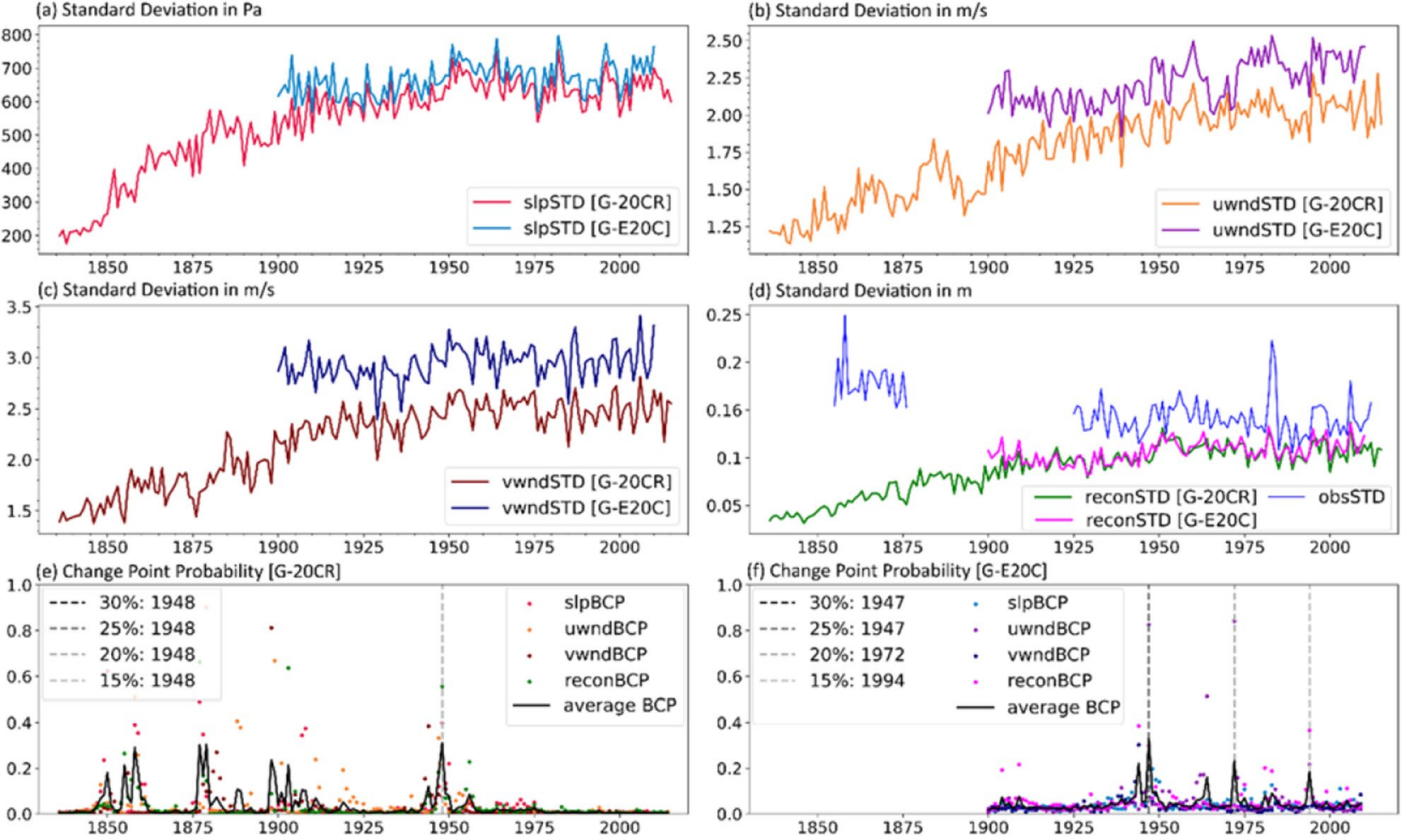
Results of change point analysis for G-20CR and G-E20C for the Astoria tide gauge. Annual variability (expressed as standard deviation) time series are shown for (**a**) sea-level pressure (slpSTD), (**b**) zonal wind speed (uwndSTD), (**c**) meridional wind speed (vwndSTD), and (**d**) reconstructed surge (reconSTD). (**e**,**f**) Bayesian change point probability (BCP) for the surge reconstruction and predictors (colored dots) and the average of them (black solid line) for G-20CR (**e**) and G-E20C (**f**); vertical dashed gray lines indicate the most recent change point year for a given cutoff probability for the average BCP.

We apply the Bayesian change point analysis for all 310 (G-20CR) and 320 (G-E20C) tide gauges on their annual variability (measured as standard deviation) time series in order to identify time periods where the data is less likely influenced by shortcomings in the reanalyses, and we only consider those time periods for the subsequent trend analysis. Figure 2 exemplarily shows the results from the Bayesian change point analysis for Astoria (US) (Fig. 2e,f), along with the annual variability of the three predictors used in Tadesse and Wahl^31^ (Fig. 2a–c), as well as the annual variability in the surge reconstructions and the observed surge (Fig. 2d). The average of the four change point probabilities corresponding to the zonal wind speed, meridional wind speed, mean sea-level pressure, and reconstructed surge are computed and presented in Fig. 2e,f. The change point detection algorithm computes the probability of each year that it constitutes a change point in the time series (see “Methods” for more details). We show here four different cut-off probabilities (probability values above which a given year is considered to be a change point) to identify likely change point years: 15%, 20%, 25%, and 30%. In the case of Astoria and for G-20CR, all cutoff probabilities indicate that the year 1948 is the most recent change point in the time series. This is also apparent from the time series shown in Fig. 2a–d. There is a rapid decrease in the annual variability of the predictors before 1948. On the other hand, for G-E20C, three probable change points (1947, 1972, and 1994) are identified for the 30%, 20%, and 15% cutoff probabilities, respectively. Visual inspection of the changes in the variability of the reanalysis predictors and reconstructed storm surge time series (Fig. 2a-d) shows a decrease in the variability of all four variables before 1947. Hence, we choose 1947 as the change point year and assume that data for the time periods 1949 to 2015 (G-20CR) and 1948 to 2010 (G-E20C) are reliable in Astoria. The same change point detection procedure has been applied for all selected G-20CR and G-E20C reconstructions (see http://gssr.info/changepoint for detailed results).

**Figure 3:**
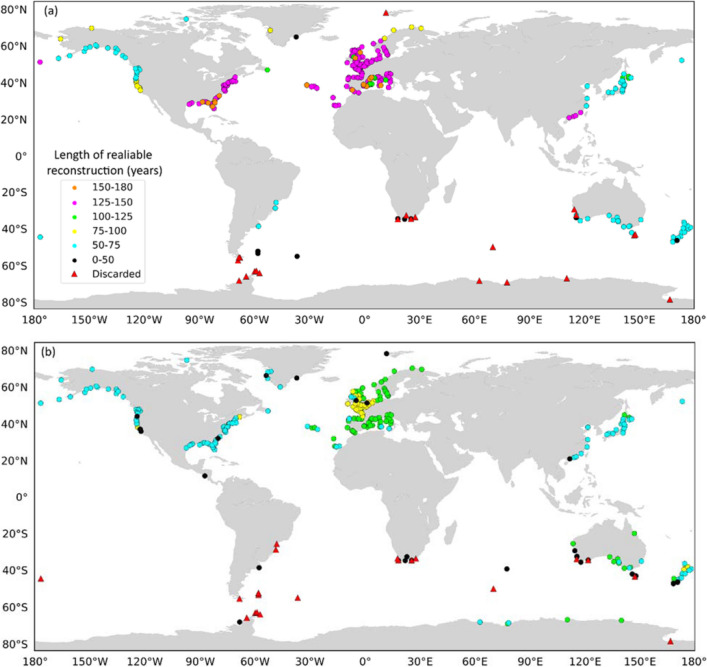
Length of G-20CR (**a**) and G-E20C (**b**) reconstructed storm surge time series in years after applying change point analysis and removing suspicious data. Red triangles represent tide gauges where surge reconstructions are rejected. Rossum, Guido van, et al., The Python Language Reference, Python Software Foundation; http://docs.python.org/py3k/reference/index.html.

After removing suspicious data from G-20CR surge reconstructions for tide gauges in southern Australia, New Zealand, Japan, and the northwest coast of the US vary in length from 50 to 75 years, and along the US Gulf coast, US East coast, and across Europe between 125 and 150 years (Fig. 3, Table 1). For several tide gauges (16 in total) along the US Gulf and East coast, Spain, Portugal, and France, G-20CR provides 150–180 years of surge reconstructions after removing suspicious data. Some tide gauges (red triangles in Fig. 3), mainly in Antarctica, southern Africa, and parts of Australia were discarded after the change point analysis due to significant (and recent) changes in the annual variabilities of predictors (see “Discussion”). For G-E20C, the lengths of the reconstructed surge time series, after removing suspicious data, for tide gauges along the US northwest coast, most of New Zealand, and Japan is 50–75 years. However, data lengths for tide gauges in southern Australia are between 100 and 110 years, which is in some cases twice as long compared to G-20CR in the same locations, pointing to distinct differences in the quality of the reanalysis data. G-E20C provides 50–75 years of data along the US Gulf and East coast, which is shorter than G-20CR. In Europe, most of the tide gauges have 100–110 years of reconstructed surge data. Similar to G-20CR, there are several tide gauges discarded in the southern polar region due to quality issues.

**Table 1:** Number of years provided/extended by each reconstruction after change point analysis.

Region	G-E20C		G-20CR	
	Total length	Observation extension [avg]	Total length	Observation extension [avg]
Europe	100–110	69	125–150	111
US East Coast + Gulf Coast	50–75	22	125–150	96
US West Coast	50–75	16	50–75	30
Japan + South East China	50–75	32	50–75	46
Australia + New Zealand	50–75	46	50–75	38

**Figure 4:**
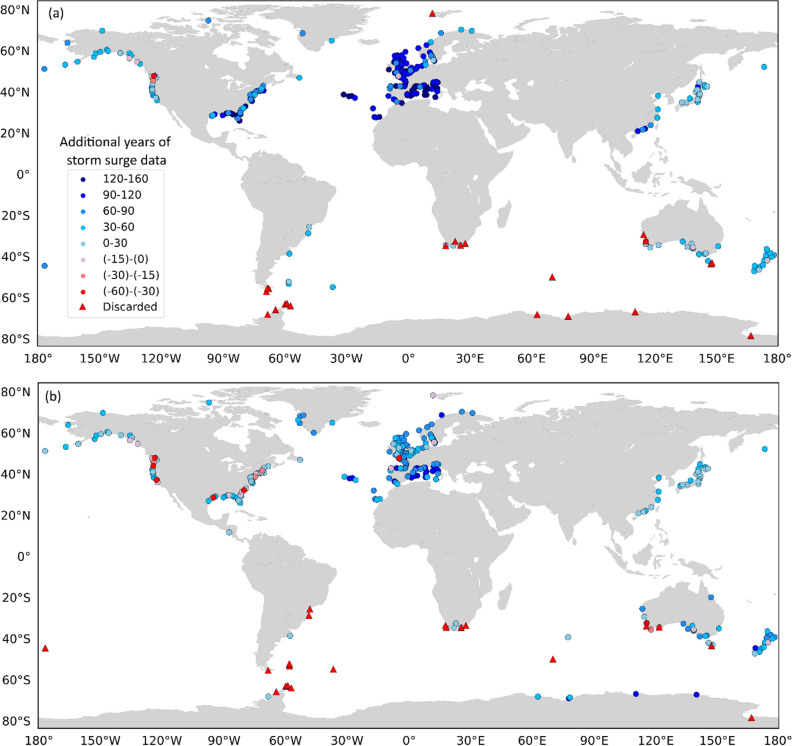
Additional years of reliable storm surge data after change point analysis obtained from G-20CR (**a**) and G-E20C (**b**) compared to the existing observed records. Negative numbers indicate that reliable surge reconstructions are shorter than observations. Red triangles represent tide gauges where surge reconstructions are rejected. Rossum, Guido van, et al., The Python Language Reference, Python Software Foundation; http://docs.python.org/py3k/reference/index.html.

On average, and after removing suspicious data, GSSR^31^ has extended the average storm surge data lengths at the 310 (G-20CR) and 320 (G-E20C) sites from 30 to 111 years (G-20CR) and 16 to 69 years (G-E20C), with significant spatial variability. We find that G-20CR provides at least 100 years of additional surge data (on top of available observed surge information) for 40% of the tide gauges and at least 50 additional years for 68% of the tide gauges; G-E20C provides at least 100 additional years of surge data for 4% of the tide gauges and at least 50 additional years for 46% of the tide gauges (Fig. 4). According to the aggregated results in Table 1, G-20CR leads to the shortest extension of existing data along the US West coast, adding on average 30 years of data. In Europe, on the other hand, an average of 111 additional years of surge data is made available. For instance, at Delfzjil (The Netherlands), G-20CR provides a total of 149 years of reconstructed surge data which is 104 more years in addition to the 45 years of existing observational data (available in the GESLA-2^1^ database).

G-E20C also provides the shortest extension for tide gauges along the US West coast, with an average of 16 additional years of data, and a maximum extension in Europe, with 69 additional years on average (Table 1). There are 9 tide gauges along the US East and Gulf coast, where the observational data is longer than the reconstruction when using G-E20C (Fig. 4b). These are tide gauges with particularly long observational records such as Galveston (102 years) and Atlantic City (94 years), where change points are detected in the reconstructions leading to shorter records compared to observations.

### Trend analysis

#### Long-term trends in storm surge magnitude

**Figure 5:**
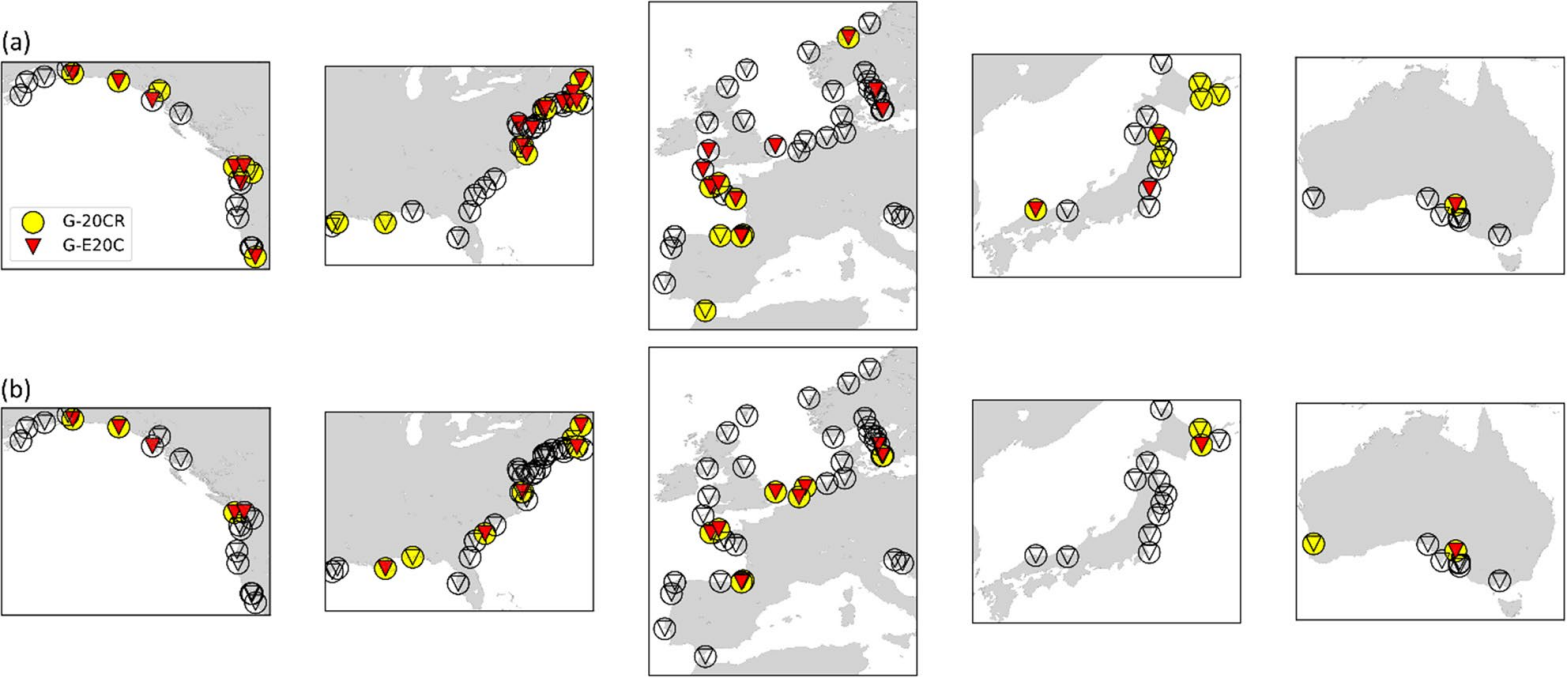
Tide gauges with significant differences in trends between observed surge and reconstructed surge from G-20CR (yellow circles) and G-E20C (red triangles) using the annual 95th (**a**) and 99th (**b**) percentiles. Tide gauges with insignificant differences in trends are shown as transparent circles and triangles. Trends are computed when at least 30 years of overlapping data are available for the 1930–2010 period. Rossum, Guido van, et al., The Python Language Reference, Python Software Foundation; http://docs.python.org/py3k/reference/index.html.

After removing suspicious data based on the change point detection, we calculate and compare trends of the observed and reconstructed surges (see “Methods” for details) to assess their similarities. We use annual values of high percentiles and chose the 95th and 99th percentiles here as those have been used in many previous studies and are often considered as thresholds when performing extreme value analysis. For this comparison, we select 122 tide gauges with at least 30 years of overlapping data between observations, G-20CR, and G-E20C and a minimum of 75% completeness in the observations. For the majority of the 122 tide gauges, no statistically significant differences (5% level) exist between observed trends and reconstruction trends (Fig. 5). Differences between observed surge and G-20CR are found at 25% (95th percentile surges) and 19% (99th percentile surges) of the tide gauges. When comparing observations and G-E20C, significant differences are found at 30% (95th percentile surges) and 18% (99th percentile surges) of the tide gauges. These differences with observations mainly exist along the Salish Sea (US West coast), New England (northeast US coast), and the Atlantic coast of France. For 64% (95th percentile surges) and 78% (99th percentile surges) of the tide gauges, both reconstructions agree with the observed trends, in particular along the US southeast coast, Japan, and the German Bight.

**Figure 6:**
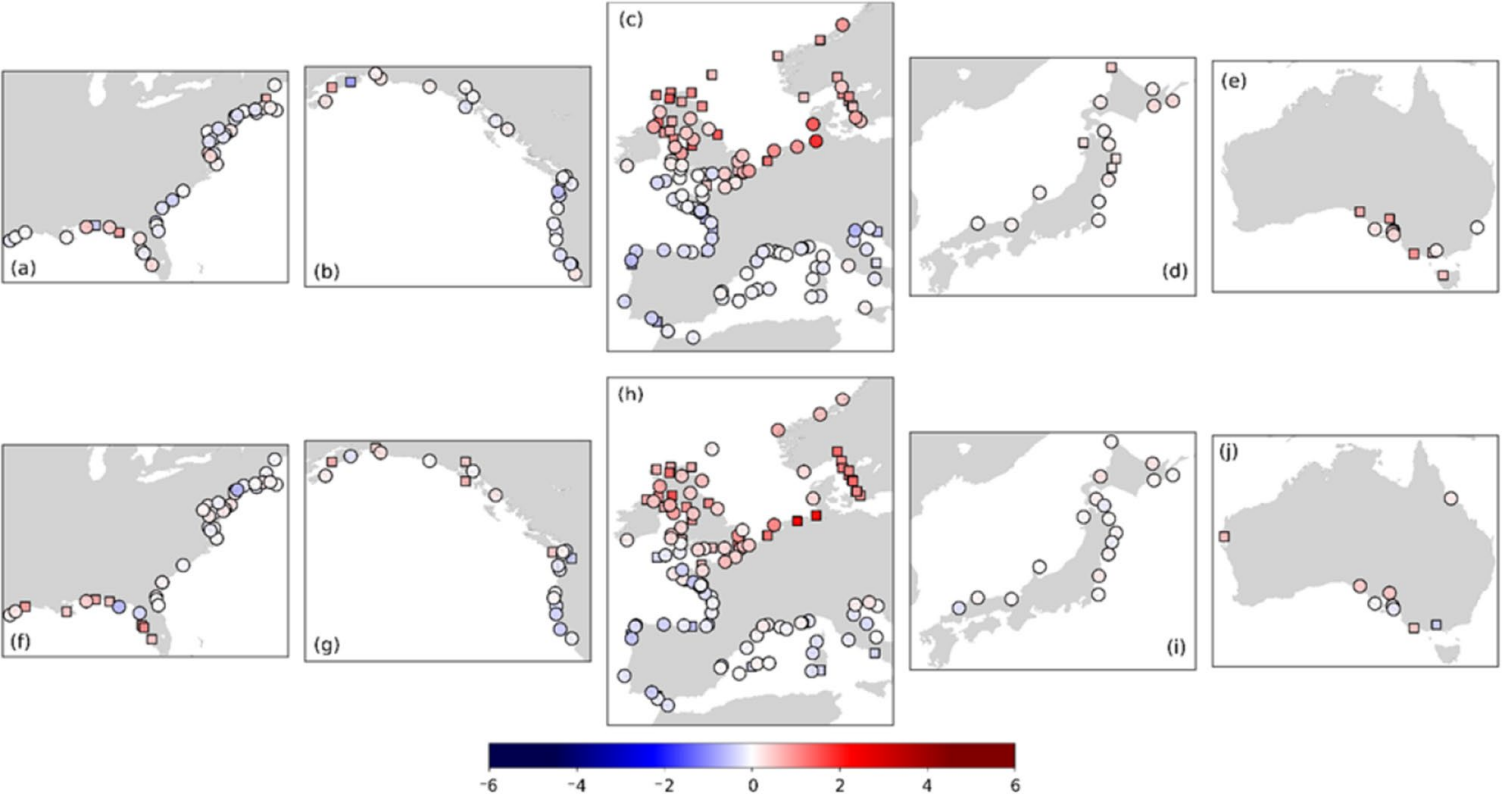
Trends (mm/year) for the annual 99th percentile surge values for G-20CR (**a**–**e**) and G-E20C (**f**–**j**) corresponding to 1950–2015 and 1950–2010 respectively. Rectangle markers indicate significant trends at the 5% significance level. Rossum, Guido van, et al., The Python Language Reference, Python Software Foundation; http://docs.python.org/py3k/reference/index.html.

Next, we investigate G-20CR and G-E20C trends for the 1950–2010 (2015) and 1930–2010 (2015) periods. Those were chosen as a tradeoff between covering relatively long time periods while still having reasonable spatial coverage. Figure 6 shows the long-term trends of the annual 99th percentile surges from G-20CR (a–e) and G-E20C (f–j) for the 1950–2015 and 1950–2010 periods respectively (Supplementary Figs. S1 and S2 show trends for the annual 95th percentile surges). Trends are shown for regions with at least 10 tide gauges. Note that the number of tide gauges can be different in the same region for the two reconstructions, because the change point analysis may have identified suspicious data post-1950 in one reconstruction but not the other.

For G-20CR, significant trends at the 5% significance level are found at 26% of the tide gauges (which were considered originally for change point analysis), notably in the northern UK, Kattegat Bay, southeast Australia, and New Zealand. The largest statistically significant positive trends are derived for the northern UK and New Zealand with magnitudes of 1.9 mm/year and 1.6 mm/year, respectively (Supplementary Fig. S7). Although statistically insignificant, Cuxhaven (Germany) and Esbjerg (Denmark) have the largest trends with magnitudes of 2.48 mm/year and 1.89 mm/year respectively. Small but significant negative trends with an average magnitude of − 0.6 mm/year are derived at 13 tide gauges and those are mostly located along the Atlantic coasts of France and Spain and in the Adriatic Sea.

Similarly, for G-E20C significant trends for the 1950–2010 period are found at 26% of the tide gauges. Positive trends are derived for the US Gulf coast, UK, Kattegat Bay, and the German Bight. The largest statistically significant positive trend of 2.9 mm/year is derived in the southeastern North Sea (for both Cuxhaven in Germany and Delfzjil in the Netherlands), followed by 2.5 mm/year at Nome (Alaska), and 2.1 mm/year at Millport (UK). Very few tide gauges (4%) show negative trends and those are located in the same regions that had negative trends in the G-20CR reconstruction. The largest negative trend is − 1.0 mm/year at Villagarcia (Spain) (Fig. 6).

**Figure 7:**
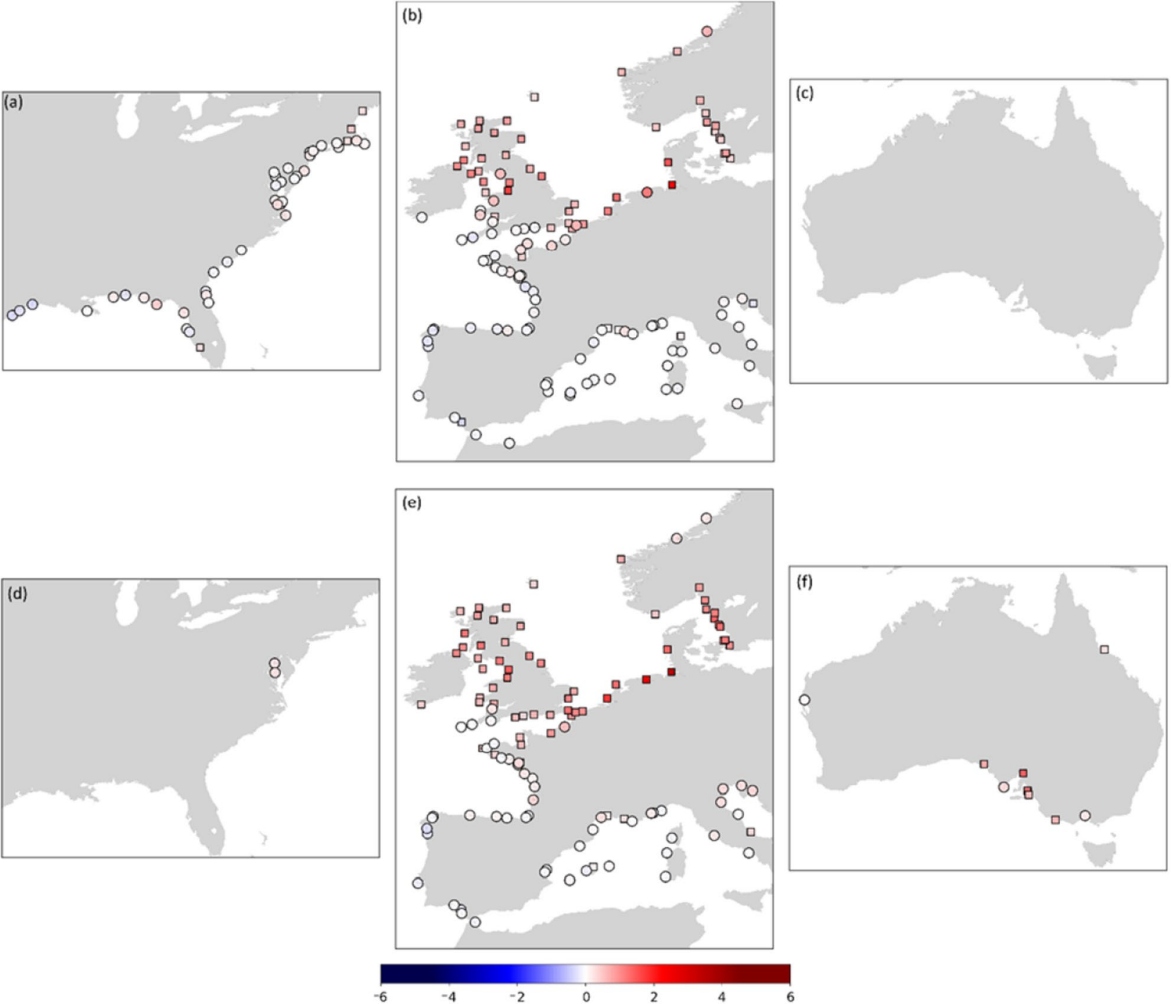
Trends (mm/year) for the 99th percentile surges for G-20CR (**a**–**c**) and G-E20C (**d**–**f**) corresponding to 1930–2015 and 1930–2010 respectively. Rectangle markers indicate significant trends at the 5% significance level. Rossum, Guido van, et al., The Python Language Reference, Python Software Foundation; http://docs.python.org/py3k/reference/index.html.

Over the 1930–2015 (G-20CR) and 1930–2010 (G-E20C) periods, 67% and 85% of the 192(142) tide gauges analyzed show positive trends in the 99th percentile surges (Fig. 7) (see “Methods” on how tide gauges are selected for trend analysis). This is a higher percentage of tide gauges with positive trends compared to the 56% (G-20CR) and 63% (G-E20C) during the 1950–2015 and 1950–2010 periods respectively. Furthermore, many of the same regions—such as the southeastern North Sea and the Kattegat Bay—show persistent positive trends. Tide gauges along the US West coast, Australia (G-20CR), and New Zealand are not included in the analysis for this period since the change point analysis indicated suspicious data before the 1940s (Fig. 2a). Significant positive trends are derived for tide gauges along the US northeast coast (G-20CR), UK, German Bight, Kattegat Bay, and southeast China (G-20CR; results for China are not shown in Fig. 7 because of the small number of tide gauges). The largest statistically significant trends are again derived in the southeastern North Sea with magnitudes of 4.5 mm/year (G-E20C) and 3.0 mm/year (G-20CR) at Cuxhaven (Germany), followed by 3.6 mm/year (G-E20C) at Delfzjil (The Netherlands), 2.3 mm/year (G-20CR) at Gladstone (UK), and 2.0 mm/year (G-20CR) at Esbjerg (Denmark).

#### Trend sensitivity analysis

**Figure 8:**
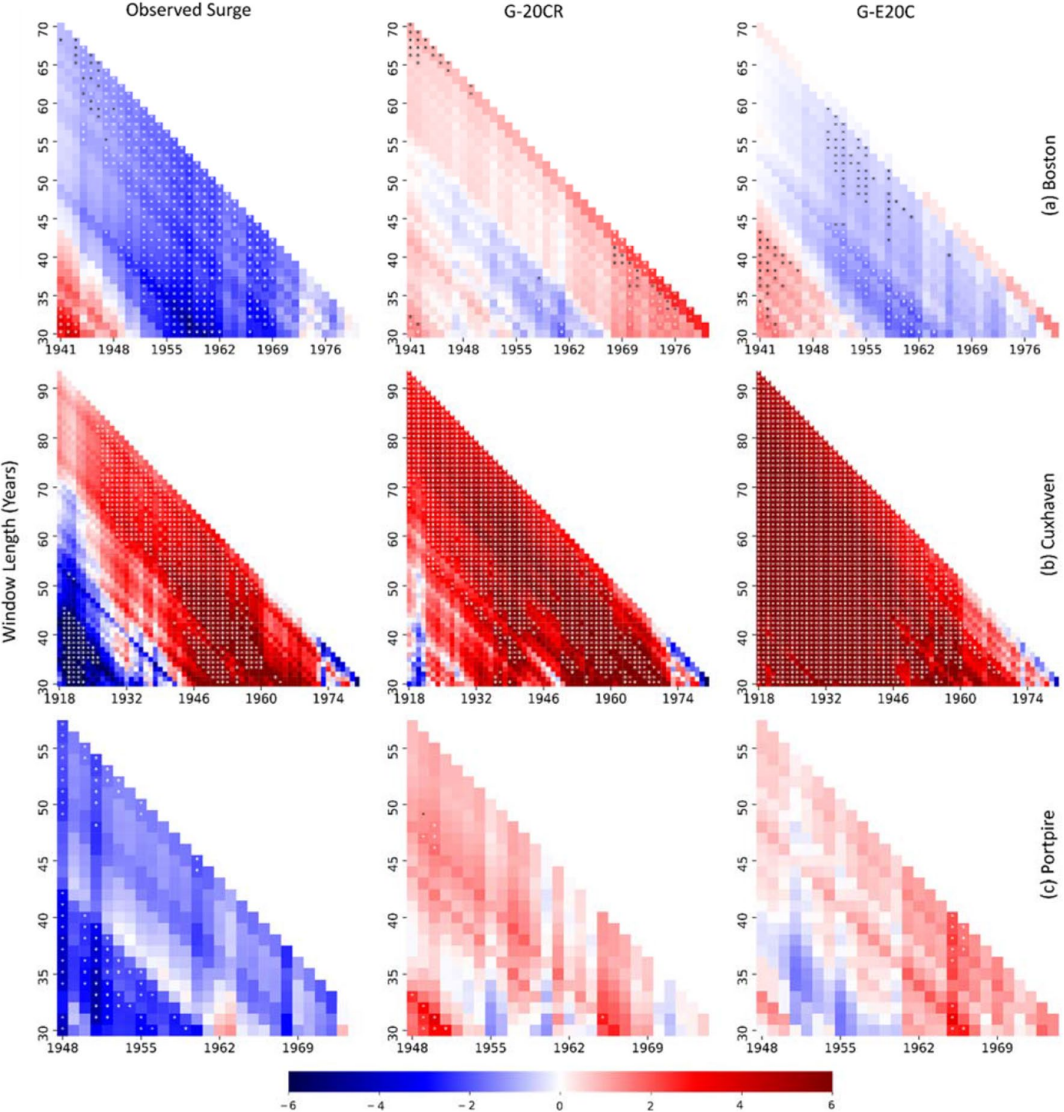
Trend (mm/year) comparison for 99th percentile observed surge (left), G-20CR (middle), and G-E20C (right) for Boston (**a**), Cuxhaven (**b**), and Portpire (**c**). Trends are computed for moving time windows (x-axis) starting with a window length of 30 years, which increases 1 year each step (y-axis) up to the length of available data. Significant trends at 5% significance level are marked with an asterisk.

As discussed in the Introduction, observed surges are usually short and not as continuous as G-20CR and G-E20C. There exist, however, tide gauges with relatively long surge records that can be used to compare against the reconstructed surges. Here we compare 99th percentile observed and reconstructed surges by computing their corresponding trends for various overlapping time windows. We start with a window length of 30 years which is moved by 1 year each time step and repeat the same analysis for longer time windows (adding 1 year each step) (Fig. 8). This allows us to not only compare the reconstructed and observed trends for many more time periods than were used in the previous section, but also shows how multidecadal variability affects observed and reconstructed trends. In Cuxhaven (Fig. 8b), for example, negative trends are found in observations early in the record when using shorter window lengths; and while G-20CR also shows some negative trends early in the record and for short window lengths, the overall patterns in both reconstructions are different, with more persistent positive trends compared to observations. At Port Pire (Fig. 8c), also both G-20CR and G-E20C show positive trends for most time periods and window lengths, while observed trends are mostly negative. In Boston (Fig. 8a), G-E20C agrees well with observations in terms of the sign of the trends, while G-20CR shows very different patterns. More examples are provided in Supplementary Fig. S5 with similar conclusions, i.e. agreement between reconstructions but not with observations in Astoria, relatively good agreement between G-E20C and observations in Brest, and general agreement between all three in Fremantle for most time periods and window lengths. Overall, there is more agreement when trends are derived for longer time windows. Check Supplementary Tables 1–4 for detailed results of the trend analysis.

#### Comparison of trends for the satellite era from all GSSR reconstructions

Finally, we compare trends in storm surge magnitudes of all five reconstructions available in GSSR with each other and with observations for the overlapping period from 1980 to 2010, for which many more tide gauges provide (near-)continuous records. We also include an ensemble mean (G-EnsMean) based on all GSSR reconstructions. Satellite data is assimilated into all reanalysis products over that time period, and spurious long-term trends due to incosnistancies in the assimilated data are less likely to occur. However, over a 30-year period decadal variability can have significant effects on trends and those long-term variations may be represented differently in the reanalysis products and associated GSSR reconstructions (as demonstrated for G-20CR and G-E20C in the previous section for selected locations).

**Figure 9:**
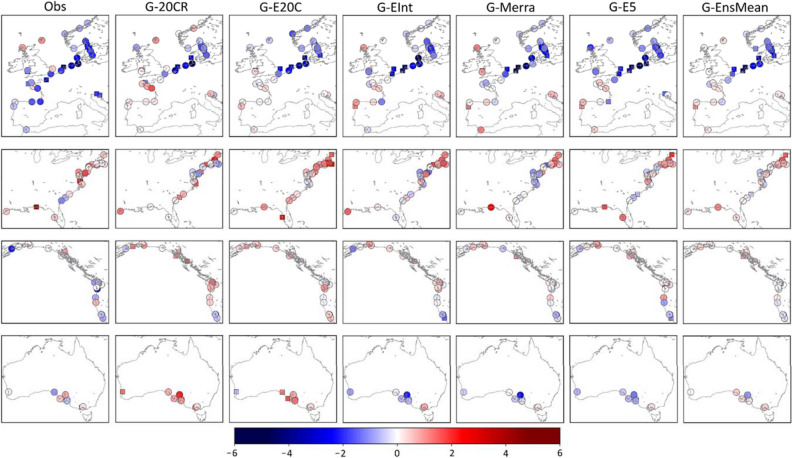
Trends (mm/year) for the 1980–2010 period for all seven datasets (including ensemble mean); significance is assessed at 5% level and significant trends are shown as rectangles. Rossum, Guido van, et al., The Python Language Reference, Python Software Foundation; http://docs.python.org/py3k/reference/index.html.

**Figure 10:**
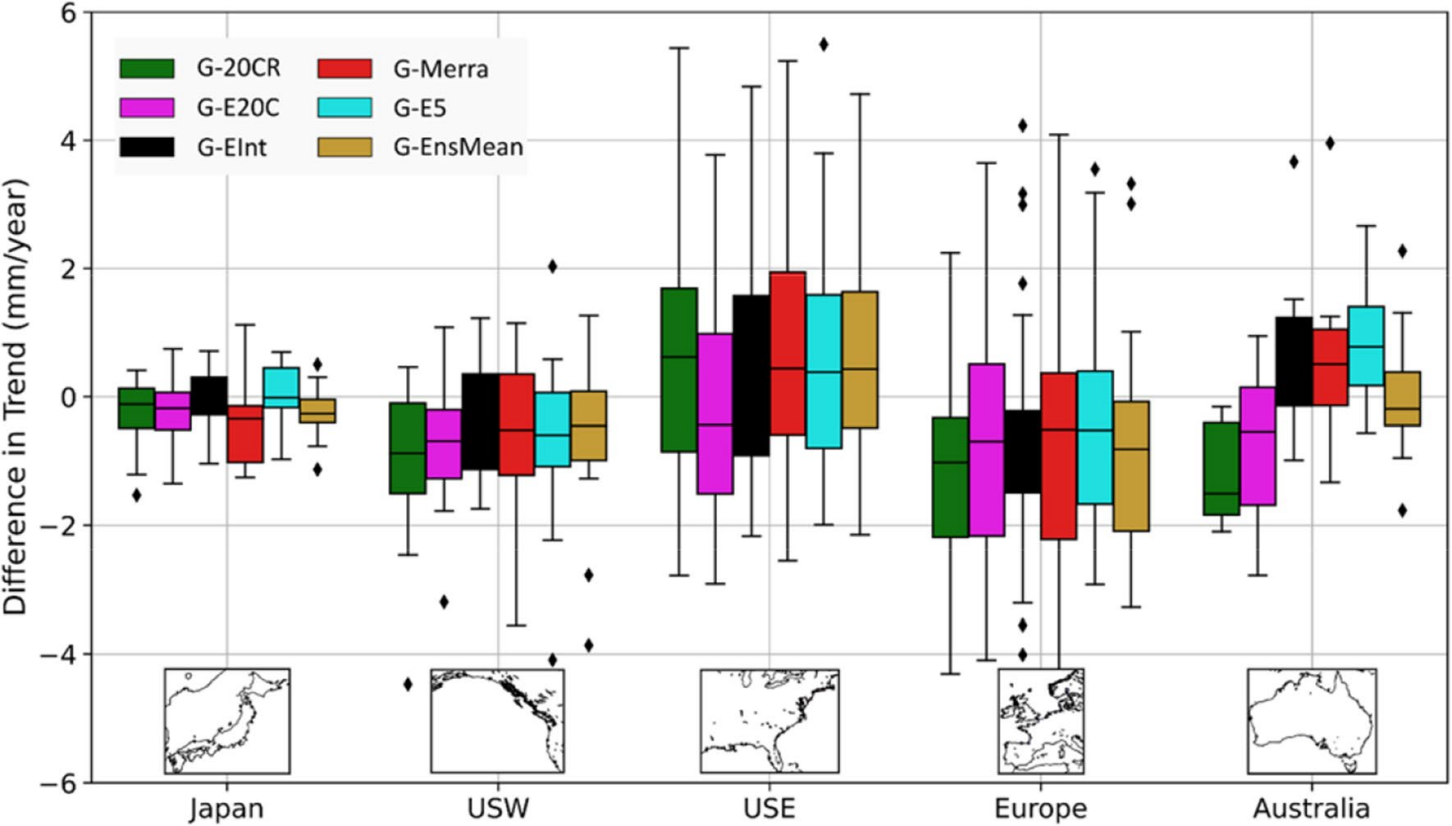
Comprison of GSSR trends with observed trends for six reconstructions (including ensemble mean) and five regions. GSSR trends are subtracted from observed trends, including their signs. Boxes indicate the interquartile range (IQR) (difference between 75 and 25th percentiles), upper and lower marks represent 75th percentile + 1.5 * IQR and 25th percentile – 1.5 * IQR respectively, and diamonds are considered outliers.

Trend analysis for the satelite era shows generally good agreement for Europe in terms of the spatial distribution of observed trends and GSSR trends as well as amongst the different GSSR trends themselves (Fig. 9). All seven datasets (including G-EnsMean) show strong negative trends along the southeastern North Sea and the Kattegat Bay, the largest negative trend being − 6.9 mm/year at Cuxhaven (G-20CR). The actual magnitude of GSSR trends, however, is smaller than that of observed trends (Fig. 10) for most of the tide gauges in Europe. Tide gauges along the Atlantic Coast of France, Spain and North Adriatic Sea have larger negative observed trends which is not reflected in most GSSR reconstructions. Moreover, tide gauges along the Bay of Brest (Brest, Le Conquet) and Loire Estuary (Saint Gildas) show stark differences between observed and GSSR trends. Observed trends at all three tide gauges are negative (− 2.58 mm/year at Brest, statistically significant), whereas GSSR trends are mostly positive, except for G-EInt and G-Merra. Along the US East coast, in the New England area all seven datasets indicate a positive trend for the majority of tide gauges. In the Chesapeake Bay region, there are differences between GSSR trends and observed trends. All GSSR reconstructions (except G-E20C) show negative trends in this region, whereas observed trends are all positive. The largest observed trend has a magnitude of 3.4 mm/year (statistically significant) at Chesapeake Bay Bridge Tunnel. Refer to Supplementary Table 5 for detailed results of this analysis.

Along the US Gulf coast there is a positive trend in most datasets (except G-20CR and G-EInt). The observed trend at Pensacola is 5.2 mm/year (statistically significant), which is the largest positive trend observed in all tide gauges considered in this study during the 1980–2010 period. In general, observed trends are positive and larger in magnitude than GSSR trends in this region which leads to the largest differences between GSSR and observed trends as shown in Fig. 10.

On the US west coast, differences exist in trends between observed surges and GSSR reconstructions mostly for tide gauges on the Columbia River and Salish Sea. While observed trends are negative at Astoria, (− 4.1 mm/year, statistically insignificant) some GSSR trends are positive (G-20CR, G-E20C, and G-EInt) and others negative but very small in magnitude (G-Merra, G-E5, and G-EnsMean). In the southwest (Alameda, Monterey, and San Francisco), there is a general agreement (statistically insignificant but negative trends) among most datasets (except G-E20C).

In Australia, G-20CR and G-E20C generally show positive trends (see also Fig. 10) which is not the case for the satellite era reconstrutions (G-EInt, G-Merra, and G-E5). Differences are most pronounced at Portpire where observed surge, G-20CR, and G-E20C show statistically insignificant but positive trends and the other three GSSR reconstructions show negative trends. The ensemble mean reconstruction (G-EnsMean) gives the smallest difference compared to observed trends (Fig. 10).

#### Trends in storm surge frequency

To study the spatial patterns in frequency trends of extreme surges, we cluster tide gauges into eight regions: US east coast, US west coast, US Gulf coast, east Asia (tide gauges from Japan and China), Oceania (tide gauges from Australia and New Zealand), Mediterranean, western Europe, and the Kattegat Bay (tide gauges from Sweden and Norway). Here we investigate the trends in storm surge frequency for both centennial reconstructions (G-20CR and G-E20C) for the 1930–2010 (2015) and 1950–2010 (2015) periods, after suspicious data identified from the change point analysis is removed. To quantify storm surge frequency at individual locations, the 95th percentile of the entire reconstructed surge time series is considered as a threshold. The number of annual storm surge events exceeding this threshold is derived at each tide gauge and the resulting time series are averaged per region and a linear trend is estimated for the regional average annual storm surge frequency.

Differences between trends in annual exceedances (after declustering, see “Methods”) above the 95th percentile surges for observed surges and reconstructed surges (G-20CR and G-E20C) are computed for 133 tide gauges that have storm surge data available during the 1930–2010 (2015) period (detailed results not shown). Results show that the trends for the number of annual exceedances above the 95th percentile of the observed and reconstructed surges for the overlapping periods are not statistically different (at 5% significance level) for 73% and 81% at the tide gauges for G-20CR and G-E20C respectively.

**Figure 11:**
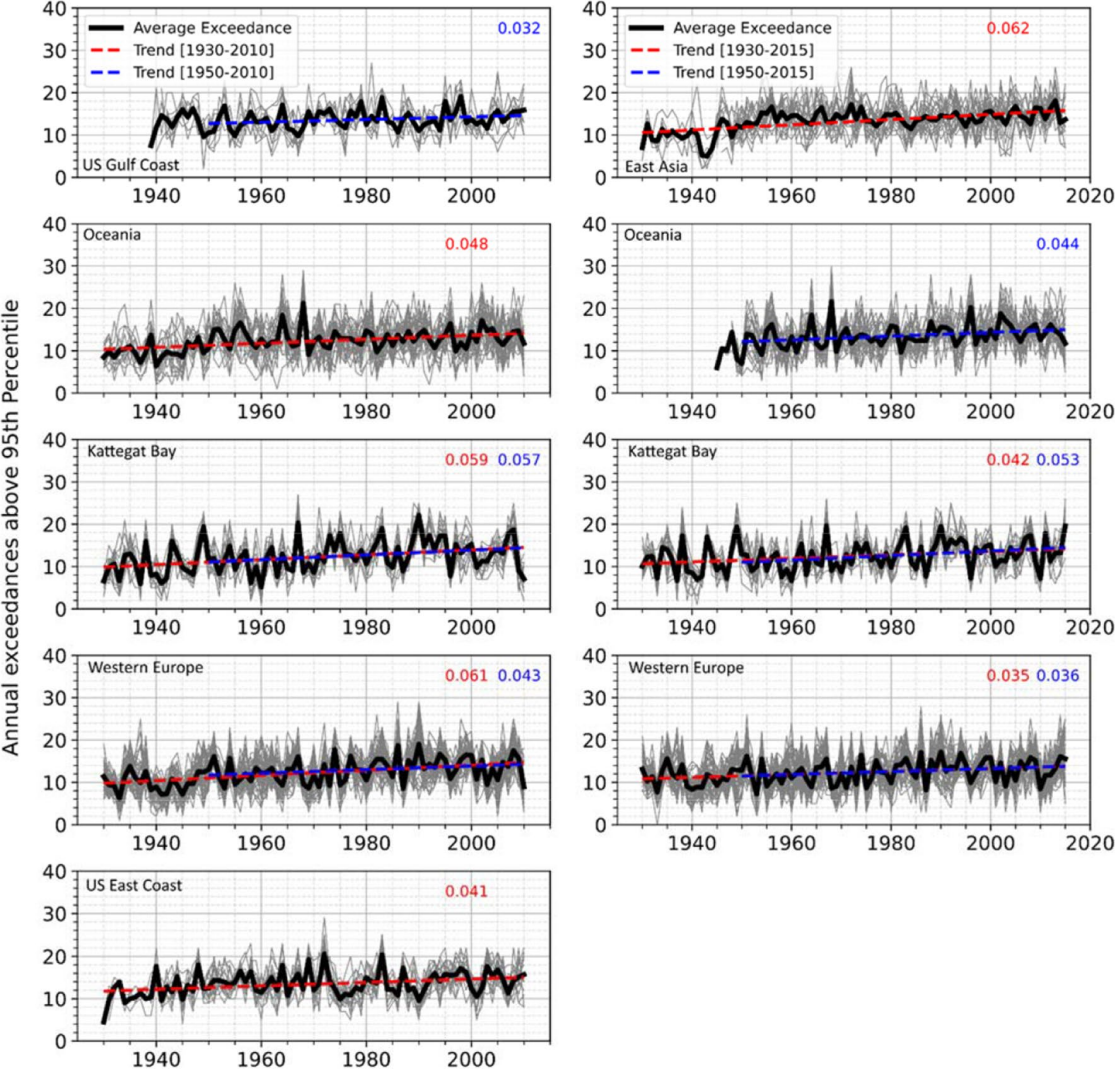
Regional storm frequency trends. Linear trends are fitted to the average number of annual exceedances above the 95th percentile (bold black line) for G-E20C reconstructions (left) and G-20CR reconstructions (right). Gray lines indicate the number of surge events exceeding the 95th percentile threshold for individual tide gauges in the given region. Only regions with at least one significant trend (dashed lines) are shown.

**Figure 12:**
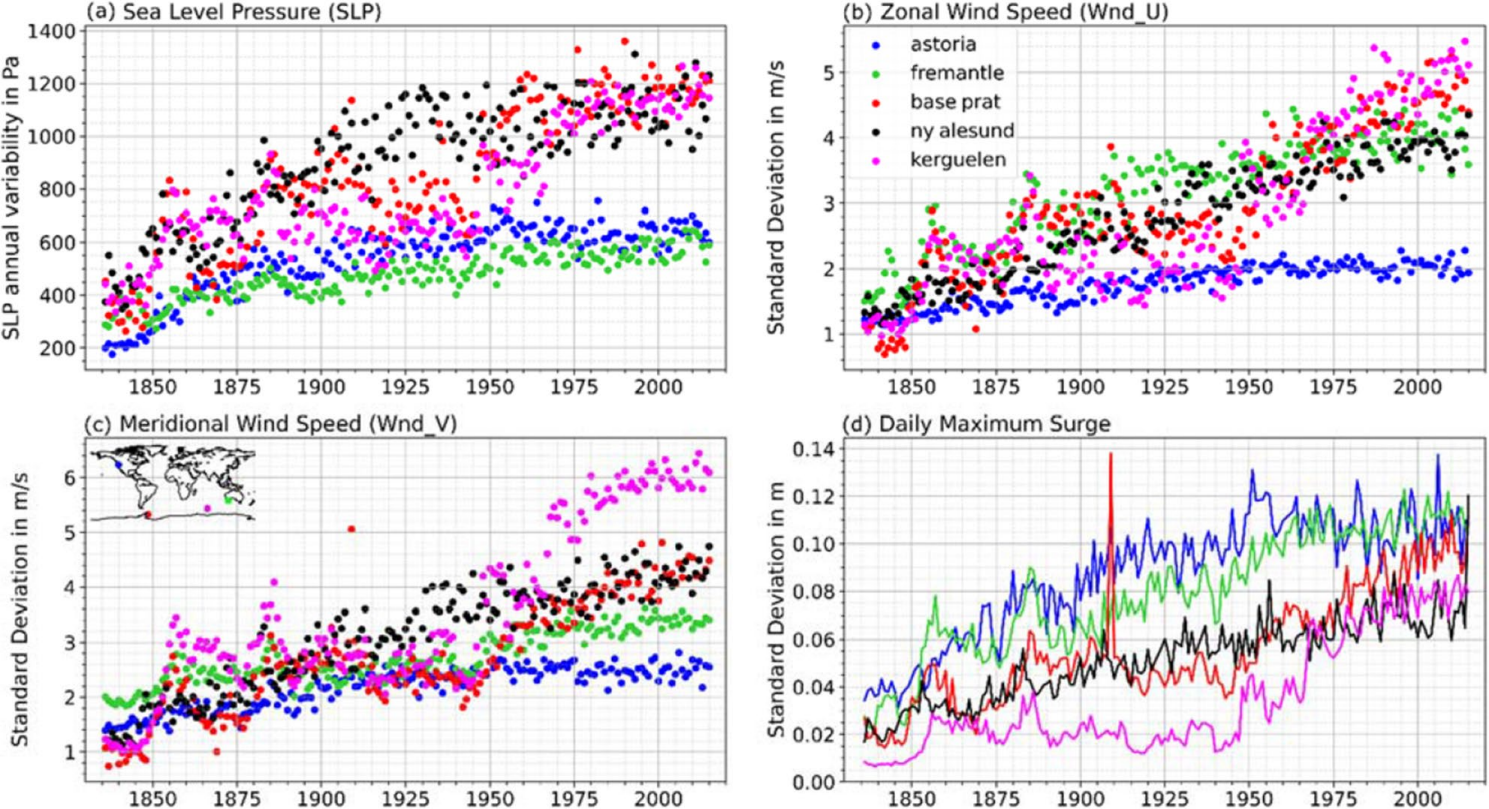
Decreasing variability (expressed as annual standard deviation) for 20CR predictors mean sea-level pressure (**a**), zonal wind speed (**b**), meridional wind speed (**c**), and G-20CR (**d**) for selected tide gauges.

Figure 11 shows the trends for six regions, as the other two regions (Mediterranean and US west coast), do not show significant trends at the 5% significance level for either of the reconstructions. East Asia does not show significant trends in G-E20C, whereas no significant trends exist for the US Gulf and east coasts in G-20R (and hence panels are not shown in Fig. 12). Similar to the trend analysis for the surge magnitudes, the frequency trends are computed for two time periods, 1950–2010 (2015) and 1930–2010 (2015). The gray lines in Fig. 11 represent the number of storm surge events exceeding the 95th percentile threshold for individual tide gauges in the given region, whereas the bold black line represents the average number of exceedances from which the two trends are derived. Results show positive trends for all six regions and the two selected time periods (shown by different colors; trend lines are only shown for significant trends). Overall, the largest trends are found across the Kattegat Bay and western Europe for both G-20CR and G-E20C.

## Discussion

### Change point analysis

We apply a Bayesian change point analysis on G-20CR and G-E20C storm surge reconstructions as well as the predictors that were used to derive them. The goal is to identify and remove suspicious data, related to inconsistencies in the reanalysis products, from the surge reconstructions. Figure 12 shows the annual variability time series for 20CR predictors and the associated reconstruction G-20CR for five tide gauges in the Arctic, Antarctica, Australia, New Zealand, and the US northwest coast. In all cases, sharp decreases in the variability of the reanalysis predictors and reconstructed surges exist when going back in time. G-E20C (not shown here) also shows such a decrease in annual variability in several but not all of these tide gauges. Some of the tide gauges like Base Prat and Kerguelen show a very rapid decline in the variability leading to a change point year in the 2000s. Therefore, surge reconstructions for these tide gauges and others with similar suspicious behavior are not considered for trend analysis. They are marked as red triangles in Figs. 3 and 4. For most of the other tide gauges in these regions, our change point analysis shows that G-20CR should be considered only from the mid-twentieth century onward since change points are detected in the 1940s and 1950s (see Supplementary Figs. S3 and S4). This aligns with the findings from Brönniman et al.^32^ who showed the strong downward trend in 20CR wind speeds in the Arctic, northeastern Canada, and the northern North Pacific before 1940. This is due to the scarcity of observations in these regions used in the data assimilation for the 20CR reanalysis. ERA-20C predictors and G-20C, on the other hand, do not show such rapid decline in variability (see Supplementary Figs. S3 and S4 for examples). A possible explanation for this might be the assimilation of surface marine wind observations into ERA-20C which is not the case for 20CR^18,26,27^.

The comparison presented in this section is not indicative of the superiority of one surge reconstruction (or reanalysis) over the other and should not be interpreted as such. For the majority of the tide gauges used in this study, the record lengths of the observed surges are too short to robustly compare trends in the annual variability with that of the reconstructed surges. However, the two centennial reconstructions, together with the other GSSR reconstructions (depending on the time period of interest) can be considered as an ensemble (Fig. 10) when used, for example, in coastal flood risk assessments to better understand the inherent uncertainties.

### Trend analysis

Using the long storm surge reconstructions from GSSR, we investigate how the magnitude and frequency of extreme surges changed over the last ~ 90 years. One of our key findings is that both storm surge reconstructions, G-20CR and G-E20C, indicate a consistent positive trend for the 1930–2010 (15) and 1950–2010 (15) periods for extreme surges (annual 99th and 95th percentiles) in northern UK, the southern North Sea, and the Kattegat Bay. Similar positive trends were reported by Donat et al.^25^ from analyzing storminess from the 20CR reanalysis in the North Sea and Baltic Sea regions. Over the 1950–2008 period, Brönniman et al.^33^ also found positive trends in strong and extreme wind speeds in northwestern Europe when using 20CR. Dangendorf et al.^30^ concluded that 20CRv2 provides a useful database for the same region for the time period after 1910 because reconstructed storm surges for the tide gauge Cuxhaven showed similar variability and trends compared to observed storm surges over that period. In our analysis G-20CR and G-E20C show positive trends at Cuxhaven since 1910 (2.6 mm/year and 4.8 mm/year for G-20CR and G-E20C, respectively).

The positive trends we find in GSSR reconstructions for northern UK, the southern North Sea, and the Kattegat Bay during the 1950–2015 period can be explained, in part, with long-term variability in the North Atlantic Oscillation (NAO) during the 1950–1990 period^34^. They are also consistent with an eastward shift of the NAO’s centers of action that occurred over the same period. NAO is one of the large-scale circulations that determine the storminess in the North Sea region. However, the 1930–2010 (2015) trends derived from the surge reconstructions are in contrast to the insignificant trends reported for observed surges in northwestern Europe^30,35,36^, albeit with considerable interannual and multidecadal variability. Focusing on the period from 1970 onwards, Menéndez et al.^37^ found no significant trends in storm surge magnitude in the European Atlantic coast. For the same period, we also find insignificant trends in G-20CR and G-E20C (except for a few tide gauges in the northern UK).

Next, we showed that during the common period 1980–2010, where all GSSR reconstructions overlap and many more tide gauge provide (near-)complete data, spatial distribution of trends is similar across all datasets (including an ensemble mean of the GSSR reconstructions) in many regions. This is particularly pertinent to tide gauges in northern Europe and northeast coast of the US. There are, however, regions where trends differ (in magnitude and sometimes also in sign), particularly in estuaries and bays. For example, at tide gauges along the Chesapeake Bay, Columbia River, Salish Sea, Bay of Brest, and Loire Estuary, observed and GSSR trends (for the majority of reconstructions) have opposite signs. This could be due to the modulating effect of river discharge on water levels in bays and estuaries^38,39^ not captured by GSSR.

We also show that GSSR centennial reconstructions exhibit statistically significant positive trends in storm surge frequency during the 1930–2010 (2015) and 1950–2010 (2015) periods. The tide gauges with the largest positive trends in surge magnitude (95th and 99th percentile) also often have the highest positive trends in the storm frequency (e.g., northwestern Europe and the Kattegat Bay). This aligns with previous studies that report an increase in the storm frequency for the high-latitude North Atlantic and northern Europe^26^, including the North Sea^25^. On the other hand, Krueger et al.^27^ argued that the long-term positive trend of the storm index, estimated from the 20CR reanalysis in northern Europe and northeast Atlantic, is implausible as the same storm index for the upper percentiles of the observed geostrophic wind speeds does not show a similar long-term trend. However, the storm indices from the 20CR reanalysis and the observed geostrophic winds behave similarly in the second half of the twentieth century.

As noted above, a limiting factor in our analysis is the potential impact of reanalysis inconsistencies on the reconstructed surges that might introduce spurious long-term trends in some regions. The Bayesian change point detection method successfully identified suspicious changes in the variability of surges and predictors at tide gauges along the northwestern coast of the US, northern Australia and some high-latitude regions. These changes in the variability, if not accounted for, would lead to significant and implausible trends in high-percentile surge time series (such as the annual 95th and 99th percentiles used here). While the change point analysis identified instances where that was the case, the methodology might still miss small and subtle trends that can be attributed to inconsistencies arising from the atmospheric reanalyses.

The case of Astoria (Fig. 2) for instance, shows some of the challenges related to the change point analysis and comparison to in-situ observations. The year 1948 (47) is identified as a change point for G-20CR (G-E20C), based on the change point probabilities as well as the visually obvious shift in the four variables during the 1940s. This could be associated with the sparse amount of observations assimilated into the reanalysis products during and shortly after World War II^40^. On the other hand, the specific years (1947 and 1948) where the change points are detected, may also be associated with a shift from the warm to the cold phase of the Pacific Decadal Oscillation^41^. In the Pacific Northwest, the cold phase of the PDO is associated with cooler water temperatures and changes in streamflow patterns (due to the change in temperature differences between cold and warm PDO phases)^42^, both of which can influence water levels^43,44^. Moreover, the year 1948 was particularly stormy, leading to a large snowpack and the second largest flood on the Columbia River since records began^45^. Hence, the particular attribution of a change point to the year 1947–1948 may be related in part to natural variability. We note, however, that the shift to warm PDO phases in ~ 1925 and ~ 1977 are not picked up by the change point analysis. Hence, we conclude that inconsistencies in the reanalysis lead to a drop in the variability in the 1940s, with the exact year(s) possibly conflated by background atmospheric/oceanic variability.

To conclude, our trend analysis on GSSR storm surge reconstructions demonstrates that in northwestern Europe, both G-20CR and G-E20C show consistent positive trends for the periods starting in 1930 and 1950 in magnitude as well as frequency, particularly in the southeastern German Bight. From the satellite-era trend analysis (1980 to 2010) we find consistently negative trends for major parts of northern Europe, which also agree with the observational data. Results are also relatively consistent across reanalysis products in the northeastern US, whereas in other regions we find pronounced differences in trends when comparing between reanalysis products, for example southern Australia and in bays and estuaries. The ensemble mean often resembles the spatial patterns of trends in the observed data best and is hence the preferred approach, but it limits the analysis to a relatively short period where all reanalysis products provide data. Trends derived from centennial reanalysis products over longer time periods provide useful insights as to where changes in storm surges may have taken place in the past (and could continue in the future), while at the same time being cautious in the interpretation due to the inhomogeneities in reanalysis products. In addition to performing trend analyses presented here, the underlying GSSR data is also useful for other applications, such as studying intra-annual to multi-decadal variability. In the future we plan to apply bias correction to the GSSR reconstructions and use those for extreme value analysis and to study spatial storm surge footprints^46^, among others.

## Methods

### Data

We use daily maximum surge reconstructions obtained from the Global Storm Surge Reconstructions database (GSSR, http://gssr.info) developed in Tadesse and Wahl^31^. GSSR comprises two centennial and three satellite-era storm surge reconstructions, all of which have been obtained with data-driven models from Tadesse et al.^47^ using wind speed and mean sea-level pressure forcing from five different atmospheric reanalysis products. GSSR reconstructions are available for 882 globally distributed tide gauges, and they have been validated against in-situ daily maximum surge observations from tide gauges^47^. Observed storm surges are extracted from sea-level measurements from the GESLA-2 database^1^ as the difference between the measured water level and the tidal prediction, after removing the annual mean sea level. Hourly water level records for Astoria (1855–1876) shown on Fig. 2d were obtained from Talke et al.^44^.  We only select GSSR reconstructions corresponding to tide gauges that show correlations with observed daily maximum surges of 0.7 or greater. This results in 310 and 320 tide gauges with G-20CR and G-E20C reconstructions, respectively.

### Change point analysis

Reanalysis products are sensitive to the assimilated meteorological and/or oceanic observations (changing over time), which may result in spurious trends in key outputs variables^27,48,49^. Furthermore, due to sparsity in assimilated observations, atmospheric events, particularly small-scale events (hurricanes, atmospheric rivers), may be poorly represented, which may result in an underestimation of modelled variables such as peak wind speeds or minimum pressure^50,51^. Systematic underestimations would therefore become visible in the variability of output variables from the atmospheric reanalysis products and therefore also translate into underestimated variability in the GSSR reconstructions (see Fig. 1). We therefore hypothesize that time-periods with a persistent decrease in the variance (or standard deviation) of surges in GSSR and/or its forcing variables likely indicate systematic model drifts rather than real trends.

In order to identify suspicious data in GSSR we apply a Bayesian change point analysis to annual standard deviation time series of GSSR surges and the atmospheric forcing datasets from 20CR and ERA-20C. The Bayesian change point analysis is carried out using the R package “bcp” version 4.0.3^52^ in RStudio version 1.1.453. The package implements a Markov Chain Monte Carlo (MCMC) approximation of the Bayesian change point analysis methodology presented in Wang et al.^52^. It is based on the product partition model^53,54^ that separates a time series into several partitions based on different parameters (for instance, the mean and variability of the time series). The product partition model considers the number of change points and their positions as random variables and assumes that there exists an unknown partition ρ of the set {1, 2,…, n} that divides the time series into b contiguous blocks (random variable ranging from 1 to n, where n is the length of the time series). We used 500 MCMC iterations for our analysis. At the end of each iteration, the posterior distribution for the random partition, the number of change points, and change point probability of a given year are updated. For each year, we average change point probabilities corresponding to the four variables (the reconstructed surge and the three predictors). This is done to find the years in the time series where all (or most) of the variables show unusual changes in the variability. Sometimes, one or more variables show a deviation from the "typical” values, but this could be an artifact and may not be reflected in other variables. From the estimated average change point probabilities, we identify years in the time series where change point probabilities are equal or greater than a set of cutoff probabilities. In our analysis, cutoff probabilities of 15%, 20%, 25%, and 30%, are used to find change point years. Usually there are multiple years in the annual variability time series where a given cutoff probability is exceeded. In that case, we select the most recent year as the change point for the given cutoff probability. Only surge data from change point years onward are considered to quantify the trends in magnitude and frequency of daily maximum surges.

As mentioned in the introduction, using the RMSE time series between observed and reconstructed surges would be the preferred approach to identify spurious trends in reconstructions. Although this is not feasible globally due to lack of data, there are a few tide gauges (Supplementary Fig. S6) with long records for which the annual RMSE between daily maximum observed and reconstructed surges time series was used to implement change point analysis (in addition to the annual standard deviation time series). There are noticeable differences in change point analysis results when using annual RMSE vs annual standard deviation time series of the reconstruction alone (Supplementary Fig. S6). This could be due to unrealistic surge values in observations (e.g., due to time shifts in the tidal analysis or other data issues) or in the GSSR reconstructions that can lead to very high RMSE values for individual years which in turn would be wrongly flagged as change points. For instance, in Brest (France) there is a change point identified in 1975 with high probability (97%) when the annual RMSE time series is used. However, there is no persistent deviation of the annual RMSE time series before or after this period. The change point analysis does not detect any change point for the same period when the annual standard deviation time series is used. Similar issues are found in Seattle (Supplementary Fig. S6d) when using the annual RMSE time series for change point detection (particularly 1960 onward).

As an alternative to the annual standard deviation time series, we tested using the annual interquartile range. This is a measure of variability that is more suitable for skewed distributions and is robust against outliers. The interquartile range is computed by taking the difference of the 75th percentile and 25th percentile values of the system variable (daily maximum observed and reconstructed surges) for a given year. Using the annual interquartile range time series for change point analysis showed very similar results to that of annual standard deviation. Hence, in this study we focus on the annual standard deviation time series of predictors and surge reconstructions to detect change points.

In addition to identifying the change point years corresponding to the different cutoff probabilities, a visual inspection of the individual annual variability time series is carried out. This is done to avoid instances where extreme events (such as surges caused by hurricanes) not adequately represented in either the observations or the reconstructions are identified as change points, or when subtle but consistent changes in the variability time series are not picked up by the change point algorithm (i.e., change point probabilities are below the cutoff values we considered). Furthermore, we assess if similar shifts occur in the different predictors and the surge reconstruction. In other words, if a change point year indicates only a change in one variable but no significant change is reflected in other variables, this change point year is disregarded. Hence, while the automated change point analysis provides initial indication of when change points occurred, the results are manually corrected in some instances for the various reasons outlined here.

### Trend analysis

First, trends in extreme storm surges are calculated for the two centennial GSSR reconstructions, after suspicious data was removed, and we focus on the periods 1930 to 2010 (2015) and 1950 to 2010 (2015). Trends are computed by fitting a linear regression model to the annual 95th and 99th percentile surges from G-20CR, G-E20C, and observations where available. The standard errors of the linear regression coefficients representing the trends are adjusted for heteroscedasticity and autocorrelation using the Newey-West estimator^55^. Before fitting trends to extreme surges from the reconstructions, we compare the trends in extreme surges from observations to trends in extreme surges from G-20CR and G-E20C. We limit our analysis to tide gauges with > 30 years of data and > 75% completeness between the years 1930 and 2010. Trends are computed using the common period between observations and reconstructions at each tide gauge. We check if the trends from observations are significantly different from the reconstruction trends at the 5% significance level. Our null hypothesis is that there is no significant difference between the trends obtained from observations and reconstructions for their period of overlap. For the annual 95th and 99th surge time series, a categorical variable is added to differentiate the time series as observation, G-20CR, or G-E20C. An interaction term (product of the categorical variable and the years) is then added as an additional predictor to fit linear trends to the annual 95th and 99th surges. We calculate the p-values for the coefficient of the interaction term and determine its significance at the 5% significance level. If the p-value for the coefficient of the interaction term is higher than 0.05 then the null hypothesis cannot be rejected. In other words, there is no significant difference between the trends in observed surges and reconstructed surges.

Following this test, we estimate trends in G-20CR and G-E20C (above 95th and 99th percentiles) for the 1950–2010 (G-E20C)/1950–2015 (G-20CR) and 1930–2010 (G-E20C)/1930–2015 (G-20CR) periods. As start years for the reconstructions vary among tide gauges due to the change point analysis, we constrain G-20CR and G-E20C strictly to the chosen time period before computing trends. For instance, when computing trends for the 1950–2015 period, we select only tide gauges that have data covering the entirety of this period.

To investigate the sensitivity of trends to start dates and periods covered and to compare and contrast trends from observation, G-20CR, and G-E20C (where long enough observations exist), a trend sensitivity analysis is carried out (Fig. 8 and Supplementary Fig. S5). A window of 30 years is selected as the starting window length where trends are computed, and the window is shifted one year each time step. Trends are then computed for each (moving) window length (by increasing the window length by one year until record length is reached). Availability of 75% of the data is required for each window. For windows where this is not met, trends are not computed (see for example Supplementary Fig. S5b). When gaps exist in observations they are also introduced to G-20CR and G-E20C, so that the trend comparison considers exactly the same period. We also compare observed trends with trends from all five GSSR reconstructions, including an ensemble mean (G-20CR, G-E20C, G-EInt, G-Merra, and G-E5, and G-EnsMean) for the 1980–2010 period where all datasets overlap (Figs. 9, 10) and (near-)complete observations are available for many tide gauges. Results are aggregated for five regions (Europe, US east Coast, US west Coast, Japan, and Australia) (Fig. 11).

Finally, we compute trends in annual storm surge frequency for G-20CR and G-E20C during the 1930–2010 (2015) and 1950–2010 (2015) periods. Trends are derived for the number of annual exceedances over the 95th percentile threshold (calculated from the reconstructions over the 1930–2010 (2015) period(s)). We use a 3-day window to decluster daily maximum surges that are above the 95th percentile threshold. We group tide gauges into eight different regions across the globe and derive the regional time series of annual number of extreme surges (> 95th percentile) by averaging them over the tide gauges within each region before fitting a linear regression model and adjusting for heteroscedasticity and autocorrelation. We also compare frequency trends from reconstructions and observations and test whether they are significantly (5% level) different from each other using the same approach as outlined above for comparing observed and reconstructed trend magnitudes.

## Supplementary Information

Supplementary Information.

## Data Availability

The Global Storm Surge Reconstructions (GSSR)^31^ (http://gssr.info) is a publicly available database that contains five daily maximum storm surge reconstruction datasets derived by forcing five climate reanalyses into a data-driven storm surge model^47^. The surge reconstructions obtained after incorporating the change point analysis as well as all change point analysis plots can be accessed at (http://gssr.info/changepoint). Trend analysis results for all tide gauges are available at (http://gssr.info/trends).
