# Supplementary material for: Long-term trends in storm surge climate derived from an ensemble of global surge reconstructions
Source: Sci Rep. 2022 Aug 3;12:13307. doi: 10.1038/s41598-022-17099-x (PMC9349196; doi:10.1038/s41598-022-17099-x)
Supplement: Supplementary file 1 — Supplementary Information. [file 41598_2022_17099_MOESM1_ESM.docx]

# Supplementary Figures


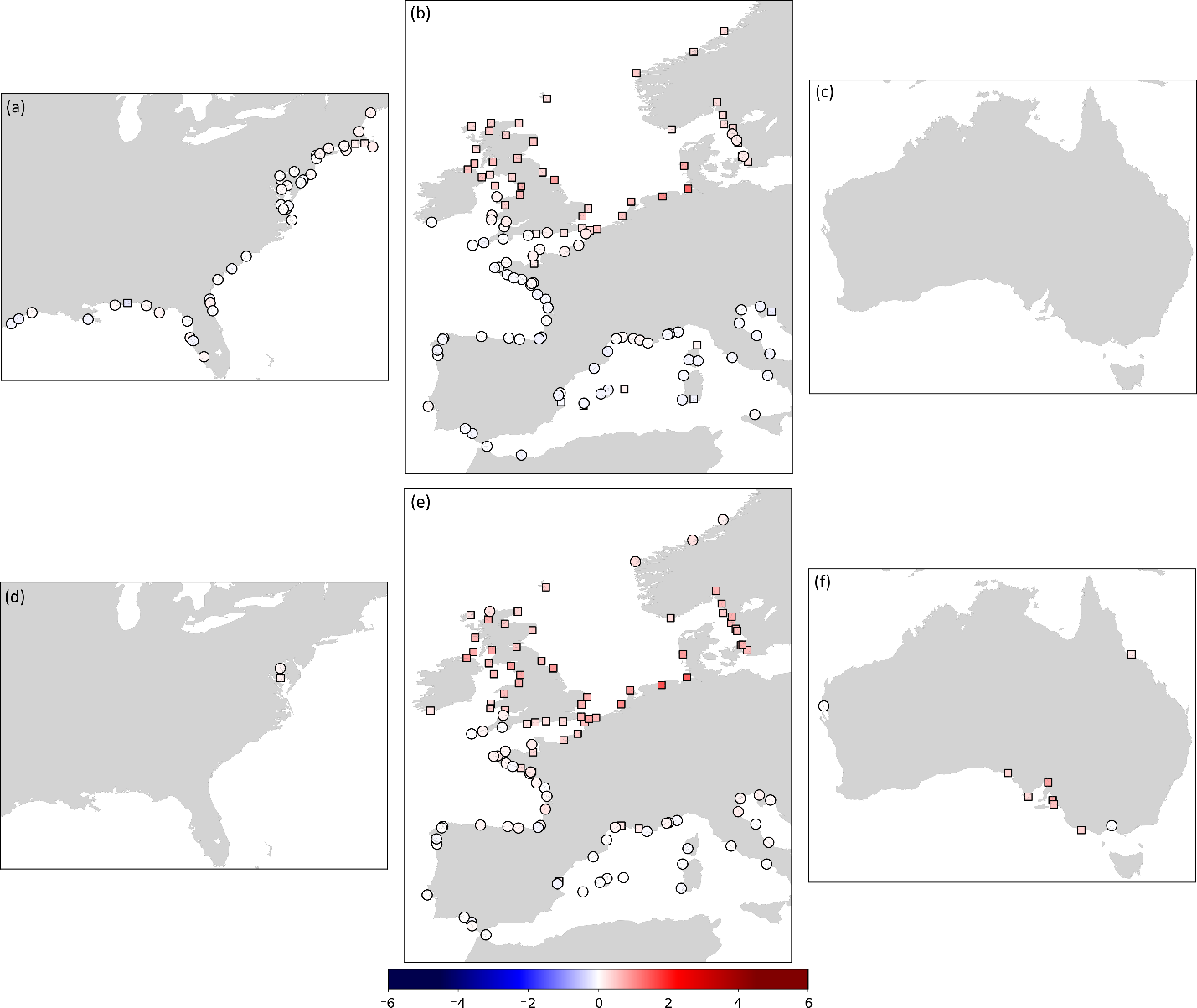


Supplementary Figure S1. Trends (mm/year) for the 95th percentile surges for G-20CR (a-c) and G-E20C (d-f) corresponding to the 1930-2015 and 1930-2010 respectively. Rectangle markers indicate significant trends at the 5% significance level. Rossum, Guido van, et al, The Python Language Reference, Python
Software Foundation; <http://docs.python.org/py3k/reference/index.html>


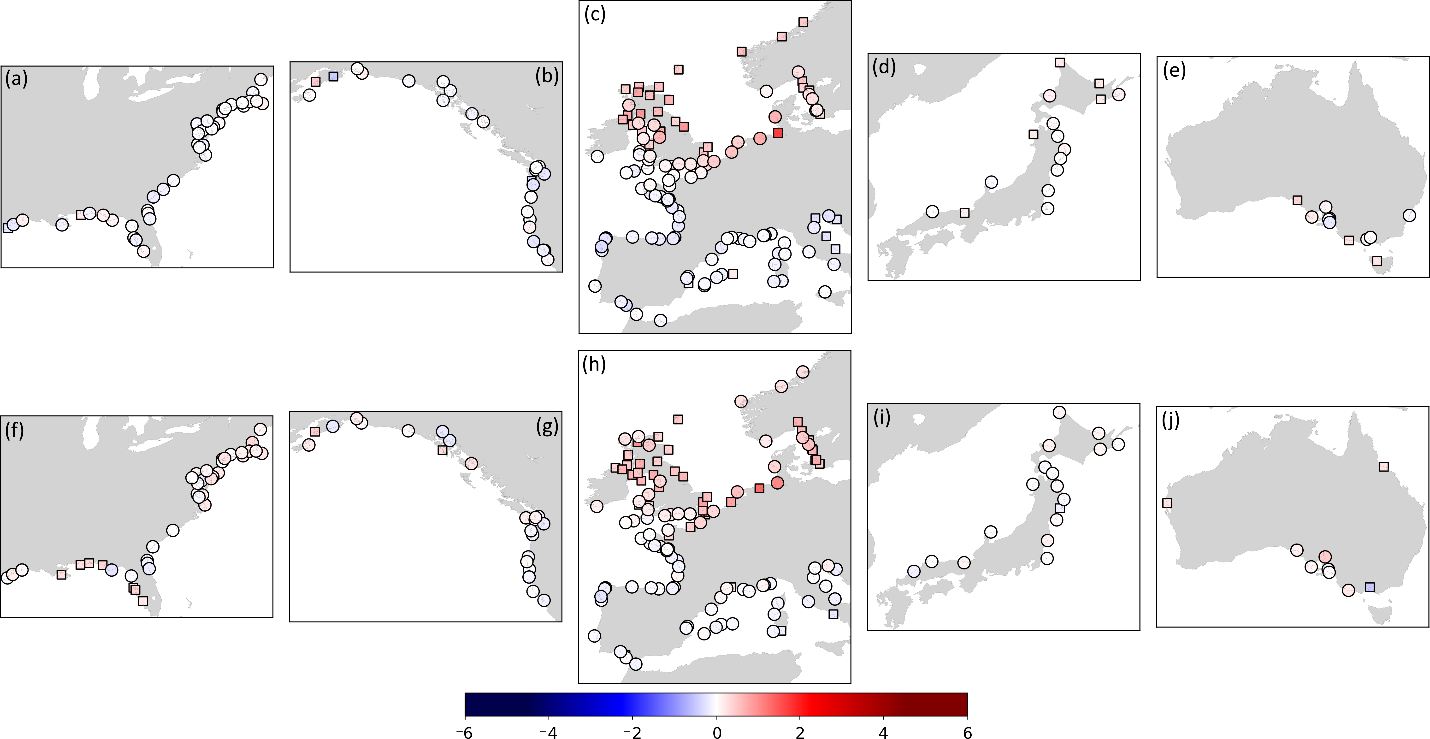


Supplementary Figure S2. Trends (mm/year) for the 95th percentile surges for G-20CR (a-e) and G-E20C (f-j) corresponding to 1950-2015 and 1950-2010 respectively. Rectangle markers indicate significant trends at the 5% significance level. Rossum, Guido van, et al, The Python Language Reference, Python
Software Foundation; <http://docs.python.org/py3k/reference/index.html>


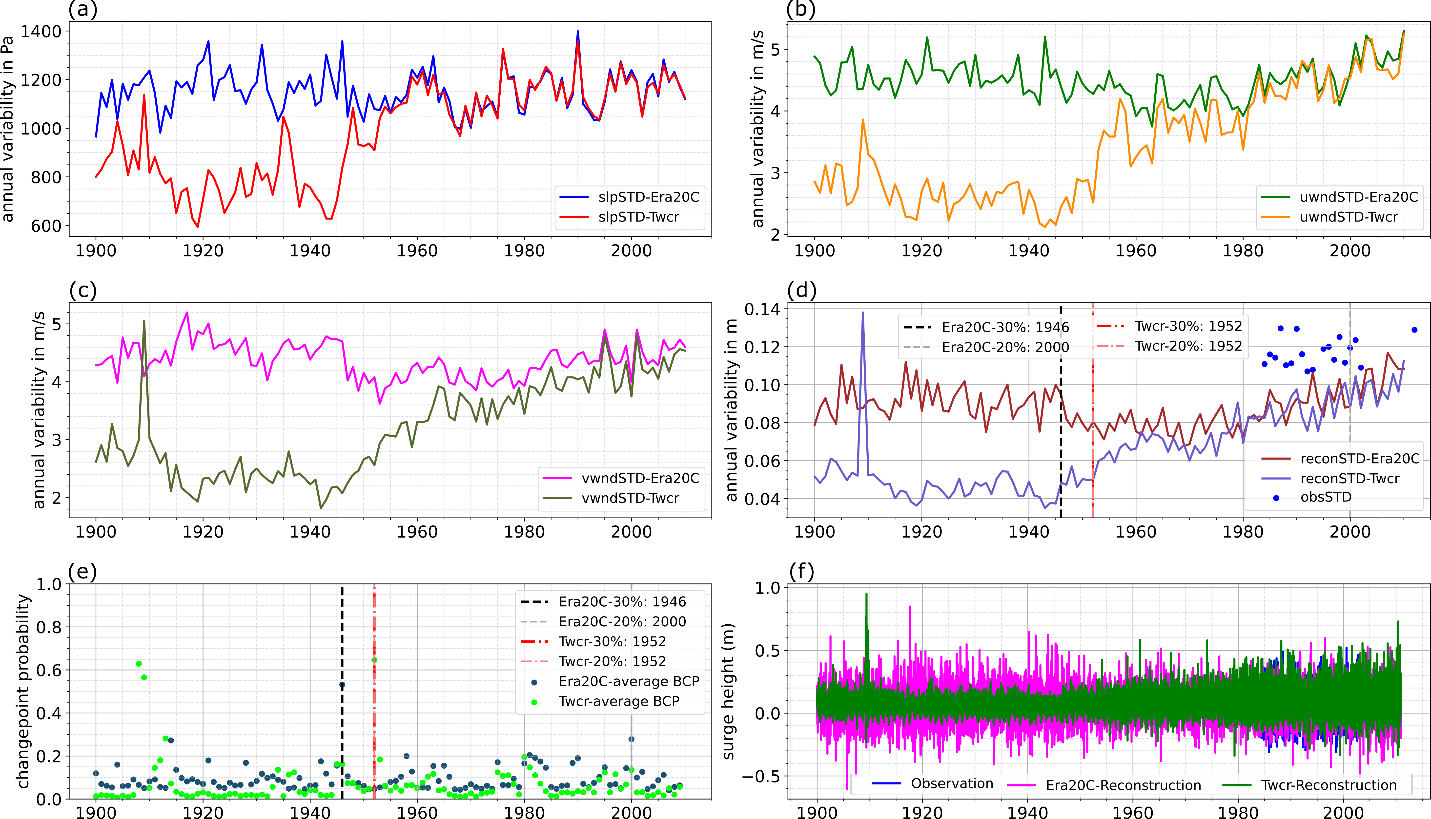


Supplementary Figure S3. Comparison of change point analysis for G-20CR and G-E20C for the 1900-2010 period for Antarctica Base Prat


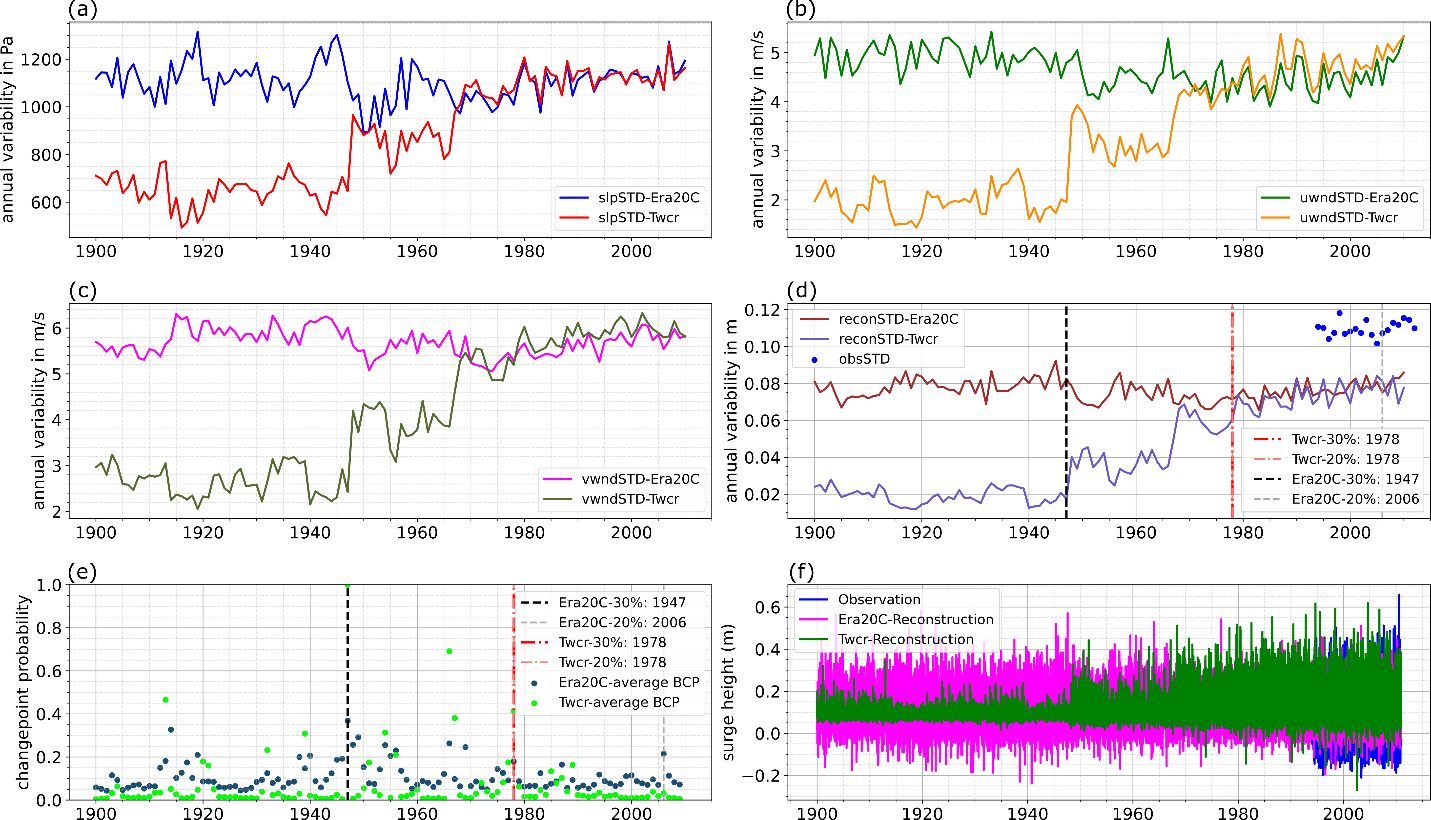


Supplementary Figure S4. Comparison of change point analysis for G-20CR and G-E20C for the 1900-2010 period for Kerguelen Island


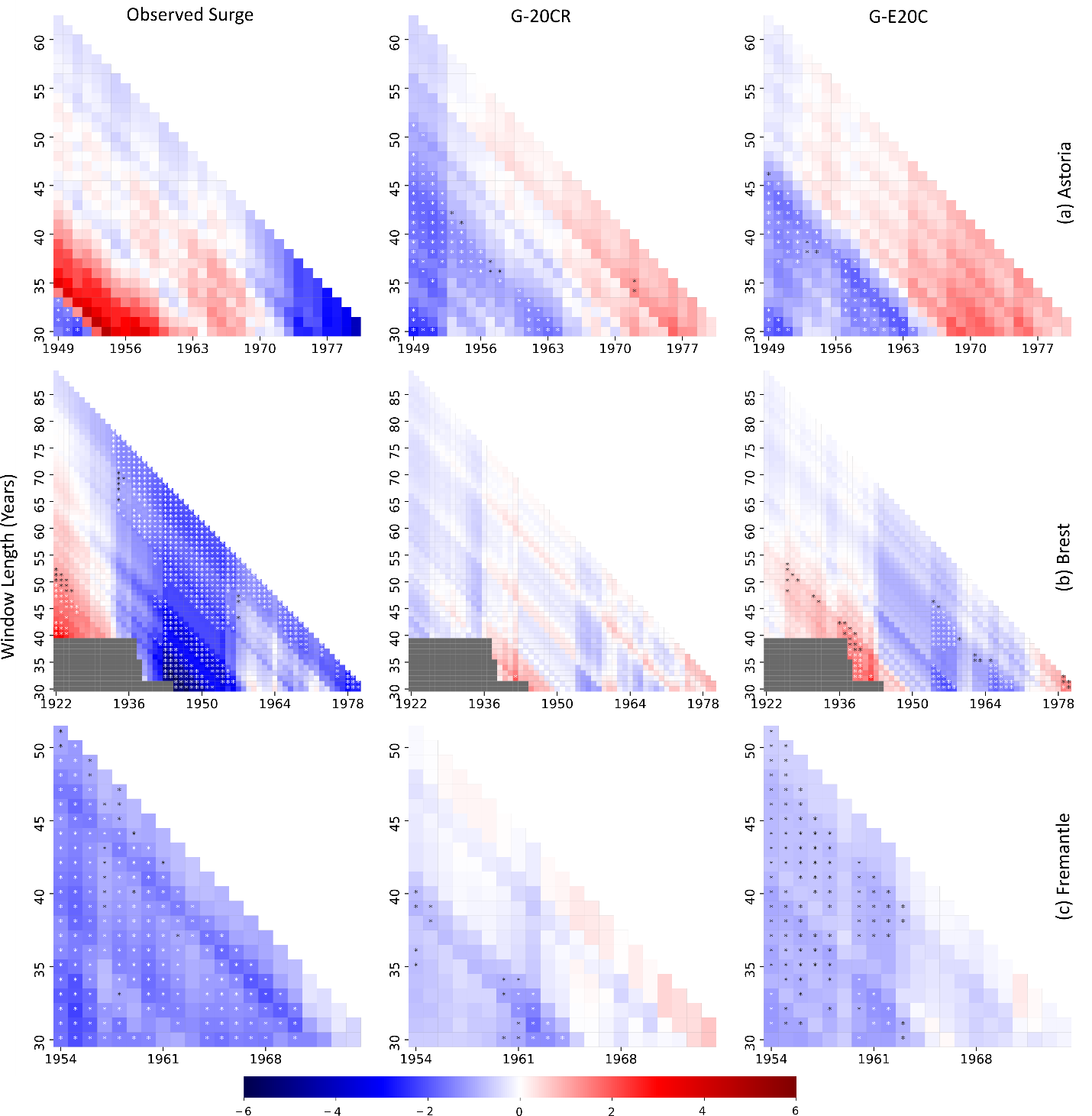


Supplementary Figure S5. Trends (mm/year) comparison for 99^th^ percentile observed surge (left), G-20CR (middle), and G-E20C (right) for Astoria (a), Brest (b), and Fremantle (c). Trends are computed starting with a minimum window length of 30 years up to the length of available data. Significant trends at 5% significance level are marked with an asterisk. Grey rectangles indicate time period where data is not at least 75% complete.


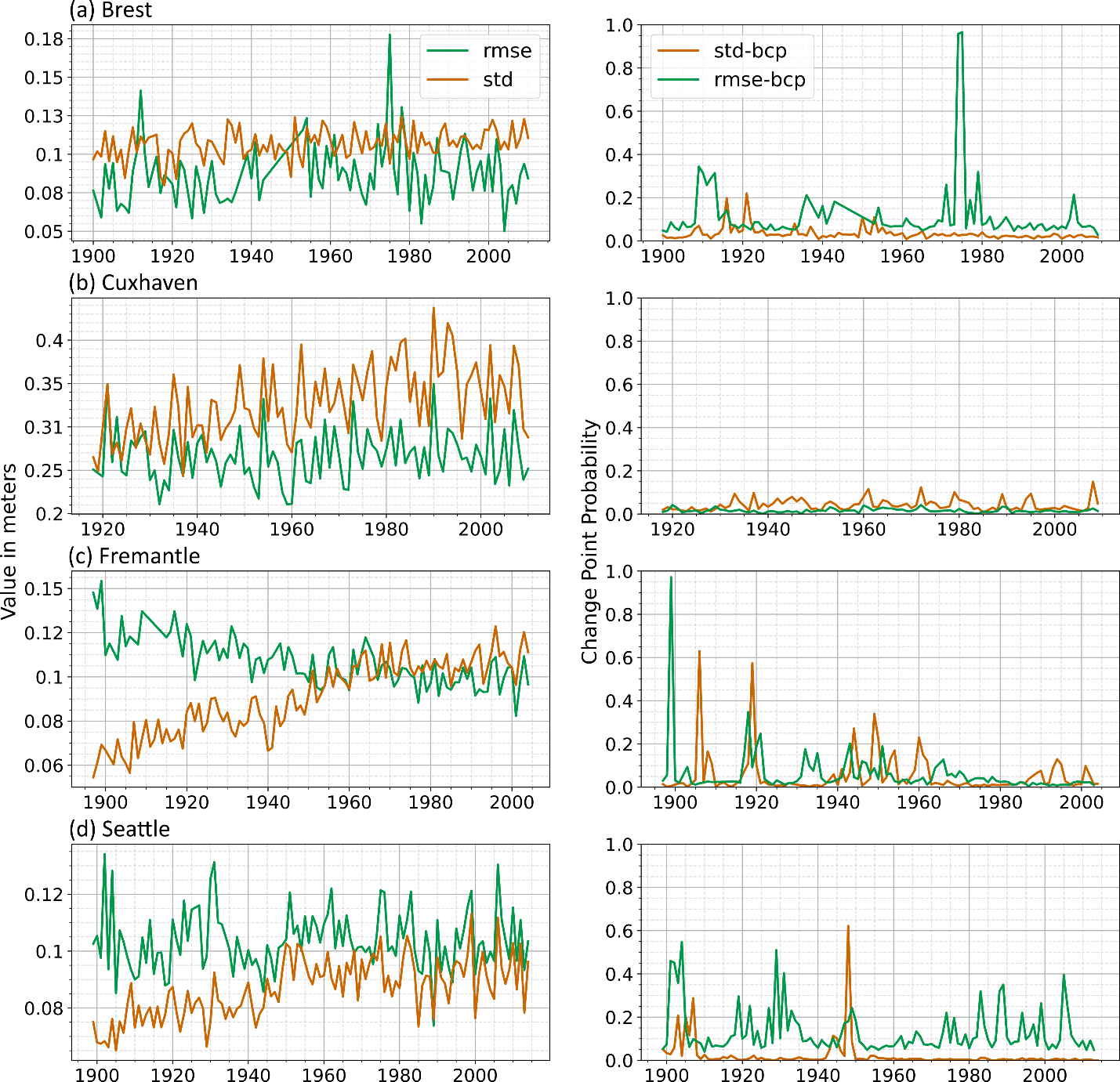


Supplementary Figure S6. Annual RMSE (green) and Standard Deviation (std; orange) time series (left) and the corresponding change point probabilities (right) for selected tide gauges with long observational records.


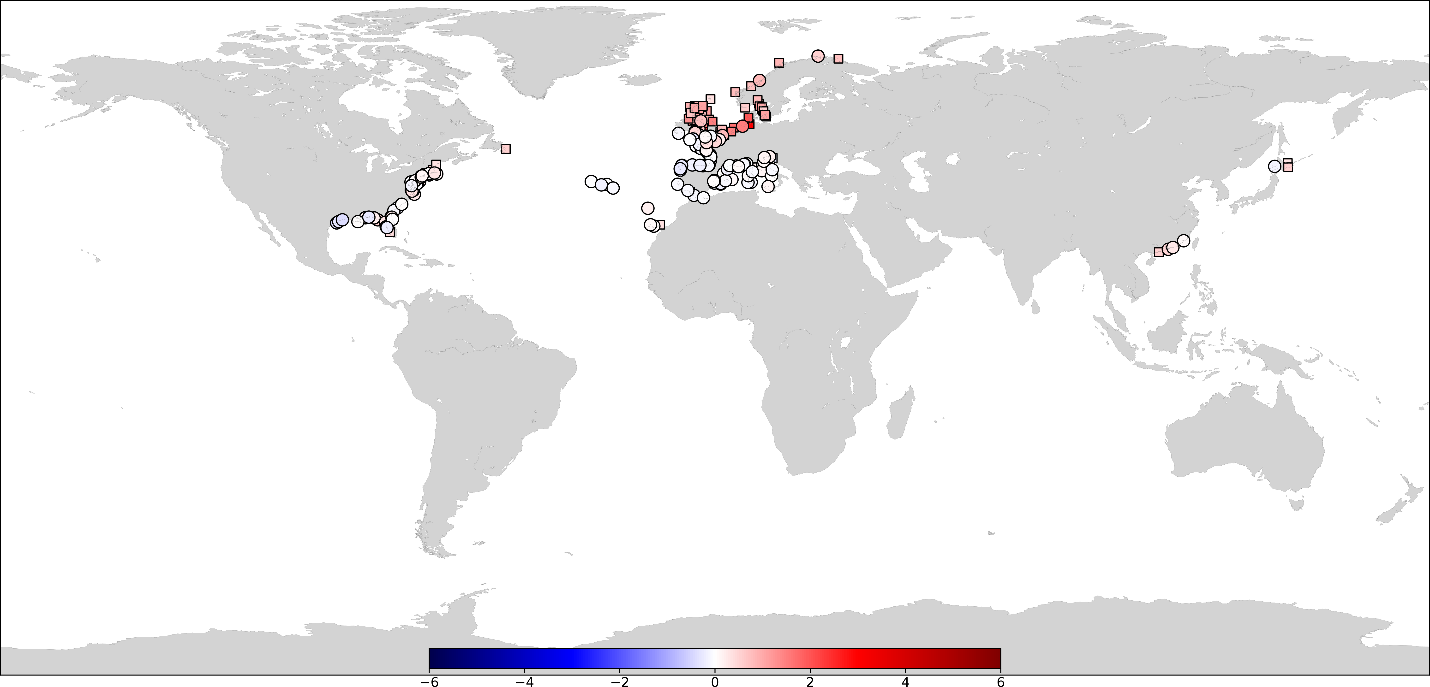


Supplementary Figure S7. Global Trends (mm/year) for the 99th percentile surges for G-20CR corresponding to the 1950-2015 period. Rectangle markers indicate significant trends at the 5% significance level. Rossum, Guido van, et al, The Python Language Reference, Python
Software Foundation; <http://docs.python.org/py3k/reference/index.html>


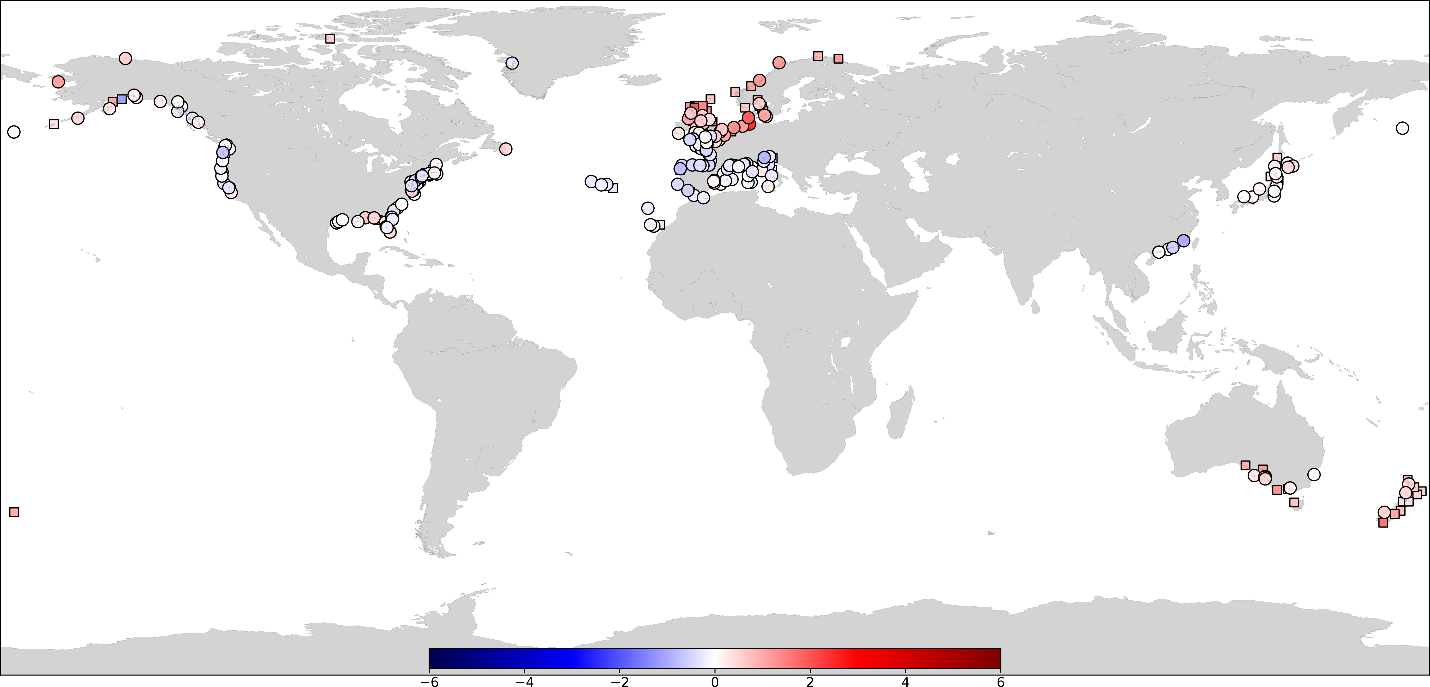


Supplementary Figure S8. Global Trends (mm/year) for the 99th percentile surges for G-20CR corresponding to the 1950-2015 period. Rectangle markers indicate significant trends at the 5% significance level. Rossum, Guido van, et al, The Python Language Reference, Python
Software Foundation; <http://docs.python.org/py3k/reference/index.html>


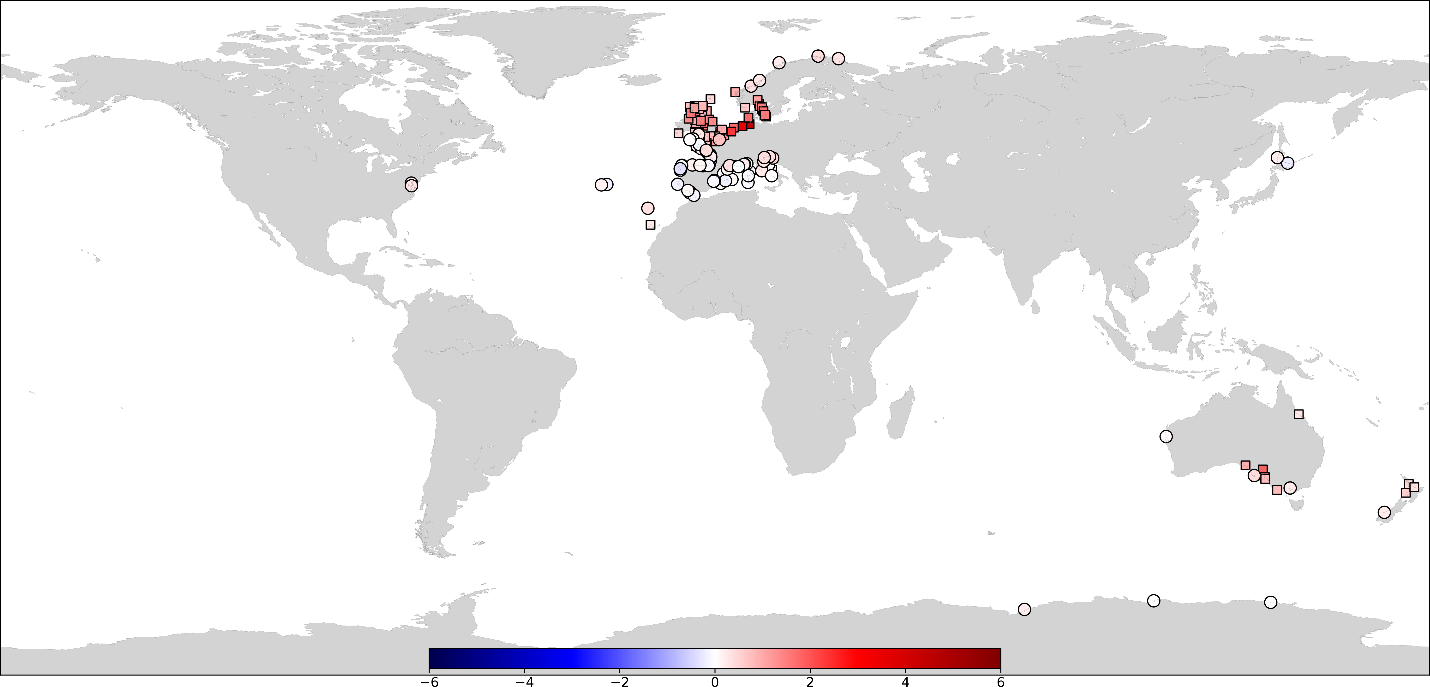


Supplementary Figure S9. Global Trends (mm/year) for the 99th percentile surges for G-E20C corresponding to the 1930-2015 period. Rectangle markers indicate significant trends at the 5% significance level. Rossum, Guido van, et al, The Python Language Reference, Python
Software Foundation; <http://docs.python.org/py3k/reference/index.html>


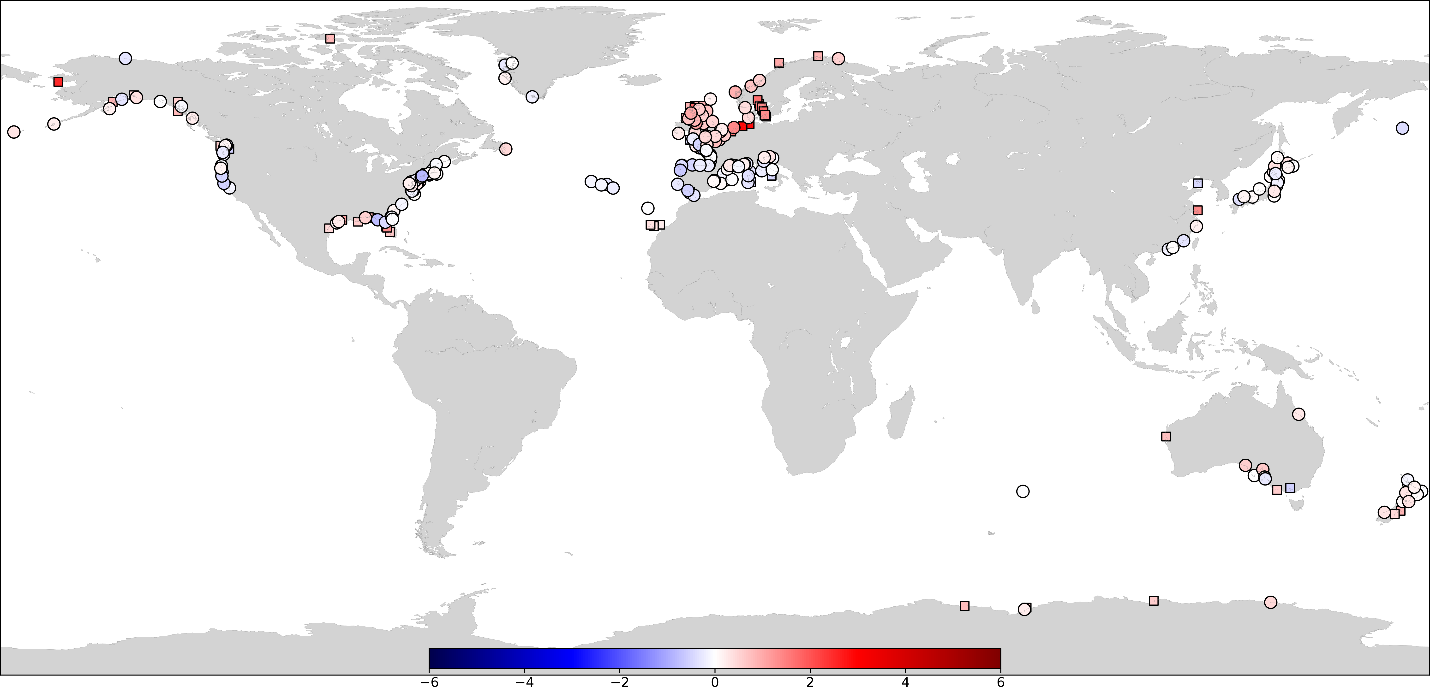


Supplementary Figure S10. Global Trends (mm/year) for the 99th percentile surges for G-E20C corresponding to the 1950-2015 period. Rectangle markers indicate significant trends at the 5% significance level. Rossum, Guido van, et al, The Python Language Reference, Python
Software Foundation; <http://docs.python.org/py3k/reference/index.html>

Supplementary Table 1. Global trends for 99^th^ percentile surges for G-20CR for the 1930-2015 period

| **Tide Gauge** | **longitude** | **latitude** | **Trend (mm/year)** | **P value** |
| --- | --- | --- | --- | --- |
| abashiri_japan | 144.28 | 44.017 | 0.289940385 | 0.017349523 |
| aberdeen_p038_uk | -2.0745 | 57.143 | 1.144014084 | 8.99E-05 |
| ajaccio_aspretto_france | 8.7628 | 41.923 | 0.012149695 | 0.911853105 |
| alcudia_alcudia_spain | 3.1392 | 39.835 | -0.070458489 | 0.538224912 |
| ancona_ancona_italy | 13.506 | 43.625 | 0.049345571 | 0.7666085 |
| andenes_001 | 16.15 | 69.317 | 0.858963646 | 0.033650318 |
| angra_heroismo_b_215b_portugal | -27.24 | 38.65 | -0.017998071 | 0.843390799 |
| annapolis_navalacademy__usa | -76.482 | 38.983 | -0.023155197 | 0.900252413 |
| apalachicola_usa | -84.982 | 29.727 | 0.522964749 | 0.126481515 |
| arcachon_eyrac_ | -1.1636 | 44.665 | 0.071661592 | 0.801456251 |
| atlantic_city_264a_usa | -74.418 | 39.355 | 0.307902791 | 0.098868252 |
| bakar_bakar_I_croatia | 14.533 | 45.3 | -0.320518784 | 0.01959595 |
| baltimore_usa | -76.578 | 39.267 | -0.000596107 | 0.997671705 |
| bangor_p662_uk | -5.6695 | 54.665 | 1.441752109 | 0.000169469 |
| barcelona_barcelona_spain | 2.163 | 41.342 | 0.045485795 | 0.715145854 |
| barmouth_p923_uk | -4.0452 | 52.719 | 0.687693088 | 0.081675613 |
| barseback_001_sweden | 12.903 | 55.756 | 0.505245606 | 0.010067827 |
| bayonne_boucau_ | -1.5148 | 43.527 | -0.128007816 | 0.611904046 |
| bilbao_bilbao_spain | -3.05 | 43.357 | 0.226692681 | 0.254087007 |
| bonanza_bonanza_spain | -6.34 | 36.8 | -0.438905309 | 0.045085534 |
| boston,ma_741a_usa | -71.052 | 42.355 | 0.589824374 | 0.005075254 |
| boulogne_sur_mer_ | 1.5777 | 50.727 | 0.852504751 | 0.018887931 |
| bournemouth_p988_uk | -1.8749 | 50.714 | 0.063917653 | 0.717120933 |
| brest_ | -4.5 | 48.383 | -0.032597863 | 0.887460609 |
| bridgeport_usa | -73.182 | 41.173 | 0.07645369 | 0.678582869 |
| cagliari_cagliari_italy | 9.1143 | 39.21 | -0.100168067 | 0.302343791 |
| calais_ | 1.8677 | 50.969 | 0.803550417 | 0.075026266 |
| cambridgeii_usa | -76.068 | 38.573 | -0.012535576 | 0.937700379 |
| capemay_usa | -74.96 | 38.968 | 0.178073665 | 0.59649064 |
| cape_may,nj_746a_usa | -74.96 | 38.968 | 0.01539177 | 0.940807125 |
| carloforte_carloforte_italy | 8.3095 | 39.148 | -0.07055577 | 0.475234178 |
| cascais_209a_portugal | -9.4167 | 38.692 | -0.05404915 | 0.725998754 |
| castletownsend_835a_ireland | -9.1833 | 51.533 | -0.077037522 | 0.672668595 |
| cedarkeyii_usa | -83.032 | 29.135 | 0.319914514 | 0.237967434 |
| centuri_france | 9.3498 | 42.966 | 0.210784636 | 0.00781629 |
| ceuta_ceta_spain | -5.3167 | 35.9 | -0.091239666 | 0.575605977 |
| charleston,sc_261a_usa | -79.925 | 32.782 | -0.150828823 | 0.364770019 |
| cherbourg_ | -1.6355 | 49.651 | 0.354770232 | 0.093966049 |
| chesapeake_bbt_749a_usa | -76.113 | 36.967 | 0.443965037 | 0.144868042 |
| civitavecchia_civitavecchia_italy | 11.79 | 42.094 | 0.1045229 | 0.295091275 |
| clearwater_bch,fl_773a_usa | -82.832 | 27.977 | 0.122876563 | 0.633805715 |
| concarneau_ | -3.9072 | 47.874 | 0.125457593 | 0.540695061 |
| coruna_coru_spain | -8.4 | 43.367 | -0.24798009 | 0.130185259 |
| cuxhaven_germany | 8.7167 | 53.867 | 3.006152788 | 0.005273308 |
| dauphin_island,_al_763a_usa | -88.075 | 30.25 | 0.199351837 | 0.532951806 |
| delfzijl_del_nl | 6.9331 | 53.326 | 1.51658619 | 0.059304211 |
| denhelder_hel_nl | 4.7464 | 52.965 | 1.684306391 | 0.00238712 |
| devonport_p002_uk | -4.1852 | 50.368 | -0.051786158 | 0.635794211 |
| dieppe_ | 1.0845 | 49.929 | 0.212479996 | 0.53490356 |
| donges_ | -2.0883 | 47.306 | 0.08805501 | 0.785936266 |
| dover_p012_uk | 1.3181 | 51.117 | 0.649574955 | 0.003593676 |
| duck_pier,nc_260a_usa | -75.74 | 36.183 | 0.259382798 | 0.16035592 |
| dunkerque_ | 2.3667 | 51.048 | 1.156425931 | 0.01849067 |
| esbjerg_130121_denmark | 8.4333 | 55.467 | 1.97460537 | 0.026344737 |
| felixstowe_p204_uk | 1.3484 | 51.957 | 0.847075148 | 0.014851444 |
| fernandina_beach_240a_usa | -81.467 | 30.672 | -0.149262015 | 0.575413574 |
| ferrol_ferrol_spain | -8.326 | 43.463 | -0.094510147 | 0.664906411 |
| fishguard_p055_uk | -4.9833 | 52.014 | 0.018522466 | 0.94691114 |
| flores,santa_cruz_210a_portugal | -31.168 | 39.378 | -0.034593356 | 0.791335381 |
| formentera_formentera_spain | 1.4189 | 38.735 | -0.087158603 | 0.47606104 |
| fortmyers_usa | -81.871 | 26.648 | 0.415824763 | 0.01831218 |
| fortpulaski_usa | -80.902 | 32.033 | 0.053986988 | 0.788381487 |
| fort_pulaski,ga_752a_usa | -80.902 | 32.033 | -0.071079045 | 0.764253275 |
| fos_sur_mer_france | 4.8929 | 43.405 | 0.064191568 | 0.027512797 |
| freeport_usa | -95.308 | 28.948 | -0.412633077 | 0.28588058 |
| fuerteventura_fuerteventura_spain | -13.85 | 28.5 | 0.340286132 | 2.58E-08 |
| funchal_b_218b_portugal | -16.907 | 32.64 | 0.160690107 | 0.399764604 |
| galveston,pier_21_775a_usa | -94.79 | 29.287 | -0.310951429 | 0.438932036 |
| gandia_gandia_spain | -0.152 | 38.995 | -0.081189141 | 0.419349445 |
| gijon_gijon_spain | -5.698 | 43.558 | -0.164508801 | 0.369950677 |
| gladstone_p234_uk | -3.018 | 53.45 | 2.268155653 | 7.27E-05 |
| gloucesterpoint_usa | -76.5 | 37.247 | 0.16156735 | 0.516440869 |
| goteborgtorshamnen_005_sweden | 11.8 | 57.683 | 0.50468808 | 0.01891273 |
| grandisle_usa | -89.957 | 29.263 | 0.042054503 | 0.806922199 |
| heimsjoe_001_norway | 9.1014 | 63.425 | 0.725066231 | 0.021267947 |
| heysham_p050_uk | -2.9204 | 54.032 | 1.492112651 | 0.008506345 |
| hoekvanholla_hvh_nl | 4.12 | 51.977 | 1.454829782 | 0.009770163 |
| holyhead_p054_uk | -4.6312 | 53.308 | 0.569191209 | 0.0170858 |
| hong_kong_b_329b_china | 114.2 | 22.3 | 0.515051775 | 0.054956041 |
| honningsvaag_002_norway | 25.973 | 70.98 | 0.525732702 | 0.092871677 |
| hornbaek_838a_denmark | 12.467 | 56.1 | 0.870446486 | 0.017916499 |
| horta,azores_212a_portugal | -28.622 | 38.533 | -0.142724565 | 0.35170125 |
| huelva_huelva_spain | -6.834 | 37.132 | -0.073763111 | 0.76223489 |
| ibiza_ibiza_spain | 1.4497 | 38.911 | -0.185191769 | 0.098967882 |
| ilfracombe_p061_uk | -4.1109 | 51.211 | 0.098942101 | 0.453938099 |
| imperia_imperia_italy | 8.0188 | 43.878 | -0.025318059 | 0.806832347 |
| kinlochbervi_p918_uk | -5.0504 | 58.457 | 0.909338756 | 0.005823262 |
| kiptopekebeach_usa | -75.988 | 37.165 | 0.106204733 | 0.683947162 |
| kungsvik_009_sweden | 11.127 | 58.997 | 0.586481037 | 0.020318342 |
| kushiro_japan | 144.38 | 42.967 | 0.527255572 | 0.00018142 |
| las_palmas_laspalmas_spain | -15.412 | 28.141 | 0.090106869 | 0.124000148 |
| la_coruna_830a_spain | -8.4 | 43.367 | -0.268222099 | 0.0536578 |
| la_rochelle_la_palli_ | -1.2206 | 46.158 | -0.170904964 | 0.494041946 |
| leith_p034_uk | -3.1817 | 55.99 | 0.825056021 | 0.008252143 |
| lerwick_p041_uk | -1.1333 | 60.15 | 0.370702644 | 0.041284234 |
| les_sables_d_olonne_ | -1.7935 | 46.497 | -0.30975864 | 0.279437054 |
| lewes,de_747a_usa | -75.12 | 38.782 | -0.081889441 | 0.667533105 |
| le_conquet_ | -4.7807 | 48.359 | -0.010722806 | 0.949957698 |
| le_crouesty_ | -2.8952 | 47.543 | 0.207531197 | 0.510383005 |
| le_havre_ | 0.106 | 49.482 | 0.439197807 | 0.124051721 |
| lowestoft_p024_uk | 1.7508 | 52.473 | 0.782192052 | 0.001037599 |
| maaloey_003_norway | 5.1133 | 61.934 | 0.79213625 | 0.000661564 |
| mahon_mahon_spain | 4.2706 | 39.893 | 0.070766079 | 0.670232327 |
| marseille_france | 5.35 | 43.3 | 0.349387392 | 0.189038546 |
| mayport,fl_753a_usa | -81.432 | 30.395 | 0.240818925 | 0.355424558 |
| melilla_melilla_spain | -2.918 | 35.291 | -0.037574766 | 0.759987614 |
| milfordhaven_p056_uk | -5.0143 | 51.702 | 0.508949394 | 0.082897796 |
| millport_p049_uk | -4.9058 | 55.75 | 0.996274569 | 0.00226951 |
| monaco_port_hercule_france | 7.4215 | 43.729 | -0.07792913 | 0.47479336 |
| montauk,ny_279a_usa | -71.96 | 41.048 | 0.159212994 | 0.452534827 |
| morayfirth_p207_uk | -4.0022 | 57.599 | 1.053240811 | 0.000431795 |
| mumbles_p932_uk | -3.9754 | 51.57 | 0.517994693 | 0.011256453 |
| nantucketisland_usa | -70.097 | 41.285 | 0.103949651 | 0.733088688 |
| napoli_napoli_italy | 14.269 | 40.841 | 0.015474279 | 0.89618302 |
| newhaven_p011_uk | 0.05703 | 50.782 | 0.636673024 | 0.017115369 |
| newlondon_usa | -72.09 | 41.361 | -0.112549224 | 0.542482694 |
| newlyn_p001_uk | -5.5417 | 50.102 | -0.251390318 | 0.097443588 |
| newport,ri_253a_usa | -71.327 | 41.505 | 0.337426882 | 0.037312715 |
| newport_usa | -71.327 | 41.505 | 0.369079172 | 0.013223684 |
| newyork_thebattery__usa | -74.014 | 40.701 | 0.030812704 | 0.907592759 |
| new_london,ct_744a_usa | -72.087 | 41.355 | 0.022053248 | 0.927798296 |
| new_york,ny_745a_usa | -74.015 | 40.7 | -0.0196778 | 0.954596731 |
| nice_france | 7.2853 | 43.696 | -0.06443606 | 0.639895257 |
| northshields_p032_uk | -1.4398 | 55.007 | 0.845731919 | 0.00207785 |
| ortona_ortona_italy | 14.415 | 42.356 | -0.078326337 | 0.508513906 |
| oslo_004_norway | 10.734 | 59.909 | 0.82065785 | 0.001993549 |
| otaru_japan | 141 | 43.2 | -0.102499325 | 0.725038566 |
| palermo_palermo_italy | 13.371 | 38.121 | 0.133687438 | 0.261930711 |
| palma_de_mallorca_palmademallorca_spain | 2.6375 | 39.56 | -0.057693904 | 0.657069683 |
| panama_city_beach_761a_usa | -85.88 | 30.213 | 0.201050137 | 0.367542997 |
| pensacola,fl_762a_usa | -87.213 | 30.403 | -0.259707142 | 0.093212911 |
| ponta_delgada_211a_portugal | -25.672 | 37.735 | -0.089760109 | 0.286396972 |
| portellen_p202_uk | -6.1901 | 55.627 | 1.453861758 | 9.63E-05 |
| porterin_p919_uk | -4.7681 | 54.085 | 1.316105374 | 1.75E-05 |
| portland_maine__usa | -70.247 | 43.657 | 0.372360785 | 0.037433105 |
| porto_torres_porto+torres_italy | 8.4039 | 40.842 | 0.069367391 | 0.60564986 |
| portpatrick_p063_uk | -5.12 | 54.843 | 0.877496409 | 0.001855193 |
| portrush_p935_uk | -6.6568 | 55.207 | 1.288983555 | 0.005642798 |
| portsmouth_p008_uk | -1.1118 | 50.803 | -0.049281975 | 0.782362768 |
| port_bloc_ | -1.0616 | 45.569 | -0.031200343 | 0.88765191 |
| port_camargue_france | 4.1264 | 43.52 | 0.156972751 | 0.021504589 |
| port_tudy_ | -3.4459 | 47.644 | -0.063357996 | 0.785810994 |
| port_vendres_france | 3.1075 | 42.52 | -0.161850777 | 0.213588719 |
| ravenna_ravenna_italy | 12.283 | 44.492 | 0.062908156 | 0.753253097 |
| reedypoint_usa | -75.573 | 39.558 | 0.073212388 | 0.762540989 |
| ringhals_016_sweden | 12.113 | 57.25 | 0.516767013 | 0.031674523 |
| roervik_005_norway | 11.23 | 64.859 | 0.818530284 | 0.078190736 |
| roscoff_ | -3.9657 | 48.718 | -0.095291128 | 0.622459354 |
| sabine_pass_n,tx_766a_usa | -93.87 | 29.73 | -0.420678561 | 0.168179107 |
| sagunto_sagunto_spain | -0.206 | 39.634 | -0.137741229 | 0.176748306 |
| saint_gildas_ | -2.2464 | 47.14 | 0.099578766 | 0.760132046 |
| saint_jean_de_luz_so_ | -1.6816 | 43.395 | -0.081905496 | 0.609697052 |
| saint_malo_ | -2.0281 | 48.641 | 0.475068513 | 0.019497304 |
| saint_nazaire_ | -2.2016 | 47.267 | 0.09416884 | 0.750645746 |
| sandyhook_usa | -74.009 | 40.467 | 0.27675371 | 0.316291442 |
| santander_ieo_spain | -3.79 | 43.461 | -0.179471243 | 0.136048544 |
| sete_france | 3.6991 | 43.398 | -0.035883425 | 0.758860192 |
| sewellspoint,hamptonroads_usa | -76.33 | 36.947 | 0.345591314 | 0.223030133 |
| shanwei_641a_china | 115.35 | 22.75 | 0.279264468 | 0.539457237 |
| smogen_020_sweden | 11.218 | 58.354 | 1.098402768 | 6.31E-05 |
| solenzara_france | 9.4038 | 41.857 | -0.065293079 | 0.651078786 |
| solomonsisland_biol.lab.__usa | -76.452 | 38.317 | -0.119106961 | 0.408286053 |
| springmaidpier_usa | -78.918 | 33.655 | 0.038573394 | 0.728075806 |
| st.petersburg_usa | -82.627 | 27.761 | -0.411090262 | 0.138108999 |
| st._augustine,fl_262a_usa | -81.262 | 29.857 | -0.025624679 | 0.900204667 |
| st._john_s_b_276b_canada | -52.7 | 47.567 | 0.526972313 | 0.008460166 |
| st._petersburg,_fl_759a_usa | -82.627 | 27.76 | -0.222045272 | 0.354296074 |
| stenungsund_022_sweden | 11.832 | 58.093 | 0.994543493 | 0.001441825 |
| sthelier_p074_uk | -2.1167 | 49.183 | 0.331390959 | 0.201390486 |
| stmarys_p231_uk | -6.3164 | 49.918 | -0.020574997 | 0.909741526 |
| stornoway_p042_uk | -6.3882 | 58.208 | 0.91324735 | 8.54E-05 |
| tenerife_tenerife_spain | -16.24 | 28.478 | 0.052449581 | 0.424327026 |
| tobermory_p223_uk | -6.0642 | 56.623 | 0.667630774 | 0.022247443 |
| toulon_france | 5.9131 | 43.117 | 0.087901224 | 0.239395025 |
| tregde_006_norway | 7.5666 | 58 | 0.580195831 | 0.028239621 |
| trieste_270061_italy | 13.75 | 45.65 | 0.15075169 | 0.546953673 |
| ullapool_p043_uk | -5.1579 | 57.895 | 1.008616891 | 0.030595817 |
| valencia_valencia_spain | -0.33 | 39.46 | 0.019815425 | 0.890620762 |
| varberg1_024_sweden | 12.217 | 57.1 | 0.517266468 | 0.003153423 |
| vardo,norway_001 | 31.1 | 70.333 | 0.709302026 | 0.002218425 |
| venezia_punta_salute_italy | 12.426 | 45.419 | 0.098809479 | 0.7826941 |
| vigo_ieo_spain | -8.7333 | 42.233 | -0.230563392 | 0.184501607 |
| viken_025_sweden | 12.579 | 56.142 | 1.103315984 | 0.000217534 |
| villagarcia_villagarcia_spain | -8.77 | 42.601 | -0.259853967 | 0.271772069 |
| weymouth_p991_uk | -2.4479 | 50.608 | 0.062061528 | 0.747176068 |
| whitby_p174_uk | -0.61417 | 54.49 | 1.511985609 | 0.001268187 |
| wick_p035_uk | -3.0863 | 58.441 | 1.08556122 | 0.001184309 |
| willetspoint_usa | -73.782 | 40.793 | 0.105338934 | 0.636090455 |
| woods_hole,ma_742a_usa | -70.672 | 41.523 | 0.314961738 | 0.213908585 |
| workington_p217_uk | -3.5676 | 54.651 | 0.732285697 | 0.090583157 |
| xiamen_376a_china | 118.07 | 24.45 | 0.073278458 | 0.90603039 |
| zhapo_a_635a_china | 111.83 | 21.583 | 0.540912519 | 0.004060775 |

Supplementary Table 2. Global trends for 99^th^ percentile surges for G-20CR for the 1950-2015 period

| **Tide Gauge** | **lon** | **lat** | **Trend (mm/year)** | **P value** |
| --- | --- | --- | --- | --- |
| abashiri_japan | 144.28 | 44.017 | 0.172658599 | 0.257031648 |
| aberdeen_p038_uk | -2.0745 | 57.143 | 1.246101033 | 0.000347049 |
| adak,alaska_040a_usa | -176.63 | 51.863 | 0.092797046 | 0.663155449 |
| ajaccio_aspretto_france | 8.7628 | 41.923 | -0.137797851 | 0.453453173 |
| alameda_navalairstation__usa | -122.3 | 37.772 | -0.228949108 | 0.292812933 |
| alcudia_alcudia_spain | 3.1392 | 39.835 | -0.171869949 | 0.401546525 |
| ancona_ancona_italy | 13.506 | 43.625 | -0.318890754 | 0.231011594 |
| andenes_001 | 16.15 | 69.317 | 1.085457952 | 0.074313382 |
| angra_heroismo_b_215b_portugal | -27.24 | 38.65 | -0.230883185 | 0.197297221 |
| annapolis_navalacademy__usa | -76.482 | 38.983 | -0.16908289 | 0.450433063 |
| apalachicola_usa | -84.982 | 29.727 | 1.039764797 | 0.025119567 |
| arcachon_eyrac_ | -1.1636 | 44.665 | -0.387755543 | 0.112107032 |
| arena_cove,_ca_573a_usa | -123.71 | 38.913 | -0.369630754 | 0.336556675 |
| astoria,or_572a_usa | -123.77 | 46.208 | -0.449873997 | 0.176675699 |
| atlantic_city_264a_usa | -74.418 | 39.355 | 0.31104151 | 0.266514944 |
| auckland_070a_new_zealand | 174.77 | -36.85 | 0.411448804 | 0.121906068 |
| ayukawa_japan | 141.5 | 38.3 | 0.218407547 | 0.194187336 |
| bakar_bakar_I_croatia | 14.533 | 45.3 | -0.573074809 | 0.00023767 |
| baltimore_usa | -76.578 | 39.267 | 0.025202533 | 0.9311469 |
| bangor_p662_uk | -5.6695 | 54.665 | 1.075640712 | 0.044597631 |
| barcelona_barcelona_spain | 2.163 | 41.342 | 0.041502335 | 0.835214825 |
| barmouth_p923_uk | -4.0452 | 52.719 | 1.094898855 | 0.056343177 |
| barseback_001_sweden | 12.903 | 55.756 | 0.660375486 | 0.056579419 |
| bayonne_boucau_ | -1.5148 | 43.527 | -0.441044991 | 0.064186746 |
| bilbao_bilbao_spain | -3.05 | 43.357 | -0.144389551 | 0.504595692 |
| bluff_072a_new_zealand | 168.35 | -46.6 | 1.623592624 | 3.78E-10 |
| bonanza_bonanza_spain | -6.34 | 36.8 | -0.790443499 | 0.03209947 |
| boston,ma_741a_usa | -71.052 | 42.355 | 0.835053951 | 0.006000148 |
| boulogne_sur_mer_ | 1.5777 | 50.727 | 1.278777179 | 0.016071705 |
| bournemouth_p988_uk | -1.8749 | 50.714 | 0.242919812 | 0.289796308 |
| brest_ | -4.5 | 48.383 | -0.071649416 | 0.825407757 |
| bridgeport_usa | -73.182 | 41.173 | -0.079520608 | 0.728901748 |
| burnie_344a_australia | 145.91 | -41.5 | 0.677903562 | 0.000133054 |
| cagliari_cagliari_italy | 9.1143 | 39.21 | -0.214445318 | 0.175843693 |
| calais_ | 1.8677 | 50.969 | 0.858061027 | 0.226304055 |
| cambridgeii_usa | -76.068 | 38.573 | -0.210335732 | 0.340625495 |
| capemay_usa | -74.96 | 38.968 | 0.218574767 | 0.637832454 |
| cape_may,nj_746a_usa | -74.96 | 38.968 | -0.111792292 | 0.699990545 |
| carloforte_carloforte_italy | 8.3095 | 39.148 | -0.092092399 | 0.584835672 |
| cascais_209a_portugal | -9.4167 | 38.692 | -0.382560233 | 0.15618484 |
| castletownsend_835a_ireland | -9.1833 | 51.533 | 0.210787658 | 0.422557922 |
| cedarkeyii_usa | -83.032 | 29.135 | 0.312805033 | 0.497114213 |
| centuri_france | 9.3498 | 42.966 | 0.120379915 | 0.362451684 |
| ceuta_ceta_spain | -5.3167 | 35.9 | -0.148100692 | 0.530850038 |
| charleston,sc_261a_usa | -79.925 | 32.782 | -0.485361252 | 0.110651978 |
| chatham_079a_new_zealand | -176.56 | -43.947 | 0.95819074 | 1.07E-13 |
| cherbourg_ | -1.6355 | 49.651 | 0.580352878 | 0.007257369 |
| chesapeake_bbt_749a_usa | -76.113 | 36.967 | -0.149031349 | 0.78504522 |
| choshigyoko_japan | 140.87 | 35.75 | 0.09911373 | 0.687165896 |
| civitavecchia_civitavecchia_italy | 11.79 | 42.094 | 0.198644195 | 0.211008979 |
| clearwater_bch,fl_773a_usa | -82.832 | 27.977 | -0.081463322 | 0.812177805 |
| concarneau_ | -3.9072 | 47.874 | -0.079695702 | 0.78299277 |
| cordova_b,alaska_583b_usa | -145.75 | 60.558 | 0.308732738 | 0.322882352 |
| coruna_coru_spain | -8.4 | 43.367 | -0.695262056 | 0.016825295 |
| crescent_city,ca_556a_usa | -124.18 | 41.745 | -0.237105365 | 0.425725522 |
| cuxhaven_germany | 8.7167 | 53.867 | 2.482553384 | 0.103015893 |
| dauphin_island,_al_763a_usa | -88.075 | 30.25 | 0.634967764 | 0.162812203 |
| delfzijl_del_nl | 6.9331 | 53.326 | 1.12957159 | 0.270549514 |
| denhelder_hel_nl | 4.7464 | 52.965 | 1.309771351 | 0.067378975 |
| devonport_p002_uk | -4.1852 | 50.368 | -0.013367781 | 0.930162242 |
| dieppe_ | 1.0845 | 49.929 | 0.255894773 | 0.601546519 |
| donges_ | -2.0883 | 47.306 | -0.517165289 | 0.203786753 |
| dover_p012_uk | 1.3181 | 51.117 | 0.561622365 | 0.034098437 |
| duck_pier,nc_260a_usa | -75.74 | 36.183 | 0.113316884 | 0.697374796 |
| dunkerque_ | 2.3667 | 51.048 | 1.063932295 | 0.115737125 |
| dutch_harbor_b,ak_041b_usa | -166.54 | 53.88 | 0.451209485 | 0.014665428 |
| esbjerg_130121_denmark | 8.4333 | 55.467 | 1.890063445 | 0.074865434 |
| felixstowe_p204_uk | 1.3484 | 51.957 | 0.695214098 | 0.16663637 |
| fernandina_beach_240a_usa | -81.467 | 30.672 | -0.433212337 | 0.236215163 |
| ferrol_ferrol_spain | -8.326 | 43.463 | -0.497880102 | 0.253689719 |
| fishguard_p055_uk | -4.9833 | 52.014 | 0.149912068 | 0.748219798 |
| flores,santa_cruz_210a_portugal | -31.168 | 39.378 | -0.276014906 | 0.198736821 |
| formentera_formentera_spain | 1.4189 | 38.735 | -0.07935761 | 0.648226719 |
| fortmyers_usa | -81.871 | 26.648 | 0.39466038 | 0.097001644 |
| fortpulaski_usa | -80.902 | 32.033 | -0.231096871 | 0.444060938 |
| fort_pulaski,ga_752a_usa | -80.902 | 32.033 | -0.29843528 | 0.409776363 |
| fos_sur_mer_france | 4.8929 | 43.405 | 0.045984881 | 0.303051733 |
| freeport_usa | -95.308 | 28.948 | -0.263150037 | 0.522019134 |
| fridayharbor_ocean.labs.__usa | -123.01 | 48.547 | 0.168480192 | 0.527149578 |
| fuerteventura_fuerteventura_spain | -13.85 | 28.5 | 0.272672988 | 0.001127353 |
| fukaura_japan | 139.93 | 40.65 | 0.365974334 | 0.042176251 |
| funchal_b_218b_portugal | -16.907 | 32.64 | -0.196793688 | 0.519393544 |
| galveston,pier_21_775a_usa | -94.79 | 29.287 | -0.046394821 | 0.903359034 |
| gandia_gandia_spain | -0.152 | 38.995 | -0.157908891 | 0.324833692 |
| geelong_013_australia | 144.36 | -38.147 | 0.499601213 | 0.021207662 |
| gijon_gijon_spain | -5.698 | 43.558 | -0.432516908 | 0.14535908 |
| gisborne_b_078b_new_zealand | 178.03 | -38.683 | 0.545763123 | 0.000550366 |
| gladstone_p234_uk | -3.018 | 53.45 | 1.860525804 | 0.017961259 |
| gloucesterpoint_usa | -76.5 | 37.247 | 0.499378103 | 0.149370372 |
| goteborgtorshamnen_005_sweden | 11.8 | 57.683 | 0.71720534 | 0.012770513 |
| grandisle_usa | -89.957 | 29.263 | 0.076780463 | 0.700208438 |
| hachinohe_japan | 141.53 | 40.533 | 0.144199859 | 0.238733693 |
| hanasaki_japan | 145.57 | 43.283 | 0.37692809 | 0.139190695 |
| heimsjoe_001_norway | 9.1014 | 63.425 | 1.06770827 | 0.000562247 |
| heysham_p050_uk | -2.9204 | 54.032 | 0.820204701 | 0.283938226 |
| hoekvanholla_hvh_nl | 4.12 | 51.977 | 1.574018803 | 0.011456943 |
| holyhead_p054_uk | -4.6312 | 53.308 | 0.662814718 | 0.067414073 |
| hong_kong_b_329b_china | 114.2 | 22.3 | -0.044833841 | 0.887071669 |
| honningsvaag_002_norway | 25.973 | 70.98 | 0.840671213 | 0.004490818 |
| hornbaek_838a_denmark | 12.467 | 56.1 | 0.999201099 | 0.115841685 |
| horta,azores_212a_portugal | -28.622 | 38.533 | -0.183028173 | 0.242263335 |
| huelva_huelva_spain | -6.834 | 37.132 | -0.547044561 | 0.080572753 |
| humboldt_bay,_ca_576a_usa | -124.22 | 40.767 | -0.008789425 | 0.97141343 |
| ibiza_ibiza_spain | 1.4497 | 38.911 | -0.314121889 | 0.043792083 |
| ilfracombe_p061_uk | -4.1109 | 51.211 | -0.077080854 | 0.677302889 |
| ilulissat,greenland_001 | -51.1 | 69.217 | -0.257660334 | 0.537451546 |
| imperia_imperia_italy | 8.0188 | 43.878 | -0.182757015 | 0.205637079 |
| jackson_403a_new_zealand | 168.62 | -43.983 | 0.478024299 | 0.090910797 |
| juneau_usa | -134.41 | 58.298 | -0.052845682 | 0.813996268 |
| ketchikan,ak_571a_usa | -131.63 | 55.333 | -0.163796642 | 0.587195579 |
| kinlochbervi_p918_uk | -5.0504 | 58.457 | 1.366349331 | 0.004384564 |
| kiptopekebeach_usa | -75.988 | 37.165 | -0.217287267 | 0.458218249 |
| kodiak_isl.,alaska_039a_usa | -152.51 | 57.732 | 0.244108527 | 0.150838563 |
| kungsvik_009_sweden | 11.127 | 58.997 | 0.586995976 | 0.052948658 |
| kushiro_japan | 144.38 | 42.967 | 0.398583664 | 0.062020209 |
| l._cornwallis_i._705a_canada | -96.95 | 75.383 | 0.517286398 | 0.000770109 |
| las_palmas_laspalmas_spain | -15.412 | 28.141 | 0.070551047 | 0.487463087 |
| la_coruna_830a_spain | -8.4 | 43.367 | -0.613364298 | 0.009472754 |
| la_rochelle_la_palli_ | -1.2206 | 46.158 | -0.234683432 | 0.497015567 |
| leith_p034_uk | -3.1817 | 55.99 | 0.596824105 | 0.141749435 |
| lerwick_p041_uk | -1.1333 | 60.15 | 0.669018747 | 0.009126812 |
| les_sables_d_olonne_ | -1.7935 | 46.497 | -0.791982193 | 0.02239136 |
| lewes,de_747a_usa | -75.12 | 38.782 | -0.24986408 | 0.153857098 |
| le_conquet_ | -4.7807 | 48.359 | -0.268615602 | 0.28998245 |
| le_crouesty_ | -2.8952 | 47.543 | -0.151458225 | 0.702827886 |
| le_havre_ | 0.106 | 49.482 | 0.398402881 | 0.415020297 |
| lowestoft_p024_uk | 1.7508 | 52.473 | 0.623214049 | 0.124427605 |
| lyttelton_667a_new_zealand | 172.72 | -43.6 | 0.69766453 | 0.000103617 |
| maaloey_003_norway | 5.1133 | 61.934 | 0.662065039 | 0.020776992 |
| mahon_mahon_spain | 4.2706 | 39.893 | -0.192767899 | 0.371161258 |
| maizuru_japan | 135.38 | 35.467 | 0.111563118 | 0.529584164 |
| marsden_point_398a_new_zealand | 174.5 | -35.833 | 0.778247383 | 0.019842364 |
| marseille_france | 5.35 | 43.3 | 0.022572254 | 0.950069036 |
| massacre_bay,ak_550a_usa | 173.2 | 52.833 | -0.015449491 | 0.957279634 |
| mayport,fl_753a_usa | -81.432 | 30.395 | 0.092400914 | 0.839910395 |
| melilla_melilla_spain | -2.918 | 35.291 | -0.164246413 | 0.338374581 |
| milfordhaven_p056_uk | -5.0143 | 51.702 | 0.211395431 | 0.554797215 |
| millport_p049_uk | -4.9058 | 55.75 | 0.8099474 | 0.026254469 |
| miyako_japan | 141.98 | 39.65 | 0.297275524 | 0.008572434 |
| monaco_port_hercule_france | 7.4215 | 43.729 | -0.26027378 | 0.118934614 |
| montauk,ny_279a_usa | -71.96 | 41.048 | -0.160749896 | 0.54413671 |
| monterey,ca_555a_usa | -121.89 | 36.605 | 0.300210338 | 0.205952352 |
| morayfirth_p207_uk | -4.0022 | 57.599 | 0.982594828 | 0.019329281 |
| mumbles_p932_uk | -3.9754 | 51.57 | 0.174714158 | 0.536064193 |
| nantucketisland_usa | -70.097 | 41.285 | -0.094058926 | 0.738158763 |
| napier_668a_new_zealand | 176.92 | -39.483 | 0.48067917 | 0.000456762 |
| napoli_napoli_italy | 14.269 | 40.841 | -0.257707775 | 0.15820678 |
| nelson_077a_new_zealand | 173.27 | -41.267 | 0.310295196 | 0.023813758 |
| newhaven_p011_uk | 0.05703 | 50.782 | 0.603138089 | 0.118489199 |
| newlondon_usa | -72.09 | 41.361 | -0.352127098 | 0.199232382 |
| newlyn_p001_uk | -5.5417 | 50.102 | -0.331278689 | 0.237401958 |
| newport,ri_253a_usa | -71.327 | 41.505 | 0.100510506 | 0.637808973 |
| newport_usa | -71.327 | 41.505 | 0.165319298 | 0.532894139 |
| newyork_thebattery__usa | -74.014 | 40.701 | 0.027603762 | 0.938010006 |
| new_london,ct_744a_usa | -72.087 | 41.355 | -0.321210057 | 0.338894338 |
| new_york,ny_745a_usa | -74.015 | 40.7 | 0.179263592 | 0.700361109 |
| nice_france | 7.2853 | 43.696 | -0.264815344 | 0.251815601 |
| nome,ak_595a_usa | -165.43 | 64.5 | 1.046983169 | 0.113613708 |
| northshields_p032_uk | -1.4398 | 55.007 | 0.372569998 | 0.375947105 |
| noto_japan | 137.15 | 37.5 | 0.11234788 | 0.762795359 |
| ofunato_japan | 141.72 | 39.067 | 0.204782429 | 0.017556557 |
| onahama_japan | 140.9 | 36.933 | -0.09069285 | 0.670139008 |
| ortona_ortona_italy | 14.415 | 42.356 | -0.295687155 | 0.009487199 |
| oslo_004_norway | 10.734 | 59.909 | 0.881174563 | 0.00662817 |
| otaru_japan | 141 | 43.2 | 0.191641638 | 0.578026021 |
| palermo_palermo_italy | 13.371 | 38.121 | 0.148399479 | 0.366441379 |
| palma_de_mallorca_palmademallorca_spain | 2.6375 | 39.56 | -0.109704637 | 0.612386666 |
| panama_city_beach_761a_usa | -85.88 | 30.213 | 0.507189872 | 0.135856071 |
| pensacola,fl_762a_usa | -87.213 | 30.403 | -0.518901927 | 0.001508422 |
| ponta_delgada_211a_portugal | -25.672 | 37.735 | -0.306670796 | 0.017585902 |
| portadelaideouter_020_australia | 138.6 | -34.926 | -0.253042409 | 0.438679989 |
| portellen_p202_uk | -6.1901 | 55.627 | 1.759669149 | 0.000761125 |
| porterin_p919_uk | -4.7681 | 54.085 | 1.269257303 | 0.005628176 |
| portland,s.aus._129a_australia | 141.6 | -38.333 | 1.230582631 | 9.90E-08 |
| portland_maine__usa | -70.247 | 43.657 | 0.079768812 | 0.751912196 |
| portlincoln_023_australia | 135.86 | -34.72 | 0.310844109 | 0.233689221 |
| porto_torres_porto+torres_italy | 8.4039 | 40.842 | 0.062362248 | 0.771767712 |
| portpatrick_p063_uk | -5.12 | 54.843 | 0.812865049 | 0.028720844 |
| portpirie_024_australia | 138.01 | -33.177 | 1.11014464 | 0.008329048 |
| portrush_p935_uk | -6.6568 | 55.207 | 0.993094662 | 0.100220922 |
| portsmouth_p008_uk | -1.1118 | 50.803 | -0.393579371 | 0.160995836 |
| port_angeles,_wa_584a_usa | -123.44 | 48.125 | 0.079159867 | 0.68825338 |
| port_bloc_ | -1.0616 | 45.569 | -0.625535963 | 0.01374646 |
| port_camargue_france | 4.1264 | 43.52 | 0.0253067 | 0.799472906 |
| port_kembla_342a_australia | 150.91 | -34.473 | 0.032815079 | 0.840002054 |
| port_orford,_or_557a_usa | -124.5 | 42.74 | -0.184487327 | 0.429016201 |
| port_stanvac_100a_australia | 138.47 | -35.108 | 0.439887299 | 0.315270358 |
| port_tudy_ | -3.4459 | 47.644 | -0.094820205 | 0.759348966 |
| port_vendres_france | 3.1075 | 42.52 | -0.305924743 | 0.206390623 |
| princerupert,canada_001 | -130.17 | 54.317 | 0.171780453 | 0.470193093 |
| prudhoe_bay,_ak_579a_usa | -148.53 | 70.4 | 0.531458831 | 0.087805907 |
| ravenna_ravenna_italy | 12.283 | 44.492 | -0.352570114 | 0.280480868 |
| reedypoint_usa | -75.573 | 39.558 | -0.341841463 | 0.364045458 |
| ringhals_016_sweden | 12.113 | 57.25 | 0.860238166 | 0.020239902 |
| roervik_005_norway | 11.23 | 64.859 | 1.136093539 | 0.082630221 |
| roscoff_ | -3.9657 | 48.718 | -0.038933898 | 0.883427719 |
| sabine_pass_n,tx_766a_usa | -93.87 | 29.73 | -0.022400415 | 0.933306122 |
| sagunto_sagunto_spain | -0.206 | 39.634 | -0.120746737 | 0.464959742 |
| saint_gildas_ | -2.2464 | 47.14 | -0.199783953 | 0.665601337 |
| saint_jean_de_luz_so_ | -1.6816 | 43.395 | -0.386700404 | 0.015229476 |
| saint_malo_ | -2.0281 | 48.641 | 0.182103915 | 0.554067403 |
| saint_nazaire_ | -2.2016 | 47.267 | -0.448041743 | 0.242164374 |
| sakai_japan | 133.25 | 35.55 | 0.006398497 | 0.977131296 |
| sandyhook_usa | -74.009 | 40.467 | -0.2805269 | 0.4281983 |
| sand_point,ak_574a_usa | -160.5 | 55.337 | 0.418426232 | 0.063699254 |
| sanfrancisco_usa | -122.47 | 37.807 | -0.326491202 | 0.056999836 |
| santander_ieo_spain | -3.79 | 43.461 | -0.355029923 | 0.069512079 |
| seattle_usa | -122.34 | 47.603 | -0.157283422 | 0.433003191 |
| seldovia_usa | -151.72 | 59.44 | 0.900333979 | 0.025164413 |
| sete_france | 3.6991 | 43.398 | -0.254631785 | 0.051215026 |
| seward_c,ak_560c_usa | -149.43 | 60.12 | -1.059582952 | 0.018650287 |
| sewellspoint,hamptonroads_usa | -76.33 | 36.947 | 0.444953136 | 0.389560167 |
| shanwei_641a_china | 115.35 | 22.75 | -0.537971149 | 0.383623896 |
| shimokita_japan | 141.23 | 41.367 | 0.050391491 | 0.745024765 |
| sitka,ak_559a_usa | -135.34 | 57.052 | -0.260170056 | 0.415803012 |
| skagway_usa | -135.33 | 59.45 | 0.051330746 | 0.861238409 |
| smogen_020_sweden | 11.218 | 58.354 | 1.231004883 | 0.002118043 |
| solenzara_france | 9.4038 | 41.857 | -0.245033952 | 0.322583785 |
| solomonsisland_biol.lab.__usa | -76.452 | 38.317 | -0.306312998 | 0.17910156 |
| south_beach,or_592a_usa | -124.04 | 44.625 | -0.077398889 | 0.854041021 |
| springmaidpier_usa | -78.918 | 33.655 | -0.024308044 | 0.900830082 |
| st.petersburg_usa | -82.627 | 27.761 | -0.172927459 | 0.403658366 |
| st._augustine,fl_262a_usa | -81.262 | 29.857 | -0.188675509 | 0.531514564 |
| st._john_s_b_276b_canada | -52.7 | 47.567 | 0.462627557 | 0.185109008 |
| st._petersburg,_fl_759a_usa | -82.627 | 27.76 | -0.169343814 | 0.532180696 |
| stenungsund_022_sweden | 11.832 | 58.093 | 1.236845879 | 0.006126676 |
| sthelier_p074_uk | -2.1167 | 49.183 | -0.006994191 | 0.986430854 |
| stmarys_p231_uk | -6.3164 | 49.918 | -0.449953255 | 0.172293144 |
| stornoway_p042_uk | -6.3882 | 58.208 | 1.277341363 | 0.000377314 |
| taranaki_076a_new_zealand | 174.03 | -39.05 | 0.430866013 | 0.140913869 |
| tauranga_073a_new_zealand | 176.18 | -37.65 | 0.581495323 | 0.000287388 |
| tenerife_tenerife_spain | -16.24 | 28.478 | -0.13161589 | 0.100764699 |
| thevenard_026_australia | 133.65 | -32.146 | 0.918228461 | 0.000147292 |
| timaru_665a_new_zealand | 171.25 | -44.383 | 0.992154065 | 3.50E-06 |
| tobermory_p223_uk | -6.0642 | 56.623 | 0.58953933 | 0.164582662 |
| toulon_france | 5.9131 | 43.117 | -0.075701793 | 0.484848897 |
| tregde_006_norway | 7.5666 | 58 | 0.654854935 | 0.021804256 |
| trieste_270061_italy | 13.75 | 45.65 | -0.190227396 | 0.464137195 |
| ullapool_p043_uk | -5.1579 | 57.895 | 1.88196591 | 0.004935742 |
| unalaska_usa | -166.54 | 53.88 | 0.306393541 | 0.023328149 |
| valdez,ak_562a_usa | -146.36 | 61.125 | 0.171751291 | 0.557659972 |
| valencia_valencia_spain | -0.33 | 39.46 | 0.117354761 | 0.643380725 |
| varberg1_024_sweden | 12.217 | 57.1 | 0.637282517 | 0.012764026 |
| vardo,norway_001 | 31.1 | 70.333 | 1.153018512 | 2.23E-05 |
| venezia_punta_salute_italy | 12.426 | 45.419 | -0.81301337 | 0.060100871 |
| victorharbor_027_australia | 138.62 | -35.553 | 0.441585295 | 0.322639732 |
| victoria,bc_543a_canada | -123.37 | 48.422 | -0.084290641 | 0.799021477 |
| vigo_ieo_spain | -8.7333 | 42.233 | -0.662421013 | 0.019664127 |
| viken_025_sweden | 12.579 | 56.142 | 1.589529865 | 0.000236822 |
| villagarcia_villagarcia_spain | -8.77 | 42.601 | -0.727251921 | 0.091963442 |
| wakkanai_japan | 141.68 | 45.4 | 0.589746973 | 0.000847561 |
| wellington_071a_new_zealand | 174.78 | -41.283 | 0.247582479 | 0.02833422 |
| weymouth_p991_uk | -2.4479 | 50.608 | 0.007477776 | 0.97807329 |
| whitby_p174_uk | -0.61417 | 54.49 | 1.889128197 | 0.001163184 |
| wick_p035_uk | -3.0863 | 58.441 | 1.404643233 | 0.000225748 |
| willapa_bay,_wa_564a_usa | -123.97 | 46.708 | -0.659852906 | 0.081403153 |
| willetspoint_usa | -73.782 | 40.793 | -0.356545827 | 0.235053242 |
| williamstown_028_australia | 144.9 | -37.857 | 0.14232009 | 0.533315245 |
| woods_hole,ma_742a_usa | -70.672 | 41.523 | 0.164745765 | 0.537071721 |
| workington_p217_uk | -3.5676 | 54.651 | 0.596455208 | 0.331158108 |
| xiamen_376a_china | 118.07 | 24.45 | -0.956727068 | 0.113610298 |
| yakutat_usa | -139.74 | 59.548 | 0.151882225 | 0.33219122 |
| zhapo_a_635a_china | 111.83 | 21.583 | -0.089167727 | 0.630371593 |

Supplementary Table 3. Global trends for 99^th^ percentile surges for G-E20C for the 1930-2015 period

| **Tide Gauge** | **longitude** | **latitude** | **Trend (mm/year)** | **P value** |
| --- | --- | --- | --- | --- |
| abashiri_japan | 144.28 | 44.017 | -0.201594105 | 0.316432193 |
| aberdeen_p038_uk | -2.0745 | 57.143 | 0.938043635 | 0.010949117 |
| ajaccio_aspretto_france | 8.7628 | 41.923 | -0.062582145 | 0.662504445 |
| alcudia_alcudia_spain | 3.1392 | 39.835 | 0.110381382 | 0.035733657 |
| ancona_ancona_italy | 13.506 | 43.625 | 0.003871654 | 0.981116331 |
| andenes_001 | 16.15 | 69.317 | 0.210562735 | 0.509693312 |
| angra_heroismo_b_215b_portugal | -27.24 | 38.65 | -0.140961934 | 0.31035983 |
| annapolis_navalacademy__usa | -76.482 | 38.983 | 0.240751674 | 0.191072538 |
| arcachon_eyrac_ | -1.1636 | 44.665 | 0.449842644 | 0.232009957 |
| auckland_070a_new_zealand | 174.77 | -36.85 | 0.394599229 | 0.000715548 |
| bakar_bakar_I_croatia | 14.533 | 45.3 | 0.448084791 | 0.124343782 |
| barcelona_barcelona_spain | 2.163 | 41.342 | 0.026525237 | 0.785886331 |
| barmouth_p923_uk | -4.0452 | 52.719 | 0.913086145 | 0.013050662 |
| barseback_001_sweden | 12.903 | 55.756 | 1.297967419 | 5.42E-05 |
| bayonne_boucau_ | -1.5148 | 43.527 | 0.123931345 | 0.657618915 |
| bilbao_bilbao_spain | -3.05 | 43.357 | 0.034632382 | 0.869232942 |
| bonanza_bonanza_spain | -6.34 | 36.8 | -0.250627036 | 0.32302646 |
| boulogne_sur_mer_ | 1.5777 | 50.727 | 1.17319578 | 0.000140784 |
| bournemouth_p988_uk | -1.8749 | 50.714 | 0.363408515 | 0.034942635 |
| brest_ | -4.5 | 48.383 | 0.032334152 | 0.877006155 |
| cadiz_cadi_spain | -6.2833 | 36.533 | -0.01467255 | 0.938039706 |
| calais_ | 1.8677 | 50.969 | 1.391885234 | 0.000122878 |
| cape_ferguson_343a_australia | 147.06 | -19.277 | 0.308879343 | 0.019187821 |
| carloforte_carloforte_italy | 8.3095 | 39.148 | -0.047207446 | 0.723639456 |
| carnarvon_008_australia | 113.66 | -24.884 | 0.055440382 | 0.779559071 |
| cascais_209a_portugal | -9.4167 | 38.692 | -0.173141515 | 0.501674361 |
| casey_130a_australia | 110.53 | -66.283 | 0.019259668 | 0.909166221 |
| castletownsend_835a_ireland | -9.1833 | 51.533 | 0.479317807 | 0.027288086 |
| ceuta_ceta_spain | -5.3167 | 35.9 | -0.135785879 | 0.198469909 |
| civitavecchia_civitavecchia_italy | 11.79 | 42.094 | 0.244389637 | 0.285739276 |
| concarneau_ | -3.9072 | 47.874 | 0.507452982 | 0.005354687 |
| cuxhaven_germany | 8.7167 | 53.867 | 4.541193639 | 7.42E-07 |
| davis_173a_australia | 77.967 | -68.45 | 0.199642669 | 0.074546216 |
| delfzijl_del_nl | 6.9331 | 53.326 | 3.597583673 | 1.21E-06 |
| denhelder_hel_nl | 4.7464 | 52.965 | 1.724993747 | 0.00186772 |
| devonport_p002_uk | -4.1852 | 50.368 | 0.065305222 | 0.583880863 |
| dieppe_ | 1.0845 | 49.929 | 0.707551051 | 0.050490529 |
| donges_ | -2.0883 | 47.306 | 1.014530392 | 0.007649608 |
| dover_p012_uk | 1.3181 | 51.117 | 1.676184825 | 0.000142191 |
| dumont_d_urville_189a_france | 140.01 | -66.662 | -0.015123957 | 0.952442531 |
| dunkerque_ | 2.3667 | 51.048 | 1.241820296 | 0.001571557 |
| esbjerg_130121_denmark | 8.4333 | 55.467 | 1.739793989 | 0.04797374 |
| felixstowe_p204_uk | 1.3484 | 51.957 | 1.428926044 | 0.026172432 |
| ferrol_ferrol_spain | -8.326 | 43.463 | 0.031480818 | 0.868974031 |
| fishguard_p055_uk | -4.9833 | 52.014 | 0.711840912 | 0.004142696 |
| funchal_b_218b_portugal | -16.907 | 32.64 | 0.330968534 | 0.08218086 |
| gijon_gijon_spain | -5.698 | 43.558 | 0.145883476 | 0.533425285 |
| gladstone_p234_uk | -3.018 | 53.45 | 1.521093581 | 1.06E-06 |
| goteborgtorshamnen_005_sweden | 11.8 | 57.683 | 1.83302077 | 4.01E-05 |
| heimsjoe_001_norway | 9.1014 | 63.425 | 0.349552944 | 0.239891803 |
| heysham_p050_uk | -2.9204 | 54.032 | 1.877620387 | 0.000297629 |
| hoekvanholla_hvh_nl | 4.12 | 51.977 | 2.366515586 | 1.05E-06 |
| honningsvaag_002_norway | 25.973 | 70.98 | 0.396475454 | 0.136336879 |
| hornbaek_838a_denmark | 12.467 | 56.1 | 1.407265719 | 3.30E-05 |
| horta,azores_212a_portugal | -28.622 | 38.533 | 0.157675473 | 0.224226124 |
| huelva_huelva_spain | -6.834 | 37.132 | 0.078541974 | 0.818147077 |
| ibiza_ibiza_spain | 1.4497 | 38.911 | 0.098130136 | 0.212432884 |
| ilfracombe_p061_uk | -4.1109 | 51.211 | 0.242922427 | 0.146198538 |
| imperia_imperia_italy | 8.0188 | 43.878 | -0.0515701 | 0.62154452 |
| jackson_403a_new_zealand | 168.62 | -43.983 | 0.232770012 | 0.066285808 |
| kinlochbervi_p918_uk | -5.0504 | 58.457 | 1.027687118 | 0.000452243 |
| kungsvik_009_sweden | 11.127 | 58.997 | 1.293633078 | 0.000611857 |
| la_coruna_830a_spain | -8.4 | 43.367 | -0.059395226 | 0.704701977 |
| la_rochelle_la_palli_ | -1.2206 | 46.158 | 0.127833395 | 0.618714483 |
| leith_p034_uk | -3.1817 | 55.99 | 0.872502635 | 0.000926521 |
| lerwick_p041_uk | -1.1333 | 60.15 | 0.429530388 | 0.045039846 |
| les_sables_d_olonne_ | -1.7935 | 46.497 | 0.267752215 | 0.314865359 |
| le_conquet_ | -4.7807 | 48.359 | 0.495933431 | 0.002993345 |
| le_crouesty_ | -2.8952 | 47.543 | 0.147145881 | 0.679310796 |
| le_havre_ | 0.106 | 49.482 | 1.187392945 | 1.16E-05 |
| lowestoft_p024_uk | 1.7508 | 52.473 | 0.943264852 | 0.004436065 |
| maaloey_003_norway | 5.1133 | 61.934 | 0.894929691 | 0.015779387 |
| mahon_mahon_spain | 4.2706 | 39.893 | 0.080364595 | 0.097253649 |
| marseille_france | 5.35 | 43.3 | 0.326063974 | 0.002671134 |
| milfordhaven_p056_uk | -5.0143 | 51.702 | 0.70579879 | 0.003056174 |
| millport_p049_uk | -4.9058 | 55.75 | 1.637863492 | 0.000724123 |
| monaco_port_hercule_france | 7.4215 | 43.729 | 0.007720645 | 0.950721891 |
| morayfirth_p207_uk | -4.0022 | 57.599 | 0.889540679 | 0.001922231 |
| mumbles_p932_uk | -3.9754 | 51.57 | 0.941743847 | 0.000549442 |
| napoli_napoli_italy | 14.269 | 40.841 | 0.027388819 | 0.822003007 |
| newhaven_p011_uk | 0.05703 | 50.782 | 0.894572737 | 0.002378349 |
| newlyn_p001_uk | -5.5417 | 50.102 | -0.034706225 | 0.786181569 |
| nice_france | 7.2853 | 43.696 | 0.213873045 | 0.089246843 |
| northshields_p032_uk | -1.4398 | 55.007 | 1.330222538 | 4.80E-06 |
| ortona_ortona_italy | 14.415 | 42.356 | 0.40302653 | 0.012965024 |
| oslo_004_norway | 10.734 | 59.909 | 1.089109761 | 0.014766366 |
| palma_de_mallorca_palmademallorca_spain | 2.6375 | 39.56 | -0.162687066 | 0.258861198 |
| portellen_p202_uk | -6.1901 | 55.627 | 1.270512111 | 0.001673314 |
| porterin_p919_uk | -4.7681 | 54.085 | 1.003073036 | 0.025127029 |
| portland,s.aus._129a_australia | 141.6 | -38.333 | 0.790056684 | 5.22E-07 |
| portlincoln_023_australia | 135.86 | -34.72 | 0.395547926 | 0.086354118 |
| portlonsdale_017_australia | 138.5 | -35.099 | 1.006460227 | 0.000116172 |
| porto_torres_porto+torres_italy | 8.4039 | 40.842 | -0.057090917 | 0.695219967 |
| portpatrick_p063_uk | -5.12 | 54.843 | 0.924957785 | 0.003456538 |
| portpirie_024_australia | 138.01 | -33.177 | 1.86342557 | 6.87E-07 |
| portrush_p935_uk | -6.6568 | 55.207 | 1.405006644 | 0.00199307 |
| portsmouth_p008_uk | -1.1118 | 50.803 | 0.793041274 | 0.000506782 |
| port_bloc_ | -1.0616 | 45.569 | 0.221185028 | 0.404928438 |
| port_camargue_france | 4.1264 | 43.52 | 0.180115624 | 0.002209232 |
| port_stanvac_100a_australia | 138.47 | -35.108 | 1.625905169 | 0.000133223 |
| port_tudy_ | -3.4459 | 47.644 | -0.087515502 | 0.618275926 |
| port_vendres_france | 3.1075 | 42.52 | 0.064174725 | 0.697673014 |
| ravenna_ravenna_italy | 12.283 | 44.492 | 0.359645166 | 0.118462365 |
| ringhals_016_sweden | 12.113 | 57.25 | 1.502138273 | 5.64E-05 |
| roervik_005_norway | 11.23 | 64.859 | 0.268882086 | 0.176954334 |
| roscoff_ | -3.9657 | 48.718 | -0.090270787 | 0.639070746 |
| sagunto_sagunto_spain | -0.206 | 39.634 | 0.031793001 | 0.820538833 |
| saint_gildas_ | -2.2464 | 47.14 | 0.193413045 | 0.412665053 |
| saint_jean_de_luz_so_ | -1.6816 | 43.395 | -0.047656788 | 0.817862454 |
| saint_malo_ | -2.0281 | 48.641 | 0.726921117 | 0.007759357 |
| saint_nazaire_ | -2.2016 | 47.267 | 0.375170239 | 0.351040853 |
| santander_ieo_spain | -3.79 | 43.461 | 0.10142801 | 0.527473977 |
| sete_france | 3.6991 | 43.398 | 0.345647295 | 0.150233231 |
| smogen_020_sweden | 11.218 | 58.354 | 1.314251541 | 8.66E-05 |
| solomonsisland_biol.lab.__usa | -76.452 | 38.317 | 0.30145199 | 0.0526569 |
| stenungsund_022_sweden | 11.832 | 58.093 | 1.499394979 | 3.59E-06 |
| sthelier_p074_uk | -2.1167 | 49.183 | 0.748837078 | 0.000303867 |
| stmarys_p231_uk | -6.3164 | 49.918 | 0.011415608 | 0.942513724 |
| stornoway_p042_uk | -6.3882 | 58.208 | 0.730993126 | 5.44E-06 |
| taranaki_076a_new_zealand | 174.03 | -39.05 | 0.535025778 | 4.47E-06 |
| tauranga_073a_new_zealand | 176.18 | -37.65 | 0.360256882 | 2.79E-05 |
| tenerife_tenerife_spain | -16.24 | 28.478 | 0.270551372 | 6.29E-05 |
| thevenard_026_australia | 133.65 | -32.146 | 0.941868989 | 1.19E-05 |
| tobermory_p223_uk | -6.0642 | 56.623 | 1.604650938 | 0.000119353 |
| toulon_france | 5.9131 | 43.117 | -0.022623544 | 0.827032347 |
| tregde_006_norway | 7.5666 | 58 | 0.578187466 | 0.00307344 |
| trieste_270061_italy | 13.75 | 45.65 | 0.362841147 | 0.153255058 |
| ullapool_p043_uk | -5.1579 | 57.895 | 1.088143821 | 0.033814233 |
| valencia_valencia_spain | -0.33 | 39.46 | -0.07487651 | 0.571722693 |
| varberg1_024_sweden | 12.217 | 57.1 | 1.657078835 | 7.57E-06 |
| vardo,norway_001 | 31.1 | 70.333 | 0.323183595 | 0.125851859 |
| venezia_punta_salute_italy | 12.426 | 45.419 | 0.393996302 | 0.070594549 |
| victorharbor_027_australia | 138.62 | -35.553 | 0.761997262 | 0.011434435 |
| vigo_ieo_spain | -8.7333 | 42.233 | -0.307841645 | 0.16495687 |
| viken_025_sweden | 12.579 | 56.142 | 1.573009054 | 0.000512341 |
| villagarcia_villagarcia_spain | -8.77 | 42.601 | -0.361478189 | 0.145925153 |
| wakkanai_japan | 141.68 | 45.4 | 0.233776418 | 0.083484117 |
| weymouth_p991_uk | -2.4479 | 50.608 | 0.749896058 | 0.000385216 |
| whitby_p174_uk | -0.61417 | 54.49 | 1.360943112 | 1.66E-05 |
| wick_p035_uk | -3.0863 | 58.441 | 0.8316092 | 0.005704565 |
| williamstown_028_australia | 144.9 | -37.857 | 0.242546887 | 0.227235534 |
| workington_p217_uk | -3.5676 | 54.651 | 1.528359385 | 9.04E-05 |

Supplementary Table 4. Global trends for 99^th^ percentile surges for G-E20C for the 1950-2015 period

| **Tide Gauge** | **longitude** | **latitude** | **Trend (mm/year)** | **p value** |
| --- | --- | --- | --- | --- |
| aasiaat,greenland_001 | -52.883 | 68.71 | -0.269286883 | 0.508155285 |
| abashiri_japan | 144.28 | 44.017 | 0.289917273 | 0.140391319 |
| aberdeen_p038_uk | -2.0745 | 57.143 | 0.742628519 | 0.096149182 |
| adak,alaska_040a_usa | -176.63 | 51.863 | 0.294904909 | 0.26106314 |
| ajaccio_aspretto_france | 8.7628 | 41.923 | -0.297607559 | 0.161878251 |
| alameda_navalairstation__usa | -122.3 | 37.772 | -0.13557656 | 0.307914103 |
| alcudia_alcudia_spain | 3.1392 | 39.835 | 0.102590332 | 0.191251126 |
| ancona_ancona_italy | 13.506 | 43.625 | -0.413786494 | 0.131759286 |
| andenes_001 | 16.15 | 69.317 | 0.992916923 | 0.037277825 |
| angra_heroismo_b_215b_portugal | -27.24 | 38.65 | -0.299848901 | 0.166461972 |
| annapolis_navalacademy__usa | -76.482 | 38.983 | 0.187842654 | 0.622071674 |
| apalachicola_usa | -84.982 | 29.727 | -0.778398589 | 0.074361038 |
| arcachon_eyrac_ | -1.1636 | 44.665 | -0.144699899 | 0.729809973 |
| arena_cove,_ca_573a_usa | -123.71 | 38.913 | -0.535027146 | 0.160596787 |
| astoria,or_572a_usa | -123.77 | 46.208 | -0.210168469 | 0.61199461 |
| atlantic_city_264a_usa | -74.418 | 39.355 | 0.34055609 | 0.342401971 |
| auckland_070a_new_zealand | 174.77 | -36.85 | 0.042431448 | 0.784832943 |
| ayukawa_japan | 141.5 | 38.3 | 0.118690985 | 0.547251457 |
| bakar_bakar_I_croatia | 14.533 | 45.3 | -0.087363301 | 0.814157996 |
| baltimore_usa | -76.578 | 39.267 | 0.218009235 | 0.413878392 |
| bangor_p662_uk | -5.6695 | 54.665 | 0.835965188 | 0.039485087 |
| barcelona_barcelona_spain | 2.163 | 41.342 | -0.120519413 | 0.522918379 |
| barharbor,frenchmanbay,me_usa | -68.205 | 44.392 | 0.040090053 | 0.893631964 |
| barmouth_p923_uk | -4.0452 | 52.719 | 0.477135342 | 0.287466637 |
| barseback_001_sweden | 12.903 | 55.756 | 1.158130631 | 0.015073634 |
| bayonne_boucau_ | -1.5148 | 43.527 | -0.419733347 | 0.213088533 |
| bilbao_bilbao_spain | -3.05 | 43.357 | -0.666950365 | 0.018139069 |
| bonanza_bonanza_spain | -6.34 | 36.8 | -0.699177702 | 0.049074417 |
| boston,ma_741a_usa | -71.052 | 42.355 | -0.102185903 | 0.794205139 |
| boulogne_sur_mer_ | 1.5777 | 50.727 | 0.751555303 | 0.168018248 |
| bournemouth_p988_uk | -1.8749 | 50.714 | 0.318479047 | 0.143014888 |
| brest_ | -4.5 | 48.383 | -0.274150667 | 0.440500207 |
| bridgeport_usa | -73.182 | 41.173 | -0.197389623 | 0.577764642 |
| cadiz_cadi_spain | -6.2833 | 36.533 | -0.21611059 | 0.511564849 |
| cagliari_cagliari_italy | 9.1143 | 39.21 | -0.452674949 | 0.001110845 |
| calais_ | 1.8677 | 50.969 | 0.780012437 | 0.169103456 |
| cambridgeii_usa | -76.068 | 38.573 | -0.094057368 | 0.781806796 |
| capemay_usa | -74.96 | 38.968 | -0.150485905 | 0.719905493 |
| cape_ferguson_343a_australia | 147.06 | -19.277 | 0.253656476 | 0.377902143 |
| cape_may,nj_746a_usa | -74.96 | 38.968 | 0.025652187 | 0.923782834 |
| carloforte_carloforte_italy | 8.3095 | 39.148 | -0.31381565 | 0.171679624 |
| carnarvon_008_australia | 113.66 | -24.884 | 0.728727247 | 0.006315586 |
| cascais_209a_portugal | -9.4167 | 38.692 | -0.25930526 | 0.39771643 |
| casey_130a_australia | 110.53 | -66.283 | 0.573909307 | 0.032494932 |
| castletownsend_835a_ireland | -9.1833 | 51.533 | 0.196830256 | 0.575958621 |
| cedarkeyii_usa | -83.032 | 29.135 | -0.46241909 | 0.289205049 |
| ceuta_ceta_spain | -5.3167 | 35.9 | -0.291589417 | 0.101935825 |
| charleston,or_575a_usa | -124.32 | 43.345 | -0.053579948 | 0.793700071 |
| chesapeake_bbt_749a_usa | -76.113 | 36.967 | 0.344264658 | 0.243882884 |
| choshigyoko_japan | 140.87 | 35.75 | 0.036456815 | 0.808744118 |
| civitavecchia_civitavecchia_italy | 11.79 | 42.094 | -0.187795867 | 0.545113104 |
| clearwater_bch,fl_773a_usa | -82.832 | 27.977 | 0.940125154 | 0.011058384 |
| concarneau_ | -3.9072 | 47.874 | 0.243939977 | 0.401510269 |
| cordova_b,alaska_583b_usa | -145.75 | 60.558 | 0.348786902 | 0.160665062 |
| corpus_cristi,tx_770a_usa | -97.217 | 27.58 | 0.451373866 | 0.021431307 |
| crescent_city,ca_556a_usa | -124.18 | 41.745 | -0.390952632 | 0.219937747 |
| cuxhaven_germany | 8.7167 | 53.867 | 2.90569237 | 0.030269244 |
| dalian_a_630a_china | 121.66 | 38.925 | -0.440069048 | 0.023323036 |
| dauphin_island,_al_763a_usa | -88.075 | 30.25 | 0.516787475 | 0.123258513 |
| davis,australia_001 | 78.5 | -68 | 0.708517649 | 0.006781355 |
| davis_173a_australia | 77.967 | -68.45 | 0.224367206 | 0.175255547 |
| delfzijl_del_nl | 6.9331 | 53.326 | 2.907258717 | 0.012235095 |
| denhelder_hel_nl | 4.7464 | 52.965 | 1.368055324 | 0.055895506 |
| devonport_p002_uk | -4.1852 | 50.368 | -0.167057579 | 0.247536958 |
| dieppe_ | 1.0845 | 49.929 | 0.474091064 | 0.458617471 |
| donges_ | -2.0883 | 47.306 | 0.703317749 | 0.225218688 |
| dover_p012_uk | 1.3181 | 51.117 | 1.452927273 | 0.023054887 |
| duck_pier,nc_260a_usa | -75.74 | 36.183 | 0.029598935 | 0.899441828 |
| dumont_d_urville_189a_france | 140.01 | -66.662 | 0.460587037 | 0.196258881 |
| dunkerque_ | 2.3667 | 51.048 | 0.578503407 | 0.313436935 |
| dutch_harbor_b,ak_041b_usa | -166.54 | 53.88 | 0.211384289 | 0.415111742 |
| esbjerg_130121_denmark | 8.4333 | 55.467 | 0.451415894 | 0.71378123 |
| felixstowe_p204_uk | 1.3484 | 51.957 | 1.245492181 | 0.213795381 |
| fernandina_beach_240a_usa | -81.467 | 30.672 | 0.164512259 | 0.831917021 |
| ferrol_ferrol_spain | -8.326 | 43.463 | -0.430010746 | 0.200106745 |
| fishguard_p055_uk | -4.9833 | 52.014 | 0.580897199 | 0.079136022 |
| flores,santa_cruz_210a_portugal | -31.168 | 39.378 | -0.12643695 | 0.485080417 |
| fortmyers_usa | -81.871 | 26.648 | 0.622960289 | 0.002398458 |
| fortpulaski_usa | -80.902 | 32.033 | 0.295854487 | 0.354262632 |
| fort_pulaski,ga_752a_usa | -80.902 | 32.033 | 0.11981273 | 0.750197966 |
| freeport_usa | -95.308 | 28.948 | 0.1221329 | 0.724565089 |
| fridayharbor_ocean.labs.__usa | -123.01 | 48.547 | -0.287179818 | 0.333714372 |
| fuerteventura_fuerteventura_spain | -13.85 | 28.5 | 0.222305061 | 4.11E-07 |
| fukaura_japan | 139.93 | 40.65 | 0.073975217 | 0.757405962 |
| funchal_b_218b_portugal | -16.907 | 32.64 | -0.034580371 | 0.905980498 |
| galveston,pier_21_775a_usa | -94.79 | 29.287 | 0.268296779 | 0.374690781 |
| gijon_gijon_spain | -5.698 | 43.558 | -0.497370485 | 0.207559717 |
| gisborne_b_078b_new_zealand | 178.03 | -38.683 | -0.004947694 | 0.976640346 |
| gladstone_p234_uk | -3.018 | 53.45 | 1.208078699 | 0.006915367 |
| gloucesterpoint_usa | -76.5 | 37.247 | -0.147109379 | 0.730189949 |
| goteborgtorshamnen_005_sweden | 11.8 | 57.683 | 1.595225147 | 0.0173879 |
| grandisle_usa | -89.957 | 29.263 | 0.591032375 | 0.016743774 |
| hachinohe_japan | 141.53 | 40.533 | 0.013022769 | 0.930810482 |
| hakodate_japan | 140.73 | 41.783 | 0.205270105 | 0.198563096 |
| hamada_japan | 132.07 | 34.9 | -0.348249943 | 0.22096469 |
| hanasaki_japan | 145.57 | 43.283 | -0.039457375 | 0.880630986 |
| heimsjoe_001_norway | 9.1014 | 63.425 | 0.657234628 | 0.122213337 |
| heysham_p050_uk | -2.9204 | 54.032 | 0.613495722 | 0.325828588 |
| hoekvanholla_hvh_nl | 4.12 | 51.977 | 1.622348261 | 0.018075044 |
| hong_kong_b_329b_china | 114.2 | 22.3 | -0.142896122 | 0.688464841 |
| honningsvaag_002_norway | 25.973 | 70.98 | 0.879361947 | 0.002549173 |
| hornbaek_838a_denmark | 12.467 | 56.1 | 1.181308293 | 0.049715195 |
| horta,azores_212a_portugal | -28.622 | 38.533 | -0.120011238 | 0.50120839 |
| huelva_huelva_spain | -6.834 | 37.132 | -0.54039135 | 0.378282356 |
| humboldt_bay,_ca_576a_usa | -124.22 | 40.767 | -0.486752291 | 0.129348347 |
| ibiza_ibiza_spain | 1.4497 | 38.911 | 0.062483783 | 0.650013342 |
| ilfracombe_p061_uk | -4.1109 | 51.211 | -0.019945857 | 0.942398565 |
| ilulissat,greenland_001 | -51.1 | 69.217 | 0.001111156 | 0.997340334 |
| imperia_imperia_italy | 8.0188 | 43.878 | -0.248863853 | 0.079468348 |
| jackson_403a_new_zealand | 168.62 | -43.983 | 0.296420409 | 0.196897927 |
| juneau_usa | -134.41 | 58.298 | -0.059819934 | 0.733990507 |
| kanmen_a_632a_china | 121.28 | 28.088 | 0.149706851 | 0.666057606 |
| ketchikan,ak_571a_usa | -131.63 | 55.333 | 0.220497797 | 0.311809858 |
| kinlochbervi_p918_uk | -5.0504 | 58.457 | 0.8822805 | 0.024065664 |
| kiptopekebeach_usa | -75.988 | 37.165 | 0.068087297 | 0.836025092 |
| kodiak_isl.,alaska_039a_usa | -152.51 | 57.732 | 0.16237959 | 0.382517456 |
| kungsvik_009_sweden | 11.127 | 58.997 | 1.238176365 | 0.031935658 |
| kushiro_japan | 144.38 | 42.967 | 0.111969315 | 0.606573853 |
| l._cornwallis_i._705a_canada | -96.95 | 75.383 | 0.803649163 | 0.000269018 |
| las_palmas_laspalmas_spain | -15.412 | 28.141 | 0.372220432 | 8.93E-14 |
| la_coruna_830a_spain | -8.4 | 43.367 | -0.398786494 | 0.130258923 |
| la_rochelle_la_palli_ | -1.2206 | 46.158 | -0.168165346 | 0.614116401 |
| leith_p034_uk | -3.1817 | 55.99 | 0.523345623 | 0.110254139 |
| lerwick_p041_uk | -1.1333 | 60.15 | 0.170632241 | 0.486910115 |
| les_sables_d_olonne_ | -1.7935 | 46.497 | -0.37960775 | 0.30149637 |
| lewes,de_747a_usa | -75.12 | 38.782 | 0.26210035 | 0.545533642 |
| le_conquet_ | -4.7807 | 48.359 | 0.250364004 | 0.176785219 |
| le_crouesty_ | -2.8952 | 47.543 | -0.647921423 | 0.211620798 |
| le_havre_ | 0.106 | 49.482 | 0.75579924 | 0.073186273 |
| lowestoft_p024_uk | 1.7508 | 52.473 | 0.211842769 | 0.650894292 |
| lusi_a_633a_china | 121.62 | 32.133 | 1.39166896 | 0.000263718 |
| lyttelton_667a_new_zealand | 172.72 | -43.6 | 0.843780655 | 0.001108981 |
| maaloey_003_norway | 5.1133 | 61.934 | 0.901360898 | 0.098335275 |
| mahon_mahon_spain | 4.2706 | 39.893 | -0.034131753 | 0.595260646 |
| maizuru_japan | 135.38 | 35.467 | 0.066627085 | 0.669670756 |
| malin_head_834a_ireland | -7.3333 | 55.367 | 0.957610597 | 0.002396793 |
| maniitsoq,greenland_001 | -52.905 | 65.413 | 0.175188746 | 0.579931722 |
| marsden_point_398a_new_zealand | 174.5 | -35.833 | -0.115163832 | 0.32225098 |
| marseille_france | 5.35 | 43.3 | 0.075927511 | 0.58849436 |
| massacre_bay,ak_550a_usa | 173.2 | 52.833 | -0.397974885 | 0.09487204 |
| mawson_177a_australia | 62.883 | -67.6 | 0.769822721 | 0.011372707 |
| mayport,fl_753a_usa | -81.432 | 30.395 | -0.091794044 | 0.768083892 |
| milfordhaven_p056_uk | -5.0143 | 51.702 | 0.449360363 | 0.161537612 |
| millport_p049_uk | -4.9058 | 55.75 | 2.071646892 | 0.001630272 |
| miyako_japan | 141.98 | 39.65 | 0.116225408 | 0.451089232 |
| monaco_port_hercule_france | 7.4215 | 43.729 | -0.239858419 | 0.226068994 |
| montauk,ny_279a_usa | -71.96 | 41.048 | -0.351165917 | 0.26749472 |
| morayfirth_p207_uk | -4.0022 | 57.599 | 0.565694662 | 0.14091667 |
| mumbles_p932_uk | -3.9754 | 51.57 | 0.746571712 | 0.0228771 |
| nantucketisland_usa | -70.097 | 41.285 | 0.034734781 | 0.951441476 |
| napier_668a_new_zealand | 176.92 | -39.483 | 0.058329032 | 0.622390975 |
| napoli_napoli_italy | 14.269 | 40.841 | -0.427618061 | 0.039272098 |
| neah_bay,wa_558a_usa | -124.62 | 48.368 | 0.58378382 | 0.026624978 |
| nelson_077a_new_zealand | 173.27 | -41.267 | 0.093010355 | 0.58126009 |
| newhaven_p011_uk | 0.05703 | 50.782 | 0.466217419 | 0.238108539 |
| newlondon_usa | -72.09 | 41.361 | -0.264465737 | 0.43496521 |
| newlyn_p001_uk | -5.5417 | 50.102 | -0.226506035 | 0.344839758 |
| newport,ri_253a_usa | -71.327 | 41.505 | -0.08693046 | 0.834687753 |
| newport_usa | -71.327 | 41.505 | 0.324966837 | 0.45644486 |
| newyork_thebattery__usa | -74.014 | 40.701 | 0.154813115 | 0.683599363 |
| new_london,ct_744a_usa | -72.087 | 41.355 | 0.082588211 | 0.86007733 |
| new_york,ny_745a_usa | -74.015 | 40.7 | -0.137215848 | 0.721292865 |
| nice_france | 7.2853 | 43.696 | 0.153404392 | 0.347224154 |
| nome,ak_595a_usa | -165.43 | 64.5 | 2.514762855 | 0.048495061 |
| northshields_p032_uk | -1.4398 | 55.007 | 1.275326013 | 0.000231927 |
| noto_japan | 137.15 | 37.5 | 0.034378877 | 0.929303307 |
| ofunato_japan | 141.72 | 39.067 | -0.175666298 | 0.297605581 |
| onahama_japan | 140.9 | 36.933 | 0.242653819 | 0.304085266 |
| ortona_ortona_italy | 14.415 | 42.356 | -0.0122318 | 0.957202268 |
| oslo_004_norway | 10.734 | 59.909 | 1.734005167 | 0.008830155 |
| otaru_japan | 141 | 43.2 | 0.370940938 | 0.151046667 |
| palma_de_mallorca_palmademallorca_spain | 2.6375 | 39.56 | -0.464177613 | 0.024772701 |
| panama_city_beach_761a_usa | -85.88 | 30.213 | 0.624331474 | 0.01788411 |
| pensacola,fl_762a_usa | -87.213 | 30.403 | 0.940884221 | 0.000347048 |
| ponta_delgada_211a_portugal | -25.672 | 37.735 | -0.195243545 | 0.151068492 |
| portellen_p202_uk | -6.1901 | 55.627 | 1.179958108 | 0.042152786 |
| porterin_p919_uk | -4.7681 | 54.085 | 1.104257277 | 0.094929701 |
| portland,s.aus._129a_australia | 141.6 | -38.333 | 0.576715708 | 0.022571247 |
| portland_maine__usa | -70.247 | 43.657 | -0.077135011 | 0.835610578 |
| portlincoln_023_australia | 135.86 | -34.72 | 0.033257367 | 0.929284783 |
| portlonsdale_017_australia | 138.5 | -35.099 | 0.180965176 | 0.606444914 |
| porto_torres_porto+torres_italy | 8.4039 | 40.842 | -0.371905964 | 0.122836067 |
| portpatrick_p063_uk | -5.12 | 54.843 | 0.846765603 | 0.063727071 |
| portpirie_024_australia | 138.01 | -33.177 | 0.644835662 | 0.131881722 |
| portrush_p935_uk | -6.6568 | 55.207 | 0.659706202 | 0.283262553 |
| portsmouth_p008_uk | -1.1118 | 50.803 | 0.685887693 | 0.034266002 |
| port_angeles,_wa_584a_usa | -123.44 | 48.125 | 0.069206926 | 0.832762157 |
| port_bloc_ | -1.0616 | 45.569 | -0.036606954 | 0.915406808 |
| port_camargue_france | 4.1264 | 43.52 | 0.102302213 | 0.2296289 |
| port_orford,_or_557a_usa | -124.5 | 42.74 | 0.129098237 | 0.637072629 |
| port_stanvac_100a_australia | 138.47 | -35.108 | 0.17647805 | 0.639017395 |
| port_tudy_ | -3.4459 | 47.644 | -0.549308766 | 0.044876508 |
| port_vendres_france | 3.1075 | 42.52 | 0.092066896 | 0.732471035 |
| prudhoe_bay,_ak_579a_usa | -148.53 | 70.4 | -0.288647492 | 0.516051986 |
| qaqortoq,greenland_001 | -46.033 | 60.717 | -0.167338323 | 0.586496162 |
| ravenna_ravenna_italy | 12.283 | 44.492 | -0.313718745 | 0.43923726 |
| reedypoint_usa | -75.573 | 39.558 | -0.151008588 | 0.74477955 |
| ringhals_016_sweden | 12.113 | 57.25 | 1.508715632 | 0.016159527 |
| roervik_005_norway | 11.23 | 64.859 | 0.543030988 | 0.080263184 |
| roscoff_ | -3.9657 | 48.718 | -0.487573581 | 0.1464303 |
| sabine_pass_n,tx_766a_usa | -93.87 | 29.73 | 1.052750635 | 0.030523122 |
| sagunto_sagunto_spain | -0.206 | 39.634 | -0.269520271 | 0.156931288 |
| saint_gildas_ | -2.2464 | 47.14 | -0.234660844 | 0.503564694 |
| saint_jean_de_luz_so_ | -1.6816 | 43.395 | -0.199313123 | 0.537840347 |
| saint_malo_ | -2.0281 | 48.641 | 0.409086339 | 0.272411993 |
| saint_nazaire_ | -2.2016 | 47.267 | -0.071827072 | 0.908514891 |
| sakai_japan | 133.25 | 35.55 | 0.107794953 | 0.66921651 |
| sandyhook_usa | -74.009 | 40.467 | 0.265456902 | 0.525341323 |
| santander_ieo_spain | -3.79 | 43.461 | -0.221266672 | 0.442374914 |
| seattle_usa | -122.34 | 47.603 | -0.548394623 | 0.012249712 |
| seldovia_usa | -151.72 | 59.44 | 0.845216334 | 2.17E-05 |
| sete_france | 3.6991 | 43.398 | 0.257518182 | 0.487162372 |
| seward_c,ak_560c_usa | -149.43 | 60.12 | -0.299856717 | 0.362130688 |
| sewellspoint,hamptonroads_usa | -76.33 | 36.947 | -0.173582171 | 0.63889783 |
| shanwei_641a_china | 115.35 | 22.75 | 0.012175098 | 0.976843992 |
| shimokita_japan | 141.23 | 41.367 | -0.195764895 | 0.342342592 |
| sitka,ak_559a_usa | -135.34 | 57.052 | 0.828441445 | 4.78E-05 |
| skagway_usa | -135.33 | 59.45 | 0.553283939 | 0.021161604 |
| smogen_020_sweden | 11.218 | 58.354 | 1.536717552 | 0.002250455 |
| solomonsisland_biol.lab.__usa | -76.452 | 38.317 | 0.269024075 | 0.268456395 |
| springmaidpier_usa | -78.918 | 33.655 | -0.129257963 | 0.640856758 |
| st.paulisland_001 | 77.583 | -38.717 | -0.041130149 | 0.841290723 |
| st.petersburg_usa | -82.627 | 27.761 | 1.213479939 | 0.00256955 |
| st._augustine,fl_262a_usa | -81.262 | 29.857 | -0.027755351 | 0.951542238 |
| st._john_s_b_276b_canada | -52.7 | 47.567 | 0.433523979 | 0.117727958 |
| st._petersburg,_fl_759a_usa | -82.627 | 27.76 | 1.318829566 | 0.000462628 |
| stenungsund_022_sweden | 11.832 | 58.093 | 1.401046058 | 0.006395553 |
| sthelier_p074_uk | -2.1167 | 49.183 | 0.643681265 | 0.003814464 |
| stmarys_p231_uk | -6.3164 | 49.918 | -0.401730868 | 0.029817494 |
| stornoway_p042_uk | -6.3882 | 58.208 | 0.844561372 | 0.001523863 |
| taranaki_076a_new_zealand | 174.03 | -39.05 | 0.301407978 | 0.16113236 |
| tauranga_073a_new_zealand | 176.18 | -37.65 | 0.155480303 | 0.107573806 |
| tenerife_tenerife_spain | -16.24 | 28.478 | 0.363435563 | 8.25E-05 |
| thevenard_026_australia | 133.65 | -32.146 | 0.596210695 | 0.076690325 |
| timaru_665a_new_zealand | 171.25 | -44.383 | 0.440131431 | 0.004868797 |
| tobermory_p223_uk | -6.0642 | 56.623 | 1.015196398 | 0.074635848 |
| toulon_france | 5.9131 | 43.117 | -0.126512505 | 0.455769978 |
| tregde_006_norway | 7.5666 | 58 | 0.415200171 | 0.090969462 |
| trieste_270061_italy | 13.75 | 45.65 | 0.284301565 | 0.215307358 |
| ullapool_p043_uk | -5.1579 | 57.895 | 1.693616023 | 0.001499194 |
| unalaska_usa | -166.54 | 53.88 | 0.524306639 | 0.019555493 |
| valdez,ak_562a_usa | -146.36 | 61.125 | 0.460310457 | 0.040782836 |
| valencia_valencia_spain | -0.33 | 39.46 | 0.054701843 | 0.774787807 |
| varberg1_024_sweden | 12.217 | 57.1 | 1.762018816 | 0.003804714 |
| vardo,norway_001 | 31.1 | 70.333 | 0.556321717 | 0.056534358 |
| venezia_punta_salute_italy | 12.426 | 45.419 | 0.194500199 | 0.402956486 |
| victorharbor_027_australia | 138.62 | -35.553 | -0.280583956 | 0.493036189 |
| victoria,bc_543a_canada | -123.37 | 48.422 | 0.114404703 | 0.627292599 |
| vigo_ieo_spain | -8.7333 | 42.233 | -0.614990438 | 0.061793596 |
| viken_025_sweden | 12.579 | 56.142 | 1.312549197 | 0.04898695 |
| villagarcia_villagarcia_spain | -8.77 | 42.601 | -0.96200723 | 0.011011673 |
| wakkanai_japan | 141.68 | 45.4 | 0.05790847 | 0.756394727 |
| washingtondc_usa | -77.022 | 38.873 | 0.181992571 | 0.618733265 |
| wellington_071a_new_zealand | 174.78 | -41.283 | 0.311294464 | 0.10684153 |
| weymouth_p991_uk | -2.4479 | 50.608 | 0.450016284 | 0.083798854 |
| whitby_p174_uk | -0.61417 | 54.49 | 0.470289736 | 0.217079214 |
| wick_p035_uk | -3.0863 | 58.441 | 0.856303568 | 0.02614382 |
| willapa_bay,_wa_564a_usa | -123.97 | 46.708 | -0.259172373 | 0.515652674 |
| willetspoint_usa | -73.782 | 40.793 | -0.836752167 | 0.082578473 |
| williamstown_028_australia | 144.9 | -37.857 | -0.54563894 | 0.029362833 |
| woods_hole,ma_742a_usa | -70.672 | 41.523 | 0.109431138 | 0.782761672 |
| workington_p217_uk | -3.5676 | 54.651 | 1.210999558 | 0.02247674 |
| xiamen_376a_china | 118.07 | 24.45 | -0.325714222 | 0.227572705 |
| yakutat_usa | -139.74 | 59.548 | -0.037959326 | 0.915427426 |

Supplementary Table 5. Global trends for 99^th^ percentile surges for seven datasets for the 1980-2010 period

| **Tide Gauge** | **longitude** | **latitude** | **Obs** | **G-20CR** | **G-E20C** | **G-EInt** | **G-Merra** | **G-E5** | **G-Ens** |
| --- | --- | --- | --- | --- | --- | --- | --- | --- | --- |
| abashiri_japan | 144.28 | 44.017 | 0.41 | 0.44 | 0.92 | 0.24 | 1.43 | -0.28 | 1.11 |
| adak,alaska_040a_usa | -176.63 | 51.863 | -0.35 | 0.28 | 1.03 | -0.34 | 0.44 | -0.12 | 0.19 |
| alameda_navalairstation__usa | -122.3 | 37.772 | 0.08 | 0.03 | -0.01 | -1.15 | -0.75 | -0.45 | -0.85 |
| annapolis_navalacademy__usa | -76.482 | 38.983 | 1.10 | -0.26 | 0.72 | -0.43 | -1.08 | -0.51 | -0.65 |
| astoria,or_572a_usa | -123.77 | 46.208 | -4.12 | 0.35 | 0.65 | 0.92 | -0.56 | -0.02 | -0.25 |
| atlantic_city_264a_usa | -74.418 | 39.355 | 0.76 | 1.66 | 1.41 | -1.59 | 1.37 | 0.22 | 0.51 |
| ayukawa_japan | 141.5 | 38.3 | 0.15 | 1.68 | 1.07 | 0.51 | 1.25 | 0.63 | 0.52 |
| bakar_bakar_I_croatia | 14.533 | 45.3 | -2.09 | -0.79 | 0.48 | 0.97 | 0.69 | -0.15 | 0.33 |
| baltimore_usa | -76.578 | 39.267 | 1.36 | -0.55 | 0.91 | -1.21 | -1.28 | -0.60 | -0.75 |
| bayonne_boucau_ | -1.5148 | 43.527 | -2.41 | 0.37 | -0.10 | -1.47 | -0.50 | -1.51 | -0.54 |
| boston,ma_741a_usa | -71.052 | 42.355 | -0.13 | 2.66 | 1.41 | 1.83 | 1.76 | 1.86 | 2.02 |
| brest_ | -4.5 | 48.383 | -2.59 | 0.85 | 1.33 | 0.96 | -0.01 | -0.37 | 0.69 |
| bridgeport_usa | -73.182 | 41.173 | 1.08 | 2.10 | 1.09 | 2.30 | -1.96 | 1.18 | 0.59 |
| cambridgeii_usa | -76.068 | 38.573 | -0.03 | -1.00 | 0.41 | -1.36 | -2.01 | -1.55 | -1.34 |
| cape_may,nj_746a_usa | -74.96 | 38.968 | 2.27 | 0.11 | 0.20 | -1.77 | 0.55 | -0.20 | -0.78 |
| ceuta_ceta_spain | -5.3167 | 35.9 | -0.76 | 0.21 | 0.31 | -0.38 | 1.45 | 0.53 | 0.30 |
| charleston,or_575a_usa | -124.32 | 43.345 | -0.96 | 0.50 | -0.13 | -0.30 | 0.57 | 1.14 | 0.31 |
| charleston,sc_261a_usa | -79.925 | 32.782 | -0.84 | 1.44 | 0.83 | 0.63 | -0.27 | -0.79 | 0.27 |
| chesapeake_bbt_749a_usa | -76.113 | 36.967 | 3.39 | 1.00 | 1.05 | 1.46 | -1.68 | -2.10 | -1.33 |
| cordova_b,alaska_583b_usa | -145.75 | 60.558 | 0.37 | 1.17 | 0.90 | 0.57 | 0.95 | 1.33 | -0.34 |
| coruna_coru_spain | -8.4 | 43.367 | -0.16 | 0.84 | 0.12 | 1.21 | 0.35 | 0.82 | 0.45 |
| crescent_city,ca_556a_usa | -124.18 | 41.745 | 0.94 | 0.48 | -0.15 | 0.20 | -0.21 | -1.10 | -0.33 |
| cuxhaven_germany | 8.7167 | 53.867 | -5.41 | -6.94 | -4.25 | -4.31 | -0.92 | -5.39 | -2.75 |
| delfzijl_del_nl | 6.9331 | 53.326 | -4.06 | -3.28 | -4.59 | -3.41 | -5.34 | -5.78 | -3.41 |
| denhelder_hel_nl | 4.7464 | 52.965 | -6.59 | -4.42 | -4.10 | -5.49 | -6.23 | -6.56 | -5.54 |
| dover_p012_uk | 1.3181 | 51.117 | -2.33 | -1.42 | -1.58 | -4.10 | -2.06 | -3.61 | -1.98 |
| duck_pier,nc_260a_usa | -75.74 | 36.183 | 0.59 | 2.19 | -0.51 | -1.00 | -1.05 | -0.09 | -0.60 |
| esbjerg_130121_denmark | 8.4333 | 55.467 | -3.23 | -4.00 | -5.29 | -6.40 | -3.99 | -4.32 | -4.09 |
| fishguard_p055_uk | -4.9833 | 52.014 | -1.33 | 1.40 | -1.83 | 0.88 | 1.75 | 0.46 | 1.13 |
| fortpulaski_usa | -80.902 | 32.033 | -1.51 | 0.14 | 0.52 | 0.14 | 1.04 | -0.19 | -0.07 |
| fort_pulaski,ga_752a_usa | -80.902 | 32.033 | -1.38 | 0.26 | 1.05 | -0.34 | 0.09 | 0.04 | 0.04 |
| fukaura_japan | 139.93 | 40.65 | 0.22 | 0.09 | 0.60 | -0.49 | 1.17 | -0.30 | 0.45 |
| galveston,pier_21_775a_usa | -94.79 | 29.287 | 0.65 | 1.63 | 0.04 | 1.84 | 0.19 | -0.11 | 0.41 |
| goteborgtorshamnen_005_sweden | 11.8 | 57.683 | -2.38 | -0.87 | -3.00 | -2.38 | -1.85 | -3.02 | -2.22 |
| hachinohe_japan | 141.53 | 40.533 | 0.66 | 0.25 | 0.01 | 0.36 | -0.46 | 0.43 | 0.16 |
| hakodate_japan | 140.73 | 41.783 | 0.07 | 0.03 | 0.96 | -0.34 | 0.21 | 0.23 | 0.33 |
| hanasaki_japan | 145.57 | 43.283 | 0.60 | 0.79 | 0.78 | 0.67 | 1.77 | 0.76 | 1.36 |
| heimsjoe_001_norway | 9.1014 | 63.425 | -1.03 | 0.88 | -1.50 | -0.28 | -0.54 | -1.27 | -0.38 |
| hoekvanholla_hvh_nl | 4.12 | 51.977 | -2.46 | -4.29 | -3.00 | -5.45 | -6.17 | -4.56 | -5.47 |
| hong_kong_b_329b_china | 114.2 | 22.3 | 0.35 | -0.07 | -0.22 | -0.45 | 0.23 | -0.44 | -0.36 |
| honningsvaag_002_norway | 25.973 | 70.98 | -1.46 | -0.88 | 0.34 | -0.66 | 0.06 | -0.66 | -0.36 |
| juneau_usa | -134.41 | 58.298 | 1.07 | 0.91 | 0.04 | 0.02 | 0.08 | 0.49 | 0.38 |
| ketchikan,ak_571a_usa | -131.63 | 55.333 | -0.24 | 1.99 | -0.24 | 1.12 | 0.26 | 0.21 | 0.29 |
| kungsvik_009_sweden | 11.127 | 58.997 | -2.56 | -1.35 | -2.10 | -1.59 | -1.89 | -0.62 | -1.17 |
| kushiro_japan | 144.38 | 42.967 | 0.30 | 1.51 | 0.24 | 0.58 | 0.70 | -0.06 | 0.70 |
| la_coruna_830a_spain | -8.4 | 43.367 | -0.30 | 0.31 | 0.13 | 1.57 | 0.21 | 0.82 | 0.36 |
| lerwick_p041_uk | -1.1333 | 60.15 | 1.30 | 1.32 | -0.64 | 0.03 | -0.16 | -0.84 | 0.38 |
| lewes,de_747a_usa | -75.12 | 38.782 | 4.21 | -1.22 | 0.72 | 1.64 | -1.02 | 0.42 | 0.63 |
| le_conquet_ | -4.7807 | 48.359 | -1.66 | 0.62 | -0.22 | -0.58 | -0.50 | -0.02 | -0.65 |
| le_havre_ | 0.106 | 49.482 | -3.93 | -0.07 | 0.17 | 0.08 | -0.17 | -1.42 | -0.88 |
| lowestoft_p024_uk | 1.7508 | 52.473 | 0.54 | 0.16 | -3.11 | -3.69 | -3.55 | -2.64 | -2.78 |
| maaloey_003_norway | 5.1133 | 61.934 | -1.03 | 0.02 | -2.14 | -0.68 | -0.57 | -0.60 | -0.43 |
| maizuru_japan | 135.38 | 35.467 | 0.17 | 0.66 | -0.01 | 0.32 | -0.30 | -0.35 | 0.21 |
| milfordhaven_p056_uk | -5.0143 | 51.702 | -2.80 | -0.02 | 0.50 | -1.46 | 1.11 | -1.61 | -0.09 |
| miyako_japan | 141.98 | 39.65 | 0.74 | 0.59 | 0.80 | 0.64 | 1.02 | 0.76 | 0.88 |
| montauk,ny_279a_usa | -71.96 | 41.048 | 1.59 | -0.48 | 0.39 | 1.07 | 0.89 | 1.49 | 0.68 |
| monterey,ca_555a_usa | -121.89 | 36.605 | -0.99 | -0.63 | 0.78 | -1.81 | -0.46 | -1.29 | -0.40 |
| nantucketisland_usa | -70.097 | 41.285 | 0.44 | -1.08 | 2.84 | 1.25 | 0.74 | 0.95 | 0.31 |
| neah_bay,wa_558a_usa | -124.62 | 48.368 | -1.14 | 0.37 | 0.11 | 0.50 | 0.22 | -0.04 | -0.04 |
| newlondon_usa | -72.09 | 41.361 | 0.26 | 0.15 | 0.69 | 0.69 | 0.84 | 1.14 | 1.10 |
| newport,ri_253a_usa | -71.327 | 41.505 | 0.97 | 0.39 | 1.74 | -0.36 | 1.06 | -0.24 | 0.20 |
| newport_usa | -71.327 | 41.505 | 0.73 | 1.06 | 2.03 | -0.40 | 1.91 | 1.04 | 0.49 |
| newyork_thebattery__usa | -74.014 | 40.701 | 2.28 | 0.62 | 1.15 | 1.04 | -0.08 | -0.03 | 0.00 |
| new_london,ct_744a_usa | -72.087 | 41.355 | 0.22 | 0.25 | 1.64 | 1.15 | 0.81 | 1.13 | 0.90 |
| ofunato_japan | 141.72 | 39.067 | 0.19 | 0.39 | 0.32 | 1.10 | 0.53 | 0.64 | 0.57 |
| onahama_japan | 140.9 | 36.933 | -0.24 | 0.90 | 1.11 | 0.80 | 1.01 | 0.73 | 0.89 |
| oslo_004_norway | 10.734 | 59.909 | -2.36 | -0.64 | -0.98 | -2.01 | -1.06 | -1.06 | -1.46 |
| pensacola,fl_762a_usa | -87.213 | 30.403 | 5.16 | -0.17 | 1.38 | 0.32 | 3.33 | 2.24 | 1.44 |
| portland_maine__usa | -70.247 | 43.657 | 0.67 | 1.38 | 1.90 | 1.54 | 1.43 | 2.58 | 1.88 |
| port_tudy_ | -3.4459 | 47.644 | 0.55 | 1.02 | 0.22 | 0.69 | 0.18 | 0.20 | 0.34 |
| ringhals_016_sweden | 12.113 | 57.25 | -2.12 | -2.22 | -1.58 | -2.01 | -2.79 | -1.75 | -2.59 |
| roervik_005_norway | 11.23 | 64.859 | -0.93 | 0.17 | -0.29 | -0.44 | -0.82 | -0.24 | -0.02 |
| roscoff_ | -3.9657 | 48.718 | -1.13 | -1.57 | -0.17 | 0.15 | -1.01 | 0.63 | 0.25 |
| saint_gildas_ | -2.2464 | 47.14 | -2.24 | 2.06 | 0.46 | 0.61 | -0.06 | 0.68 | 0.29 |
| sakai_japan | 133.25 | 35.55 | 0.48 | 0.29 | 0.75 | 0.57 | 0.09 | 0.65 | 0.17 |
| sandyhook_usa | -74.009 | 40.467 | 0.29 | -0.37 | 1.52 | 0.22 | 0.08 | -0.65 | -0.09 |
| sand_point,ak_574a_usa | -160.5 | 55.337 | 0.84 | -0.58 | -0.25 | -1.22 | -0.39 | -0.22 | -0.59 |
| sanfrancisco_usa | -122.47 | 37.807 | -0.74 | -0.77 | 0.54 | -0.97 | -0.96 | -0.73 | -0.35 |
| santander_ieo_spain | -3.79 | 43.461 | -1.99 | 0.25 | 0.13 | 0.47 | 0.60 | 0.08 | 0.40 |
| seattle_usa | -122.34 | 47.603 | 0.21 | 0.78 | 0.36 | -0.19 | -0.19 | 0.53 | 0.05 |
| seldovia_usa | -151.72 | 59.44 | -2.75 | -0.67 | 0.45 | -1.59 | -0.28 | -0.81 | 0.03 |
| seward_c,ak_560c_usa | -149.43 | 60.12 | 0.08 | 1.54 | 1.26 | 0.07 | 0.71 | 0.35 | 0.60 |
| sewellspoint,hamptonroads_usa | -76.33 | 36.947 | 3.17 | 0.47 | -0.31 | 0.89 | -0.93 | 1.22 | 0.56 |
| sitka,ak_559a_usa | -135.34 | 57.052 | -0.11 | 1.31 | 0.85 | 0.30 | 1.20 | 0.91 | 1.09 |
| smogen_020_sweden | 11.218 | 58.354 | -2.56 | -2.15 | -0.29 | -1.31 | -3.14 | -2.19 | -1.83 |
| solomonsisland_biol.lab.__usa | -76.452 | 38.317 | 0.47 | -0.02 | 0.12 | -0.65 | -0.51 | 0.39 | -0.15 |
| springmaidpier_usa | -78.918 | 33.655 | 0.41 | 0.90 | 0.33 | -0.65 | 0.00 | -0.75 | -0.65 |
| st.petersburg_usa | -82.627 | 27.761 | 0.55 | -1.14 | 3.11 | -0.27 | -0.35 | 0.33 | 0.44 |
| st._john_s_b_276b_canada | -52.7 | 47.567 | 0.08 | 0.01 | -0.17 | -0.51 | -0.30 | -0.71 | -0.84 |
| st._petersburg,_fl_759a_usa | -82.627 | 27.76 | 0.62 | -0.05 | 3.54 | -0.39 | 0.21 | 1.75 | 0.41 |
| stenungsund_022_sweden | 11.832 | 58.093 | -3.08 | -1.33 | -1.56 | -2.59 | -1.64 | -3.26 | -2.71 |
| stornoway_p042_uk | -6.3882 | 58.208 | 1.12 | -1.12 | -0.40 | 1.17 | 1.43 | -2.43 | 0.11 |
| tregde_006_norway | 7.5666 | 58 | -0.72 | -0.63 | -1.07 | -0.47 | -1.09 | -1.27 | -1.42 |
| trieste_270061_italy | 13.75 | 45.65 | -2.60 | 0.80 | 0.02 | 0.61 | 0.82 | -1.99 | 0.19 |
| valdez,ak_562a_usa | -146.36 | 61.125 | 0.18 | 0.43 | 0.52 | 1.19 | 0.66 | 1.06 | 0.44 |
| victoria,bc_543a_canada | -123.37 | 48.422 | -0.86 | 1.60 | -0.33 | 0.88 | 0.10 | -0.10 | -0.21 |
| vigo_ieo_spain | -8.7333 | 42.233 | -0.71 | 0.17 | 0.77 | 1.48 | 1.53 | 1.28 | 1.29 |
| viken_025_sweden | 12.579 | 56.142 | -2.62 | -1.98 | -3.17 | -1.45 | -3.02 | -2.48 | -2.78 |
| wakkanai_japan | 141.68 | 45.4 | 0.84 | 0.96 | 0.10 | 0.31 | 1.16 | 0.39 | 0.60 |
| wellington_071a_new_zealand | 174.78 | -41.283 | -0.02 | 0.30 | 0.52 | -0.21 | -0.42 | 0.12 | 0.15 |
| willapa_bay,_wa_564a_usa | -123.97 | 46.708 | -0.03 | 0.92 | 1.27 | 0.40 | 0.10 | 2.20 | 0.10 |
| woods_hole,ma_742a_usa | -70.672 | 41.523 | 0.01 | -0.95 | 2.53 | 2.18 | 1.90 | 1.05 | 0.97 |
| yakutat_usa | -139.74 | 59.548 | -0.05 | 0.00 | 0.42 | 0.46 | -0.63 | -0.14 | 0.17 |
| fremantle_012_australia | 115.75 | -32.053 | -0.16 | 1.64 | -0.75 | -1.08 | -0.56 | -0.85 | 0.04 |
| geelong_013_australia | 144.36 | -38.147 | 1.10 | 1.35 | 0.16 | 0.39 | 0.12 | -0.28 | -0.21 |
| portadelaideouter_020_australia | 138.6 | -34.926 | 0.18 | 1.50 | 1.10 | -0.96 | -0.43 | -0.69 | 0.36 |
| portlincoln_023_australia | 135.86 | -34.72 | 0.64 | 1.10 | 2.10 | -0.88 | -0.61 | -0.81 | 0.56 |
| portpirie_024_australia | 138.01 | -33.177 | 1.03 | 2.71 | 1.03 | -2.64 | -2.93 | -1.64 | -1.25 |
| thevenard_026_australia | 133.65 | -32.146 | -1.20 | 0.90 | 1.58 | -0.21 | 0.13 | -0.63 | 0.57 |
| victorharbor_027_australia | 138.62 | -35.553 | -0.74 | 1.20 | 1.62 | -0.78 | -0.38 | -1.00 | 0.21 |
| williamstown_028_australia | 144.9 | -37.857 | 0.30 | 0.45 | 0.45 | 1.00 | 0.36 | 0.40 | 0.58 |
